# Biomimetic Nanomedicine for Senescence‐Modulated Immune Activation Enhances Immunotherapy Efficacy in Hepatocellular Carcinoma

**DOI:** 10.1002/advs.202517792

**Published:** 2025-12-23

**Authors:** Shiji Fang, Liyun Zheng, Bin Lin, Jiale Chen, Dehai Hou, Yiming Ding, Mengzhu Han, Pan Qin, Mengyuan Wang, Xiaoju Guo, Yeyu Zhang, Gaofeng Shu, Fazong Wu, Jianfei Tu, Minjiang Chen, Zhongwei Zhao, Zhuang Liu, Jiansong Ji

**Affiliations:** ^1^ Zhejiang Key Laboratory of Imaging and Interventional Medicine the Fifth Affiliated Hospital of Wenzhou Medical University Lishui 323000 China; ^2^ Institute of Functional Nano & Soft Materials (FUNSOM) Jiangsu Key Laboratory for Carbon‐Based Functional Materials & Devices Soochow University Suzhou 215123 China; ^3^ Cancer center Lishui Central Hospital the Fifth Affiliated Hospital of Wenzhou Medical University Lishui 323000 China; ^4^ Department of Radiology Lishui Central Hospital the Fifth Affiliated Hospital of Wenzhou Medical University Lishui 323000 China; ^5^ School of Pharmacy Jiamusi University Jiamusi 154007 China

**Keywords:** anticancer immunotherapy, combination therapy, hepatocellular carcinoma, nanoparticles, radiofrequency ablation

## Abstract

Tumor senescence, a double‐edged sword, can suppress tumor growth but also promote immune evasion if not properly cleared. Herein, a cell membrane‐coated ZIF‐8@MnOx nanoplatform co‐loaded with doxorubicin (DOX) and piperlongumine (PL), termed mPDZM, is developed to remodel the senescence‐mediated immune response in hepatocellular carcinoma. PL synergizes with DOX to amplify intracellular oxidative stress, which promotes both the killing of tumor cells and the clearance of senescent cells. The biomimetic ZIF‐8@MnOx nanoplatform potentiates the efficacy of DOX and PL by integrating targeted delivery, hypoxia relief, and redox homeostasis disruption. mPDZM remodels the immunosuppressive microenvironment by regulating SASP release, inducing immunogenic cell death, and activating the STING signaling pathway. In vivo, mPDZM exhibits preferential tumor accumulation and minimal systemic toxicity. mPDZM treatment leads to significant tumor suppression both in the senescent and non‐senescent tumor models. Moreover, mPDZM effectively promotes CD8^+^ T cell and NK cell infiltration, while reducing immunosuppressive Treg cells and M2‐like macrophages. In combination with anti‐PD‐L1 therapy, mPDZM further potentiates antitumor immunity and induces a robust abscopal effect against distant tumors. Collectively, these findings unveil a new paradigm that integrates senescence modulation with immune activation via a biomimetic nanotherapeutic platform and offers a promising combinatorial approach to overcome immune resistance in solid tumors.

## Graphical abstract

A multifunctional mPDZM nanoplatform is developed in this study. mPDZM integrates chemotherapy‐induced senescence, selective senolysis, and STING‐mediated immune activation. mPDZM effectively clears senescent tumor cells, remodels the tumor immune microenvironment, and enhances antitumor T‐cell responses. Combined with PD‐L1 blockade, mPDZM achieves robust local and systemic antitumor efficacy.

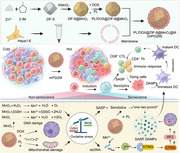

## 1 Introduction

Primary liver cancer is one of the most common and deadly malignancies worldwide, ranking as the sixth most frequently diagnosed cancer and the third leading cause of cancer‐related death.^[^ ^1^ ^]^ Hepatocellular carcinoma (HCC) is the predominant type, accounting for ≈75% of all primary liver cancer.^[^ ^2^, ^3^ ^]^ Despite advances in early screening and therapeutic modalities, more than 70% of HCC cases are diagnosed at intermediate or advanced stage, where curative strategies such as surgical resection, liver transplantation, or ablation are no longer feasible.^[^ ^3^, ^4^ ^]^ In these cases, palliative approaches such as transarterial chemoembolization, tyrosine kinase inhibitors, immune checkpoint inhibitors (ICIs), and combination therapies are often employed.^[^ ^3^, ^5^ ^]^ However, the overall response rate remains unsatisfactory due to tumor heterogeneity, intrinsic and acquired resistance, and the immunosuppressive tumor microenvironment (TME).

Recently, ICIs targeting PD‐1, PD‐L1, and CTLA‐4 have demonstrated substantial therapeutic potential in the treatment of HCC.^[^ ^6^ ^]^ By disrupting inhibitory signaling pathways that suppress T cell activation, ICIs can reinvigorate cytotoxic lymphocyte responses and facilitate immune‐mediated tumor clearance.^[^ ^7^ ^]^ When combined with anti‐angiogenic agents or chemotherapeutics, ICIs have the potential to overcome therapeutic resistance and enhance the durability of response in advanced HCC.^[^ ^8^, ^9^ ^]^ However, a substantial proportion of patients continue to exhibit limited or transient responses, highlighting the inherent complexity of tumor‐immune interactions.^[^ ^10^, ^11^ ^]^ These limitations underscore the urgent need for more rationally designed, mechanistically informed, and precisely targeted therapeutic innovations to improve clinical outcomes of intermediate or advanced HCC.

To address the limited efficacy, increasing efforts have focused on strategies to convert “cold” tumors into “hot” ones by enhancing tumor immunogenicity.^[^ ^12^, ^13^, ^14^ ^]^ Among these, the induction of immunogenic cell death (ICD) represents a particularly attractive approach.^[^ ^13^, ^14^ ^]^ ICD involves the emission of damage‐associated molecular patterns (DAMPs), including calreticulin (CRT), adenosine triphosphate (ATP), and high‐mobility group box 1 (HMGB1). These DAMPs promote the maturation of dendritic cells (DC) and enhance antigen presentation, thereby initiating robust antitumor immune responses. Notably, oxidative stress has been reported to play a critical role in the initiation and amplification of ICD.^[^ ^15^, ^16^ ^]^ Evidence has been demonstrated that the accumulation of reactive oxygen species (ROS) within tumor cells induces endoplasmic reticulum stress and facilitates CRT surface translocation.^[^ ^17^, ^18^, ^19^ ^]^ Moreover, ROS can also activate the cyclic GMP‐AMP synthase (cGAS)‐stimulator of interferon genes (STING) signaling pathway to trigger type I interferon (IFN) responses. These responses further promote dendritic cell maturation and cytotoxic T cell priming.^[^ ^20^ ^]^ Accordingly, therapeutic strategies aimed at elevating ROS levels have been proposed as a promising approach to amplify immune activation and enhance the efficacy of ICIs in tumors

In addition to ICD‐based strategies that enhance tumor immunogenicity, therapy‐induced senescence has recently emerged as a complementary immunomodulatory mechanism in cancer treatment.^[^ ^21^ ^]^ Cellular senescence is characterized by a stable arrest of the cell cycle by various cellular stresses, including DNA damage, oxidative stress, oncogene activation, and exposure to chemotherapeutic or radiotherapeutic agents. Senescent cells often exhibit resistance to death.^[^ ^22^ ^]^ Unlike apoptotic cells, senescent cells remain metabolically active and often acquire a distinctive senescence‐associated secretory phenotype (SASP), which is characterized by the secretion of pro‐inflammatory cytokines, chemokines, growth factors, and proteases.^[^ ^23^ ^]^ However, cellular senescence functions as a double‐edged sword in cancer immunity. On one hand, senescent tumor cells can enhance immune surveillance by secreting pro‐inflammatory cytokines and upregulating ligands that recruit and activate immune effector cells such as NK cells and CD8⁺ T lymphocytes.^[^ ^24^, ^25^, ^26^ ^]^ On the other hand, chronic or unresolved senescence can foster an immunosuppressive tumor microenvironment. Persistent SASP expression, including IL‐6 and IL‐8, has been associated with enhanced tumor proliferation, angiogenesis, and metastasis.^[^ ^27^ ^]^ Moreover, senescent tumor cells may promote the infiltration of immunosuppressive cell populations such as regulatory T cells (Tregs) and M2‐like tumor‐associated macrophages (TAMs).^[^ ^28^ ^]^ Prolonged senescence can also lead to the upregulation of immune checkpoint molecules such as PD‐L1, a marker that suppresses cytotoxic T cell responses and reduces ICI efficacy.^[^ ^29^ ^]^ In inflammation‐associated liver cancer models, elevated SASP‐related cytokines are strongly correlated with increased tumor burden and recurrence risk.^[^ ^30^ ^]^ Therefore, the immunological outcome of senescence is highly context‐dependent, and therapeutic strategies must be carefully designed to harness its beneficial immune‐activating effects while mitigating its potential to drive tumor immune evasion.

To resolve this paradox, a “one‐two punch” strategy has been proposed, involving sequential induction of senescence in tumor cells followed by targeted elimination of the senescent population using senolytic agents.^[^ ^31^, ^32^ ^]^ This strategy seeks to exploit the initial tumor‐suppressive effects of senescence while mitigating the long‐term pro‐tumorigenic risks. Several recent studies support this approach. Wang et al.^[^ ^32^ ^]^ showed that a combination of XL413 and AZD8055 effectively induced and cleared senescent tumor cells in liver cancer models. Similarly, Zhang et al. ^[^ ^33^ ^]^ demonstrated that tryptanthrin, a natural senescence inducer, synergized with senolytic therapy via the ROS/DNA damage response/NF‐κB/SASP pathway to inhibit HCC progression. Despite these advances, the clinical translation of senescence‐based therapy faces critical challenges. First, the clearance of senescent cells is often incomplete, leading to chronic SASP release and sustained immune suppression. Second, the immune response elicited by senescence is highly context‐dependent and often insufficient to induce durable tumor regression. Third, some senolytic agents may exhibit off‐target toxicity or lack tumor specificity. Therefore, the development of a comprehensive and mechanistically guided approach that precisely coordinates senescence induction, selective clearance of senescent cells, and effective immune stimulation is urgently needed.

Nanoparticle‐based delivery systems offer a versatile platform to address the multifaceted challenges associated with senescence‐based cancer therapy.^[^ ^34^ ^]^ By enabling the co‐delivery of multiple therapeutic agents with stimulus‐responsive control, nanocarriers enhance drug bioavailability, improve pharmacokinetic profiles, and facilitate tumor‐specific targeting, thereby minimizing off‐target effects and overcoming resistance. Among various nanocarriers, zeolitic imidazolate framework‐8 (ZIF‐8) has been extensively investigated due to its exceptional biocompatibility, high surface area, tunable pore size, and pH‐sensitive degradability.^[^ ^35^ ^]^ Notably, ZIF‐8′s facile surface functionalization allows integration with complementary materials to achieve multifunctional and stimuli‐responsive delivery. MnO_x_, which is a well‐known redox‐sensitive material, can be employed to functionalize ZIF‐8 nanoparticles.^[^ ^36^, ^37^ ^]^ Indeed, MnO_x_ readily undergoes degradation under tumor‐relevant conditions, such as elevated hydrogen peroxide levels and acidic pH, leading to the release of Mn^2+^ ions and ROS generation.^[^ ^38^ ^]^ The byproducts of MnO_x_ not only exacerbate redox stress within tumor cells but also activate the cGAS‐STING pathway, thereby potentiating antitumor immunity.^[^ ^39^ ^]^ Moreover, Mn^2+^ ions are recognized as T_1_‐weighted MRI contrast agents that enhance signal intensity for tumor imaging.^[^ ^40^ ^]^ It is worth mentioning that cloaking nanoparticles with tumor cell membrane (CM) derived from tumor cells which mimic the biological identity of the source tumor cells, has emerged as a promising approach to improve has emerged as an effective strategy to improve homotypic targeting and immune evasion.^[^ ^41^ ^]^ Thus, the development of ZIF‐8‐based hybrid nano‐system incorporating MnO_x_ and CM coatings offers a powerful and rational design to potentiate the “one‐two punch” strategy against HCC within its immunosuppressive TME.

To implement the “one‐two punch” strategy, the combination of doxorubicin (DOX) and piperlongumine (PL) appears to be optimal. DOX, a well‐established anthracycline chemotherapeutic, induces DNA double‐strand breaks and activates the p53 pathway, leading to senescence in HCC cells.^[^ ^42^, ^43^ ^]^ PL, a natural alkaloid derived from *Piper longum*, selectively eliminates senescent cells by disrupting redox homeostasis and inducing apoptosis.^[^ ^44^ ^]^ Moreover, PL has been shown to potentiate the cytotoxic effects of DOX and tyrosine kinase inhibitors such as sorafenib by promoting intracellular ROS accumulation.^[^ ^45^ ^]^ Importantly, PL restored ROS levels, which in turn triggered oxidative stress and immune activation.^[^ ^46^ ^]^ Thus, the DOX/PL combination may offer a synergistic therapeutic mechanism that both initiates senescence and selectively clears the resulting senescent tumor cells, facilitating a temporally coordinated intervention in HCC.

In this study, a multifunctional ZIF‐8‐based hybrid nano‐system was constructed by integrating a redox‐responsive MnO_X_ shell and a homologous CM coating for synergistic senescence‐based cancer therapy. DOX and PL were co‐loaded into the nanocarrier to achieve a temporally orchestrated “one‐two punch” strategy, wherein DOX induces senescence, and PL facilitates senolytic clearance. The system was systematically evaluated through a series of in vitro and in vivo experiments to characterize its physicochemical properties, drug release profiles, cytotoxicity, and immune activation. This rationally designed nanoplatform is anticipated to enhance tumor‐specific accumulation, amplify antitumor efficacy, and elicit sustained immune responses within the immunosuppressive TME of HCC.

## 2 Results and Discussion

### 2.1 Synergistic Cytotoxic and Senolytic Effects of DOX and PL

**Figure 1:**
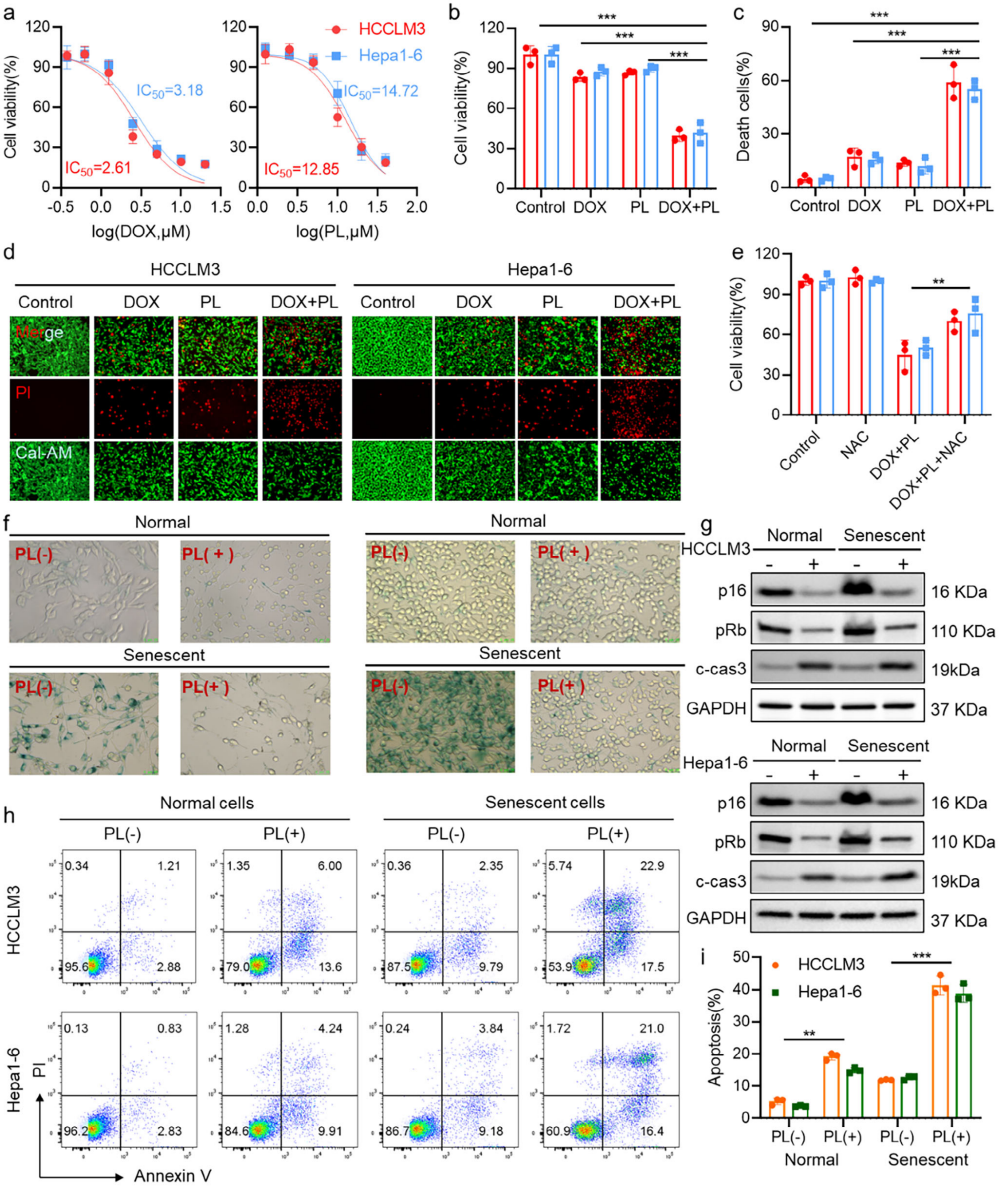
Synergistic cytotoxic and senolytic effects of DOX and PL. a) Dose‐response curves and IC_50_ values of DOX and PL against HCCLM3 and Hepa1‐6 cells. b) Cell viability of HCCLM3 and Hepa1‐6 cells treated with DOX, PL, or their combination. c) Cell death rates in DOX‐treated cells with or without PL, quantified from Calcein‐AM/PI staining images. d) Representative fluorescence images of Calcein‐AM/PI staining showing live (green) and dead (red) cells under the indicated treatments. e) Cell viability of HCCLM3 and Hepa1‐6 cells treated with DOX and PL, with or without N‐acetyl‐L‐cysteine (NAC, 5 mm) pretreatment. f) SA‐β‐gal staining images showing senescent morphology and β‐gal positivity with or without PL treatment in DOX‐induced senescent cells. g) Western blot analysis of p16I expression, Rb phosphorylation (pRb), and cleaved caspase 3 in DOX‐induced senescent HCCLM3 and Hepa1‐6 cells treated with or without PL. GAPDH served as a loading control. h) Flow cytometric analysis of Annexin V‐FITC/PI staining in DOX‐induced senescent HCCLM3 and Hepa1‐6 cells treated with or without PL. i) Quantification of apoptotic cells. Data are presented as mean ± SD (n = 3). Statistical analysis was performed using one‐way ANOVA + Tukey's test, **p < 0.01, ***p < 0.001. Note: c‐casp3 denotes cleaved caspase‐3.

To investigate the cytotoxic potential of the individual therapeutic agents of DOX and PL, dose‐dependent viability assays were performed both in HCCLM3 and Hepa1‐6 cells using the 3‐(4,5‐dimethylthiazol‐2‐yl)‐2,5‐diphenyltetrazolium bromide (MTT) method. As shown in **Figure** **1**a, both DOX and PL induced concentration‐dependent growth inhibition in the two cell lines. IC_50_ values of DOX were 1.52 µg mL^−1^ for HCCLM3 and 1.84 µg mL^−1^ for Hepa1‐6, respectively. The IC_50_ values of PL were 4.08 µg/mL and 4.67 µg/mL. To investigate the potential synergy between DOX and PL, low‐dose concentrations that individually elicited ≈15% growth inhibition were selected. MTT assay revealed that both 0.58 µg/mL DOX and 1.58 µg/mL PL exhibited only a slight reduction in cell viability of HCCLM3 and Hepa1‐6 cells. However, a markedly enhanced reduction in cell viability was observed in cells treated with the combination treatment of DOX and PL (Figure 1b). The observed enhancement in cytotoxicity supports a potent synergistic interaction between the two agents. For validation, live/dead cell staining was performed (Figure 1c,d). Treatment with low‐dose DOX or PL alone induced only limited cell death in HCCLM3 cells, with death rates of 13.77 ± 1.90% and 17.13 ± 4.98%, respectively. In contrast, upon combination treatment, a pronounced increase in death rate to 59.03 ± 9.35%. Similarly, the trend was also noted in Hepa1‐6 cells. These findings indicate that DOX and PL, even at concentrations with limited individual efficacy, can act cooperatively to induce potent cytotoxicity in HCC cells. The low‐dose synergy offers potential advantages in reducing systemic toxicity while maintaining antitumor efficacy, and thus provides a promising rationale for combination therapy strategies.

Accumulating evidence suggests that the anticancer activity of DOX and PL is mediated, at least in part, through the induction of oxidative stress.^[^ ^43^, ^45^ ^]^ Thus, redox homeostasis within the treated cells was further investigated. Flow cytometric analysis (Figure S1, Supporting Information) demonstrated that monotherapy with DOX or PL moderately increased the mean fluorescence intensity of DCF to 1.93 ± 0.31 and 2.15 ± 0.39, respectively. In contrast, co‐treatment with DOX and PL markedly elevated the DCF fluorescence intensity to 5.41 ± 0.58, which was ≈2.80‐ and 2.52‐fold higher than that observed in cells treated with DOX or PL alone, respectively. These findings were further validated in Hepa1‐6 cells, highlighting a robust and synergistic burst of ROS accumulation. Moreover, pre‐incubation with N‐acetylcysteine (NAC), a ROS scavenger, effectively attenuated the cytotoxicity induced by DOX and PL co‐treatment, as indicated by a significant increase in cell viability (Figure 1e).

Given that mitochondria are both the major source and target of oxidative stress,^[^ ^47^ ^]^ we next examined mitochondrial ROS accumulation and membrane potential integrity to clarify the role of mitochondrial dysfunction in DOX and PL‐induced cytotoxicity. Flow cytometric analysis (Figure S2, Supporting Information) revealed that mitochondrial ROS levels, as indicated by Mito‐SOX fluorescence, were significantly higher in cells co‐treated with DOX and PL compared to those treated with DOX or PL alone. Notably, co‐treatment with DOX and PL induced a marked loss of mitochondrial membrane potential, as evidenced by a significantly higher proportion of JC‐1 monomers compared to cells treated with DOX or PL alone (Figure S3, Supporting Information). Collectively, our results suggest that the enhanced cytotoxicity induced by DOX and PL co‐treatment is largely mediated by ROS‐driven mitochondrial damage.

### 2.2 Senolytic Effects of PL in DOX‐Induced Senescent Cells

It has been reported that DOX triggers senescence through DNA damage and cell cycle arrest, and PL acts as a senolytic agent that preferentially targets and clears senescent cells.^[^ ^43^, ^44^ ^]^ We therefore hypothesized that the combination of DOX and PL could achieve a “one‐two punch” strategy, where senescence is induced by DOX and cleared by PL. To test this hypothesis, DOX‐induced senescent HCC cell models were established by continuous exposure to 0.25 µg mL^−1^ DOX for 5 days (Figure S4, Supporting Information). SA‐β‐gal staining results revealed that SA‐β‐gal‐positive cells were observed after 2 days of low‐dose DOX treatment. By day 5, more than 50% of the cells exhibited SA‐β‐gal positivity. Immunofluorescence staining revealed that continuous exposure to DOX markedly increased the expression of γH2AX and p16 (Figure S5, Supporting Information), indicating a robust induction of cellular senescence upon prolonged exposure.

Importantly, PL treatment resulted in a pronounced reduction in SA‐β‐gal‐positive cells in both DOX‐induced senescent HCCLM3 and Hepa1‐6 cell lines (Figure 1f). Western blot analysis showed that PL also led to a marked reduction in the expression of p16 and phosphorylated Rb (pRb) (Figure 1g). These results suggested that PL exhibits senolytic activity in DOX‐induced senescence. It is well established that senescent cells are resistant to apoptosis, and the induction of apoptosis could serve as an effective strategy to selectively eliminate senescent cells.^[^ ^22^, ^48^ ^]^ As an apoptosis‐inducing agent,^[^ ^49^ ^]^ PL induced apoptosis in both normal and senescent HCCLM3 and Hepa1‐6 cells, as indicated by a notable increase in apoptotic cell populations. Notably, the effect was more pronounced in senescent cells (Figure 1h,i). Western blot analysis revealed that PL treatment increased the expression of cleaved‐caspase 3 in both normal and senescent cells (Figure 1g). These results highlight that PL maintains potent pro‐apoptotic activity even in the senescent state. Taken together, the combination of DOX and PL not only exerts a synergistic anticancer effect in normal HCC cells, but also serves as a “one‐two punch” strategy to induce and subsequently eliminate senescent cells in the context of HCC.

### 2.3 Synthesis and Characterization of Cell Membrane‐Coated DOX/PL@ZIF‐8@MnOx

**Figure 2:**
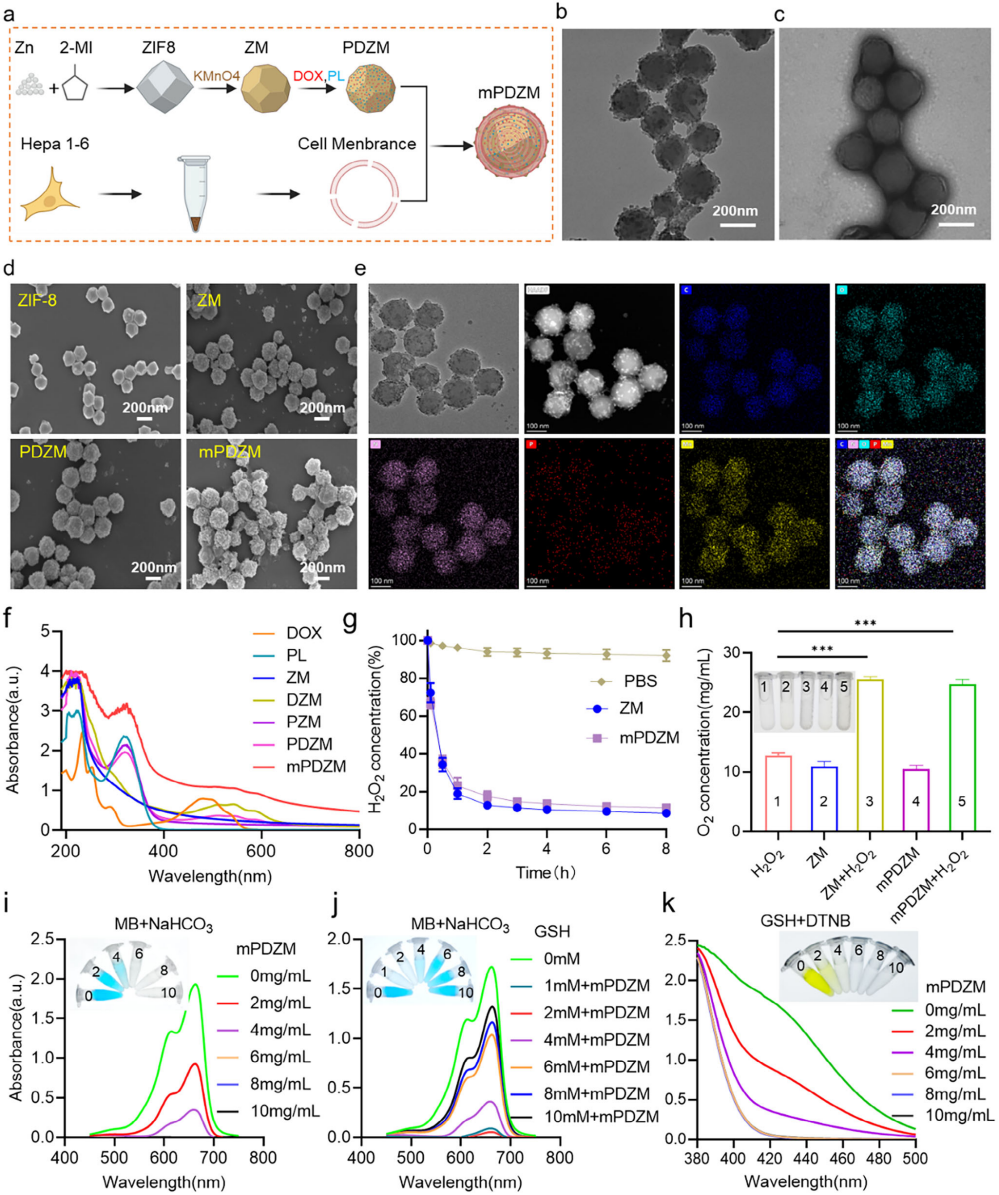
Synthesis and characterization of mPDZM nanoparticles. a) Schematic illustration of the preparation of mPDZM via sequential synthesis of ZIF‐8, surface modification with MnOx, co‐loading of DOX and PL, and coating with Hepa1‐6 cell membrane. b) TEM image of mPDZM showing uniform particle morphology. Scale bar: 200 nm. c) TEM image of mPDZM after negative staining to enhance contrast. d) SEM images of ZIF‐8, ZM, PDZM, and mPDZM. e) Elemental mapping images of mPDZM demonstrating the distribution of Mn, C, N, O, and P elements. f) UV–vis absorption spectra of different nanoparticles. g) H_2_O_2_ consumption in PBS, ZM, and mPDZM suspensions determined by UV–vis absorbance at 240 nm. h) O_2_ generation in H_2_O_2_, ZM, ZM + H_2_O_2_, mPDZM, and mPDZM + H_2_O_2_ measured using a portable dissolved oxygen meter. i) Hydroxyl radical generation detected by MB degradation in MB + NaHCO_3_ solution, monitored using UV–vis spectroscopy at 664 nm. j) MB degradation in MB + NaHCO_3_ solution in the presence of different GSH concentrations (0–10 mm). k) GSH depletion at different concentrations of mPDZM measured by the DTNB assay, monitored using UV–vis spectroscopy at 412 nm. Data are presented as mean ± SD (n = 3). Statistical analysis was performed using one‐way ANOVA + Tukey's test, *p < 0.05, **p < 0.01, ***p < 0.001.

The synthesis of DOX and PL co‐loaded ZIF‐8@MnOx coated with CM, termed DOX/PL@ZIF‐8@MnOx@CM (mPDZM), is described in **Figure** **2**a. Briefly, ZIF‐8 nanoparticles were first synthesized via a classical room‐temperature self‐assembly method by mixing zinc nitrate and 2‐methylimidazole in aqueous solution.^[^ ^50^ ^]^ Subsequently, MnOx was incorporated onto ZIF‐8 in situ deposition of KMnO_4_, yielding ZIF‐8@MnOx (ZM) nanoparticles, which exhibited characteristic Mn 2p and O 1s peaks at ≈641 eV and ≈530 eV in X‐ray photoelectron spectroscopy (XPS) spectra (Figure S6, Supporting Information). Fourier‐transform infrared (FTIR) spectra also confirmed the MnOx deposition, as evidenced by the additional peaks at ≈530 and ≈640 cm^−1^ corresponding to Mn–O vibrations (Figure S6, Supporting Information). Transmission electron microscopy (TEM) imaging revealed that upon MnOx deposition, the resulting ZM nanoparticles exhibited a distinct core–shell structure with a visibly roughened and darker outer layer, along with a slight increase in size compared to pristine ZIF‐8 (Figure S7, Supporting Information). Thereafter, DOX and PL were loaded with or without CM coated onto the surface of ZM to obtain the DOX/PL@ZIF‐8@MnOx (PDZM) and DOX/PL@ZIF‐8@MnOx@CM (mPDZM). X‐ray diffraction (XRD) patterns revealed that, similar to the other formulations (ZM, PDZM), mPDZM retained the characteristic diffraction peaks of ZIF‐8, indicating that the crystalline framework remained largely intact after MnOx deposition, drug loading, and membrane coating (Figure S6, Supporting Information).

To validate the successful coating with CM, TEM, and negatively stained TEM imaging were performed. TEM images revealed that the mPDZM particles maintained a uniform spherical morphology with a well‐defined core–shell structure, with an average particle diameter was ≈200 nm. Negatively stained TEM image showed a distinct contrast corona around each particle, indicating successful membrane coating (Figure 2b,c). Scanning electron microscopy (SEM) images revealed that mPDZM particles exhibited a rough and irregular surface texture, similar to PDZM and ZM, but with an additional diffuse outer layer, indicative of successful cell membrane coating. In contrast, pristine ZIF‐8 displayed well‐defined dodecahedral structures with smooth surfaces (Figure 2d). Moreover, elemental mapping images (Figure 2e) displayed the uniform distribution of key elements, including C, N, O, Mn, and P, within the mPDZM nanoparticles. Notably, the clear signals for P suggested the presence of membrane‐derived phospholipids and proteins, further supporting successful CM coating. Moreover, SDS‐PAGE analysis (Figure S8, Supporting Information) revealed that both CM‐coated ZM (mZM) and mPDZM exhibited protein bands consistent with those of the extracted CM, whereas the uncoated nanoparticles showed no detectable protein signal, confirming the successful formation of the biomimetic mPDZM nanoplatform.

Dynamic light scattering (DLS) analysis (Figure S9, Supporting Information) revealed that the hydrodynamic diameter of mPDZM was 230.80 ± 3.98 nm, which was comparable to that of ZM (213.37 ± 3.84 nm) and PDZM (221.33 ± 3.20 nm), but slightly larger than that of pristine ZIF‐8 (192.67 ± 3.23 nm). The zeta potential of mPDZM exhibited a moderately negative surface charge, whereas ZIF‐8, ZM, and PDZM showed positively charged surfaces. The negative surface charge of mPDZM was lower than that of free CM, further indicating partial membrane fusion and successful nanoparticle coating. UV–vis absorption spectra (Figure 2f) revealed that mPDZM exhibited characteristic peaks at ≈480 and ≈332 nm, corresponding to the absorbance signatures of DOX and PL, respectively, demonstrating successful co‐loading of both agents. Similarly, PDZM exhibited distinct absorbance peaks corresponding to both DOX and PL, while DZM showed a single peak at ≈480 nm, and PZM displayed a peak at ≈332 nm. Drug‐loading behavior evaluation showed that the encapsulation efficiency of DOX and PL was 98.97 ± 1.00% and 82.57 ± 0.68%, respectively. The loading efficiency of DOX and PL was 8.37 ± 0.08% and 7.08 ± 0.19%, respectively (Figure S10, Supporting Information). Cumulative drug release analysis indicated that both DOX, PL, and Mn^2+^ were released in a pH‐dependent manner, with significantly higher release observed under acidic conditions (pH 5.0 and 6.5) compared to pH 7.4 (Figure S11, Supporting Information). Importantly, DOX exhibited a consistently faster release profile than PL, which may result from differences in their molecular hydrophilicity and interactions with the carrier matrix. Stability assessment indicated that mPDZM maintained a relatively stable size in double‐distilled water, PBS, and 10% FBS (Figure S12, Supporting Information).

Given the intrinsic catalytic ability of MnOx to decompose hydrogen peroxide (H_2_O_2_),^[^ ^38^ ^]^ we further investigated the catalytic performance of mPDZM by measuring H_2_O_2_ consumption, oxygen generation, and hydroxyl radical generation. Upon incubation with mPDZM, the concentration of H_2_O_2_ sharply decreased within the first hour, indicating robust MnOx‐mediated catalytic activity. The rate of H_2_O_2_ consumption by mPDZM was comparable to that of PDZM, indicating that CM coating did not compromise the catalytic activity of the nanoparticles. In contrast, PBS‐treated samples showed negligible change in H_2_O_2_ levels (Figure 2 g). Moreover, mPDZM triggered robust oxygen bubble generation in H_2_O_2_ solution. The O_2_ concentration in the mPDZM‐added solution reached 24.75 ± 0.80 mg mL^−1^, which was comparable to that observed for PDZM (25.53 ± 0.53 mg mL^−1^), indicating that membrane coating did not compromise the oxygen‐generating capability. Such robust oxygen generation, which may help alleviate tumor hypoxia, has the potential to improve therapeutic outcomes. To evaluate the ability of mPDZM to catalyze the conversion of H_2_O_2_ into hydroxyl radicals via a Fenton‐like reaction, a methylene blue (MB) degradation assay was conducted. UV–vis absorption spectra showed that the characteristic peak of MB ≈664 nm gradually decreased with increasing concentrations of mPDZM. In particular, the absorbance significantly declined at higher mPDZM concentrations, indicating efficient MB degradation. To further assess the glutathione (GSH)‐responsive catalytic behavior, mPDZM was added to solutions containing different concentrations of GSH. As shown in Figure 2j, MB degradation was enhanced in the presence of a low dosage of GSH, suggesting that the catalytic performance of mPDZM was amplified in a reductive environment. At GSH concentrations above 4 mm, the absorbance began to increase, suggesting that excessive GSH may scavenge hydroxyl radicals. These results suggest that GSH not only enhances hydroxyl radical generation via MnOx‐mediated Fenton‐like reactions but may also be consumed in the process as a redox‐active substrate. To further verify the GSH‐depleting capacity of mPDZM, a DTNB assay was performed. UV–vis absorption spectra showed that the TNB absorbance at 412 nm gradually decreased with increasing concentrations of mPDZM, demonstrating effective depletion of GSH levels (Figure 2k). Collectively, these findings highlight the multifunctional catalytic and redox‐modulatory properties of mPDZM, underscoring its potential as a therapeutic platform for cancer treatment.

### 2.4 Cellular Uptake, Cytotoxicity, and Elimination of Senescent Cells

**Figure 3:**
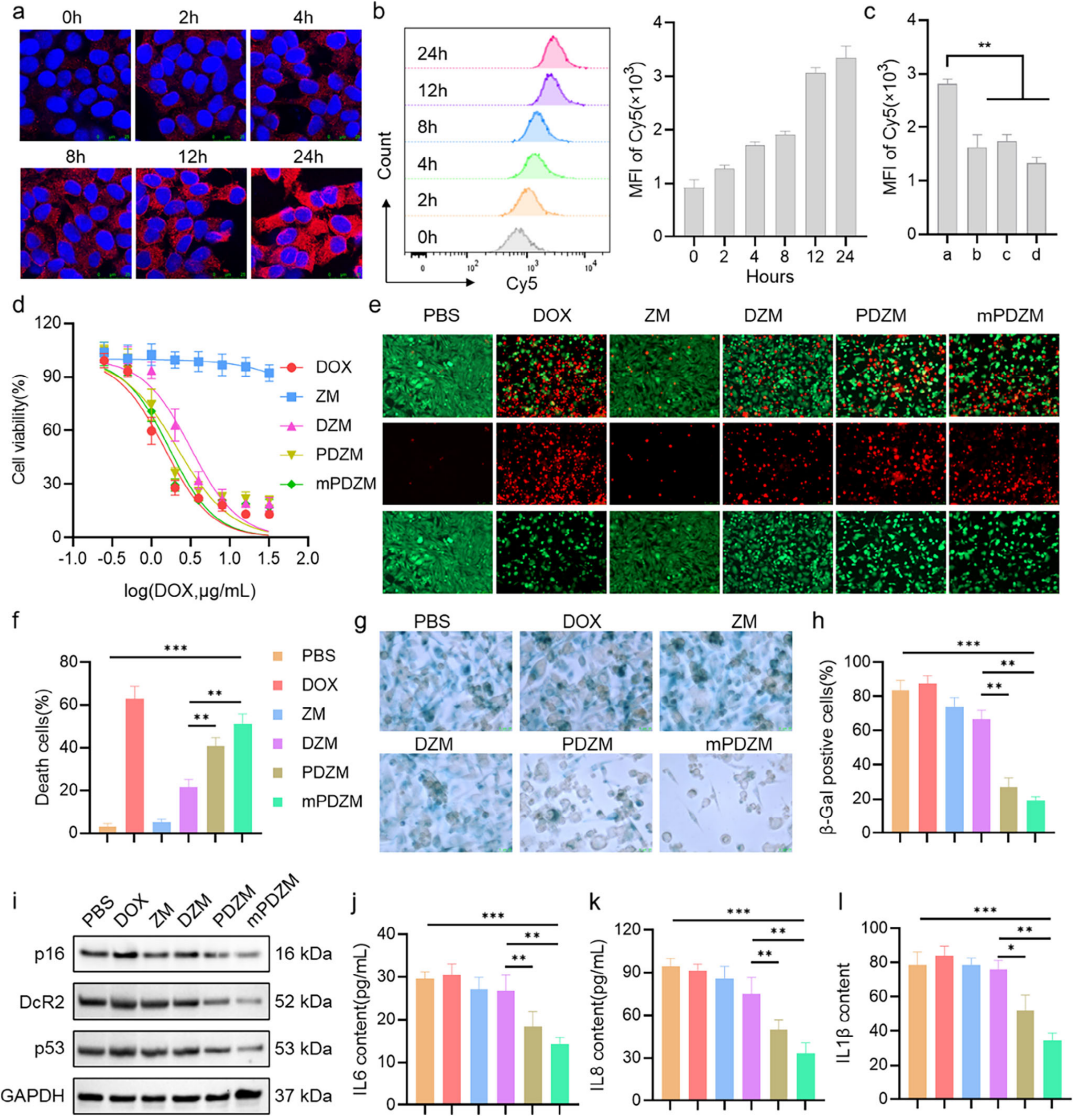
Cellular uptake, cytotoxicity, and therapeutic effects of mPDZM in vitro. a) CLSM images showing intracellular uptake of mPDZM in Hepa1‐6 cells at different time points. Cell nuclei were stained with DAPI (blue), and Cy5 fluorescence is shown in red. b) Flow cytometric analysis and quantitative evaluation of Cy5 fluorescence intensity in Hepa1‐6 cells after incubation with mPDZM for different times. c) Cy5 fluorescence intensity in mPDZM‐treated cells with or without preincubation of sodium azide (10 mm), chlorpromazine (10 µg mL^−1^), or dynasore (80 µm) for 30 min. Note: a: mPDZM, b: mPDZM+ sodium azide, c: mPDZM+ chlorpromazine, d: mPDZM+ dynasore. d) Cell viability of Hepa1‐6 cells treated with free DOX, ZM, DZM, PDZM, or mPDZM, measured by the MTT assay. e) Calcein‐AM/PI staining images of Hepa1‐6 cells treated as indicated. Live cells are shown in green, and dead cells in red. f) Quantification of cell death rate based on Calcein‐AM/PI staining images. g) SA‐β‐gal staining images of Hepa1‐6 cells with indicated treatments. h) Quantification of SA‐β‐gal positive cells from SA‐β‐gal staining images. i) Western blot analysis of senescence‐related proteins including p16, DcR2, and p53, in Hepa1‐6 cells after different treatments. GAPDH was used as a control. ELISA assays measuring the contents of j) IL‐6, k) IL‐8, and l) IL1β released into the supernatants of cell cultures after different treatments. Data are presented as mean ± SD (n = 3). Statistical analysis was performed using one‐way ANOVA + Tukey's test, *p < 0.05, **p < 0.01, ***p < 0.001.

To investigate the cellular internalization behavior, Cy5‐labeled mPDZM nanoparticles were incubated with Hepa1‐6 cells for the indicated time points. A confocal laser scanning microscopy (CLSM) image revealed that a gradual increase in red fluorescence intensity was observed, indicating efficient and time‐dependent internalization of the nanoparticles by Hepa1‐6 cells (**Figure** **3**a). Flow cytometric analysis further confirmed these findings (Figure 3b). Notably, the cellular uptake of mPDZM was markedly reduced upon blockade of energy‐dependent (sodium azide), clathrin‐mediated (chlorpromazine), and dynamin‐dependent (dynasore) endocytic pathways (Figure 3c). These results indicate that the internalization of mPDZM primarily relies on active, clathrin‐ and dynamin‐mediated endocytosis.

The cytotoxicity of various formulations was evaluated in Hepa1‐6 cells using a MTT assay. As shown in Figure 3d, ZM exhibited negligible cytotoxicity, with only ≈10% reduction in cell viability observed even at the highest tested concentration, indicating good biocompatibility of the carrier. In contrast, DZM, PDZM, and mPDZM induced a marked decrease in cell viability. The IC_50_ values of DOX delivered from DZM, PDZM, and mPDZM were determined to be 3.20 ± 0.22, 2.13 ± 0.22, and 1.68 ± 0.06 µg mL^−1^, respectively. Compared with DZM, the IC_50_ was decreased both in PDZM and mPDZM, demonstrating that the stepwise functionalization, especially the incorporation of PL, enhanced the cytotoxicity of DOX. To further validate the cytotoxic effects, Calcein‐AM/PI live/dead staining was performed. As shown in Figure 3e f, mPDZM treatment resulted in a substantial increase in dead cells, significantly exceeding those observed in the DZM and PDZM groups. In addition, mPDZM treatment led to a significantly increased proportion of apoptotic cells, as determined by flow cytometric analysis (Figure S13, Supporting Information). The apoptosis rate in the mPDZM group reached 42.67 ± 4.89%, which was notably higher than that of DZM (20.86 ± 0.42%) and PDZM (34.80 ± 0.78%), though still lower than that of free DOX (62.60 ± 10.98%). Western blot analysis revealed that mPDZM treatment markedly increased the expression of cleaved caspase‐3 (Figure S13, Supporting Information). Pretreatment with the apoptosis inhibitor z‐VAD‐fmk resulted in a significant attenuation of the mPDZM‐induced decrease in cell viability (Figure S14, Supporting Information). These results further confirm the enhanced pro‐apoptotic capability of mPDZM.

To assess the oxidative stress‐triggering potential of mPDZM in vitro, intracellular ROS levels were measured using the DCFH‐DA probe (Figure S15, Supporting Information). A significant increase in ROS level was observed in cells treated with mPDZM. Compared to DZM, mPDZM treatment induced approximately a two‐fold elevation in intracellular ROS levels. Consistently, the intracellular GSH level and SOD activity were significantly reduced in mPDZM‐treated cells. Compared with DZM, the GSH level and SOD activity were markedly lower, supporting the enhanced oxidative stress induced by mPDZM (Figure S15, Supporting Information). Importantly, pretreatment with NAC or GSH significantly alleviated the cytotoxicity induced by mPDZM (Figure S14, Supporting Information). Collectively, these results highlight oxidative stress as a key mechanism underlying the enhanced therapeutic efficacy of mPDZM.

Next, mPDZM was applied to senescent Hepa1‐6 cells to evaluate its senolytic effect. As expected, the proportion of SA‐β‐gal‐positive cells decreased to 19.13 ± 2.17% in mPDZM‐treated cells, which was 4.37‐fold lower than that observed with PBS and 3.61‐fold lower than that with DZM (Figure 3g,h). Interestingly, DZM treatment alone also resulted in a reduction of SA‐β‐gal‐positive cells in DOX‐induced senescent Hepa1‐6 cells. This may be attributed to the ZM component's potential to modulate the senescence microenvironment or promote partial clearance, as evidenced by the reduction in SA‐β‐gal‐positive cells observed in ZM‐treated senescent Hepa1‐6 cells. Importantly, the mPDZM treatment resulted in substantially more efficient clearance of senescent cells than ABT263, Dasatinib + Quercetin, or free PL. (Figure S17, Supporting Information). Western blot analysis further confirmed that the expression levels of p16, DcR2, and p53 were markedly reduced in both PDZM‐ and mPDZM‐treated cells (Figure 3i). Importantly, mPDZM treatment resulted in a pronounced elevation of cleaved‐caspase‐3, demonstrating its ability to trigger apoptosis in senescent cells (Figure S17, Supporting Information). Notably, pretreatment with the pan‐caspase inhibitor z‐VAD‐fmk significantly mitigated the senolytic activity of mPDZM (Figure S17, Supporting Information). By analyzing the SASP profiles (Figure 3j–l), we found that the secretion levels of IL‐6, IL‐8, and IL1β were markedly decreased in mPDZM‐treated cells. Compared to DZM, both PDZM and mPDZM led to reduced secretion of these pro‐inflammatory cytokines. In addition, treatment with mPDZM significantly inhibited NF‐κB activation, which serves as a key modulator of SASP secretion,^[^ ^51^ ^]^ as reflected by a pronounced reduction in phosphorylated p65 (p‐p65) levels (Figure S18, Supporting Information). These results indicate that mPDZM effectively attenuates the SASP response, potentially limiting chronic inflammation and tumor‐promoting signaling associated with senescent cells.

### 2.5 Mechanistic and Immunological Insights

**Figure 4:**
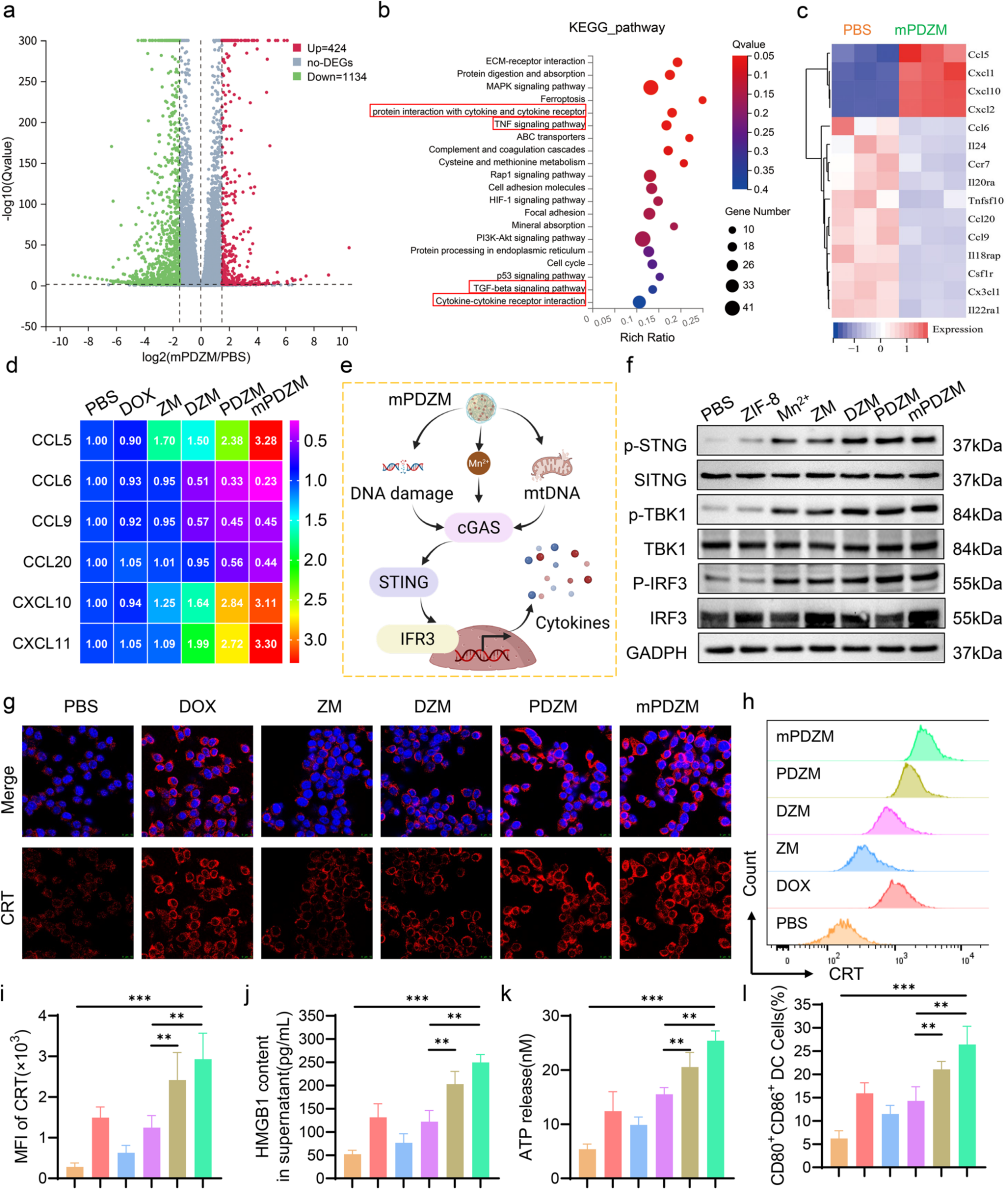
Mechanistic and Immunological Insights of mPDZM. a) Volcano plot of differentially expressed genes (DEGs) between mPDZM‐treated and PBS‐treated cells (|log_2_FC| > 1.5, p < 0.05). b) KEGG pathway enrichment analysis of DEGs. c) Heatmap showing the expression of representative cytokine‐related genes in mPDZM‐ and PBS‐treated cells. d) RT‐qPCR analysis of cytokines in Hepa1‐6 cells with the indicated treatment. e) Schematic illustration of mPDZM‐triggered STING signaling pathway activation mediated by DNA damage, Mn^2+^release, and mitochondrial DNA leakage. f) Western blot analysis of STING, p‐STING, TBK1, p‐TBK1, IRF3, and p‐IRF3 expression in Hepa1‐6 cells with the indicated treatment for 8 h. GAPDH served as a loading control. g) CLSM images showing the expression of CRT in Hepa1‐6 cells with different treatments; nuclei were stained with DAPI. h,i) Flow cytometric analysis and quantification of CRT expression in Hepa1‐6 cells. j) ELISA assays measuring the secretion of HMGB1. k) ATP content determined using a commercial assay kit. l) The percentage of matured DC (CD80^+^CD86^+^) in co‐cultured bone marrow‐derived dendritic cells (BMDCs)and Hepa1‐6 cells pretreated with PBS, DOX, ZM, DZM, PDZM, or mPDZM. Data are presented as mean ± SD (n = 3). Statistical analysis was performed using one‐way ANOVA with Tukey's post hoc test, *p < 0.05, **p < 0.01, ***p < 0.001.

To comprehensively elucidate molecular alterations induced by mPDZM, transcriptomic analysis was conducted. Upon mPDZM treatment, a total of 1558 differentially expressed genes (DEGs) were identified, including 424 upregulated and 1134 downregulated genes, based on a threshold of |log_2_(fold change) | > 1.5 and an adjusted p‐value < 0.05. The DEGs were visualized by a volcano plot and hierarchical clustering heatmap, revealing distinct transcriptional profiles between mPDZM‐treated and control cells (**Figure** **4**a). Furthermore, GO enrichment analysis (Figure S19, Supporting Information) revealed that biological processes of the DEGs were significantly associated with angiogenesis, extracellular matrix organization, cell adhesion, and response to bacteria, as well as apoptosis‐related terms such as positive regulation of apoptotic process and negative regulation of cell proliferation. Immune‐related pathways, including cellular response to tumor necrosis factor and protection from natural killer cell‐mediated cytotoxicity, were also enriched. Molecular function enrichment of DEGs was observed in ATP‐dependent microtubule motor activity, protein and protease binding, and endopeptidase inhibitor activity, suggesting that mPDZM modulates intracellular transport, cytoskeletal organization, and protease activity. Cellular component analysis indicated that DEGs were predominantly associated with the extracellular region, extracellular matrix, cell surface, spindle apparatus, and microtubules, implying that mPDZM may influence both extracellular interactions and intracellular structures related to cell division and migration. KEGG pathway enrichment analysis (Figure 4b). indicated that DEGs were significantly enriched in pathways related to cytokine–cytokine receptor interaction, TNF signaling, and TGF‐beta signaling, indicating that mPDZM modulates key immune and inflammatory signaling cascades. In addition, enrichment in ECM–receptor interaction, focal adhesion, and cell adhesion molecules suggests potential effects on tumor–stroma interactions and cell–matrix communication. Several pathways associated with cell stress and proliferation, such as the p53 signaling pathway, cell cycle, and MAPK signaling pathway, were also enriched, highlighting the multifaceted mechanisms through which mPDZM may exert its antitumor effects. Moreover, GSEA analysis revealed significant enrichment of multiple pathways that converge upstream of or functionally intersect with STING signaling, including the NOD‐like receptor signaling pathway, the RIG‐I‐like receptor signaling pathway, and the Cytosolic DNA Sensing Pathway (Figure S19, Supporting Information).

Given the enrichment of immune‐related pathways, a subset of DEGs associated with immune modulation was visualized by heatmap (Figure 4c). Notably, several upregulated cytokines, including CXCL10 and CXCL11, are known to enhance antitumor immunity by promoting immune cell recruitment and apoptosis. In contrast, the upregulation of CCL5 and downregulation of CCL6, CCL9, and CCL20 suggest that mPDZM may also activate pathways associated with immune suppression or tumor‐associated inflammation. RT‐qPCR analysis (Figure 4d) confirmed the transcriptomic findings, showing upregulated mRNA expression of CCL5, CXCL10, and CXCL11, and downregulated expression of CCL6, CCL9, and CCL20 in mPDZM‐treated cells. Furthermore, ELISA analysis revealed that the secretion of CXCL10,^[^ ^52^, ^53^ ^]^ a chemokine known to recruit CD8⁺ T cells, was significantly increased in mPDZM‐treated cells, which showed the highest level among all treatment groups (Figure S20, Supporting Information). These findings suggest that mPDZM may promote T cell‐mediated antitumor immunity by enhancing the release of cytokines such as CXCL10.

Emerging evidence suggests that the STING signaling pathway plays a central role in regulating innate immune responses by promoting the production of immune‐stimulatory cytokines.^[^ ^54^, ^55^ ^]^ Oxidate stress inducers and manganese are established activators of the STING pathway.^[^ ^54^, ^55^ ^]^ Thus, we hypothesized that the cytokine profiles induced by mPFZM are potentially linked to the activation of the STING signaling pathway (Figure 4e). Western blot analysis (Figure 4f) showed that mPDZM treatment led to a pronounced increase in the levels of phosphorylated STING (p‐STING), TBK1 (p‐TBK1), and IRF3 (p‐IRF3) proteins. In contrast, treatment with DOX or ZM led to only a slight increase in the levels of p‐STING, p‐TBK1, and p‐IRF3. In DZM‐treated cells, a modest yet noticeable increase in the phosphorylation of these proteins was observed. However, the phosphorylation levels remained substantially lower than those in the PDZM and mPDZM‐treated cells. The strong activation of the STING signaling pathway observed in mPDZM‐treated cells is likely attributed to the synergistic contribution of Mn^2+^ release, oxidative stress, and DNA damage. Moreover, our results also showed that treatment with mPDZM markedly activated the STING pathway in DCs, as evidenced by a pronounced increase in phosphorylated STING (p‐STING), p‐TBK1, and p‐IRF3 levels (Figure S21, Supporting Information).

Considering that oxidative stress and DNA damage are also key triggers of ICD to release DAMPs and antitumor cytokines,^[^ ^16^, ^56^ ^]^ we next investigated whether mPDZM treatment could induce ICD‐associated hallmarks in Hepa1‐6 cells. CLSM images revealed that a significant elevation in CRT fluorescence signal was observed in mPDZM‐treated cells compared to PBS (Figure 4g). In contrast, ZM treatment induced minimal CRT exposure. DZM treatment resulted in a modest increase in CRT signals, which remained lower than that observed in the PDZM group. Flow cytometric analysis revealed that the mean fluorescence intensity of CRT in mPDZM‐treated cells was approximately threefold higher than that observed in DZM‐treated cells (Figure 4h,i). Moreover, mPDZM treatment led to an increase in HMGB1 release into the supernatant (Figure 4j). The ATP level in the supernatant was also elevated following mPDZM treatment (Figure 4k), further supporting the induction of ICD.

To validate the ICD‐induced effects on DC maturation, Hepa1‐6 cells pretreated with mPDZM or other formulations were co‐incubated with bone marrow‐derived dendritic cells (BMDCs) at a ratio of 2:1. Flow cytometric analysis revealed that mPDZM‐pretreated Hepa1‐6 cells significantly increased the percentage of CD80⁺CD86⁺ BMDCs to 26.40 ± 3.93%, which was 4.25‐, 2.30‐, and 1.85‐fold higher than that observed in the PBS‐, ZM‐, and DZM‐treated groups, respectively (Figure 4l; Figure S22, Supporting Information). ELISA assay indicated that mPDZM treatment markedly increased IL‐12p70 and decreased IL‐10 (Figure S23, Supporting Information). Taken together, mPDZM effectively enhances antitumor immunity by remodeling cytokine profiles, activating the cGAS‐STING pathway, and inducing ICD.

To determine whether the cytokine and DAMP up‐regulation induced by mPDZM indeed leads to tumor‐antigen‐specific T‐cell activation, we performed an antigen‐specific cross‐presentation assay^[^ ^57^ ^]^ (Figure S24, Supporting Information). Specifically, Hepa1‐6 cells were treated with mPDZM, and the resulting supernatants were used to stimulate OVA_257‐264_ pulsed BMDCs. BMDCs were subsequently co‐cultured with OT‐I CD8^+^ T cells, which recognize the SIINFEKL peptide presented by H‐2Kb. Our results showed that mPDZM‐treated tumor supernatants significantly enhanced H‐2KbSIINFEKL complex expression on BMDCs, indicating improved antigen cross‐presentation. In the co‐culture system, we further observed a marked increase in CD8^+^ T‐cell proliferation, accompanied by a substantial elevation in IFN‐γ production. These findings demonstrate that the immunogenic molecules induced by mPDZM functionally promote antigen‐specific CD8^+^ T‐cell expansion.

### 2.6 In Vivo *b*iodistribution, magnetic resonance visibility, and biosafety

**Figure 5:**
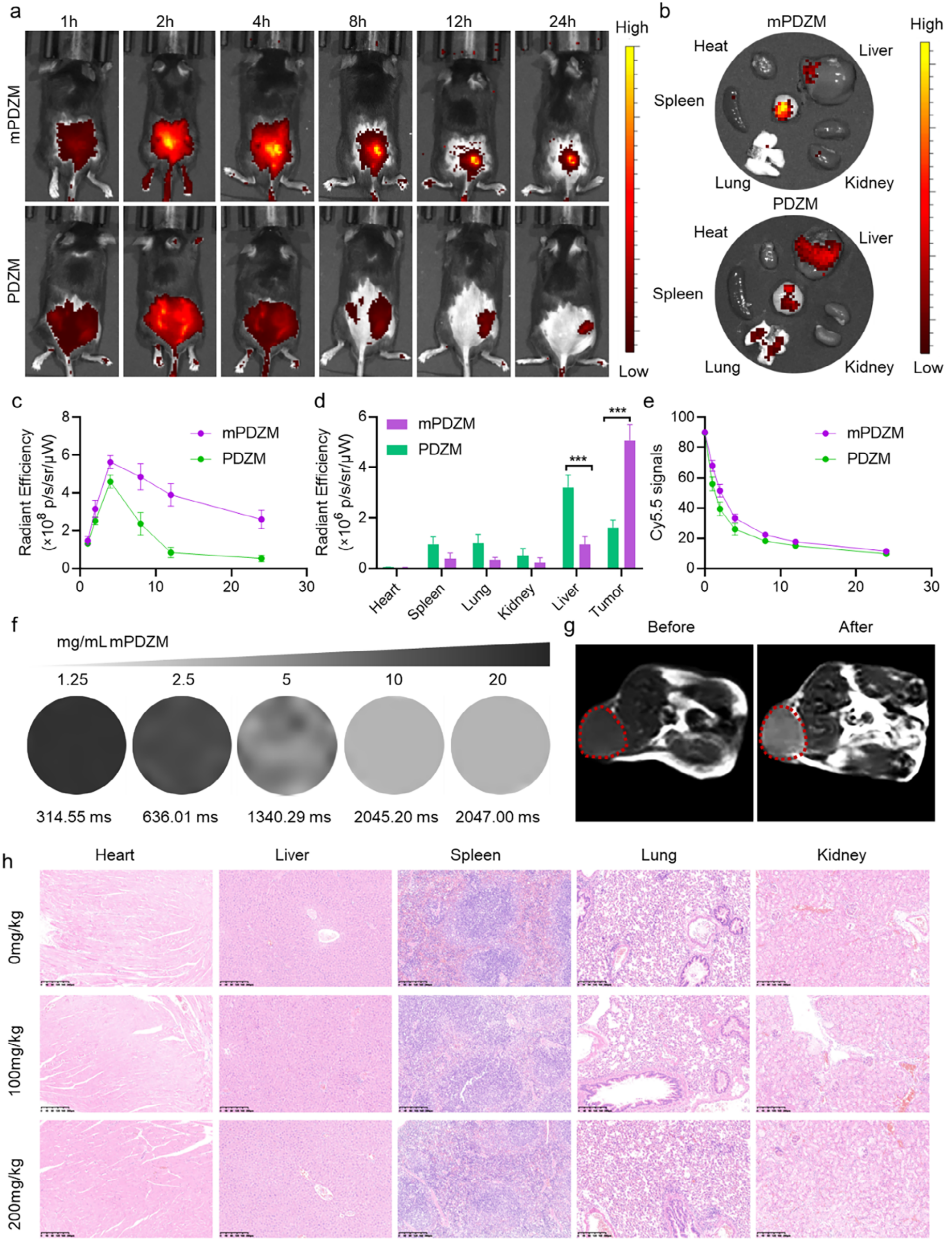
In vivo biodistribution, imaging performance, biosafety assessment. a) In vivo fluorescence imaging of Hepa1‐6 tumor‐bearing mice at different time points post‐intravenous injection of mPDZM or PDZM. b) Ex vivo fluorescence images of major organs and tumors at 24 h post‐injection. c) Quantitative fluorescence intensity analysis of tumors over time. d) Fluorescence quantification of organs and tumors at 24 h post‐injection. e) Blood circulation profiles of mPDZM or PDZM. f) MRI images with increasing mPDZM concentrations. g) In vivo MR images of tumor sites before and after mPDZM injection. h) Representative H&E staining sections of major organs (heart, liver, spleen, lung, kidney) from healthy mice treated PBS or mPDZM at low and high doses. Data are presented as mean ± SD (n = 3). Statistical analysis was performed using one‐way ANOVA + Tukey's test, *p < 0.05, **p < 0.01, ***p < 0.001.

The biodistribution of mPDZM was further investigated in vivo, with PDZM used as a control. Briefly, both PDZM and mPDZM were labeled with Cy5 and intravenously injected into tumor‐bearing mice. Using the IVIS Spectrum imaging system, tumor accumulation was monitored by tracking the Cy5 fluorescence signals at 0, 1, 2, 4, 8, 12, and 24 h post‐injection. As shown in **Figure** **5**a,c both PDZM and mPDZM effectively accumulated in the tumor tissue, as evidenced by the increased Cy5 fluorescence intensity. Notably, mPDZM exhibited a stronger and more prolonged tumor‐targeting signal compared to PDZM, suggesting enhanced tumor accumulation and retention. At 24 h post‐injection, the mice were sacrificed, and the tumor tissues, along with major organs (heart, liver, spleen, lungs, and kidneys), were harvested for *ex vivo* fluorescence imaging. Cy5 signals were retained in both PDZM‐ and mPDZM‐treated tumors (Figure 5b,d), likely due to the enhanced permeability and retention effect. Notably, mPDZM‐treated tumors exhibited significantly stronger fluorescence signals, indicating enhanced tumor‐homing capability conferred by the cell membrane coating. Moreover, Cy5 fluorescence in the liver was markedly reduced in mPDZM‐treated mice, whereas higher hepatic fluorescence was observed in PDZM‐treated mice. Blood circulation profiles revealed that mPDZM exhibited significantly prolonged retention in the bloodstream. The distribution half‐life of 1.62 h and an elimination half‐life of 20.75 h for mPDZM, longer than those of PDZM (0.99 h and 16.54 h). These results suggest that CM coating can effectively prolong the circulation time of nanoparticles by reducing their clearance rate from the body (Figure 5e).

Given that mPDZM contains MnO_x_ capable of releasing Mn^2+^ in the acidic tumor microenvironment, we further evaluated its T_1_‐weighted magnetic resonance (MR) imaging performance to validate its tumor‐targeted imaging capability.^[^ ^38^, ^58^ ^]^ Ex vivo MR imaging demonstrated a concentration‐dependent T_1_ signal enhancement of mPDZM (Figure 5f), confirming the Mn^2+^‐mediated MR contrast capability. Subsequently, in vivo T_1_‐weighted MRI was performed post‐intravenous administration of mPDZM. As shown in Figure 5g, an increase in MR signal intensity was observed at the tumor site, indicating effective accumulation and Mn^2+^ release. These results confirmed that mPDZM enabled efficient tumor‐specific MRI visualization with optimal imaging performance, consistent with its Mn^2+^ release behavior and in vivo biodistribution profile.

To assess the in vivo biosafety of mPDZM, healthy C57BL/6J mice were intravenously administered with mPDZM (100 and 200 mg kg^−1^). No significant changes in behaviors were observed during the 7‐day observation period. Hematological and serum biochemical parameters (WBC, RBC, PLT, ALT, CR) remained within normal ranges compared to 0 mg kg^−1^‐treated controls (Figure S25, Supporting Information). Furthermore, H&E staining of major organs (heart, liver, spleen, lung, and kidney) revealed no observable histopathological abnormalities (Figure 5h). These data collectively demonstrate that mPDZM possesses favorable biocompatibility and safety in vivo.

### 2.7 Antitumor Effects In Vivo

**Figure 6:**
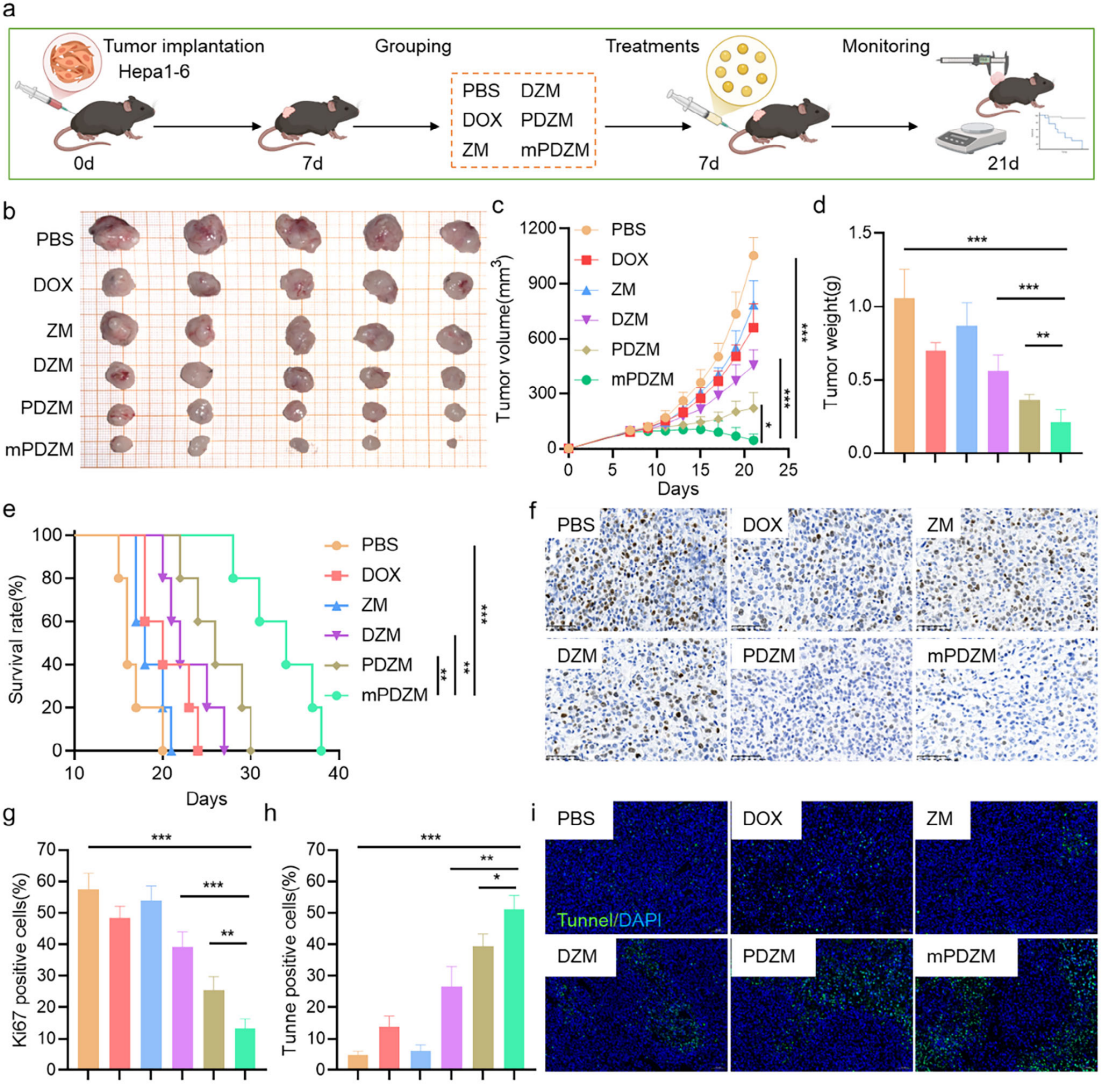
In vivo antitumor efficacy of mPDZM in Hepa1‐6 tumor‐bearing mice. a) Schematic illustration of the in vivo experimental design. b) Representative images of excised tumors from mice after 21 days of treatment with PBS, DOX, ZM, DZM, PDZM, or mPDZM. c) Tumor growth curves during treatment. d) Tumor weights at the end of the experiment. e) Kaplan–Meier survival curves of mice in different treatment groups (n = 5 per group). f) Immunohistochemical staining of Ki‐67 in tumor tissues from each group. g) Quantification of Ki‐67 positive cells from immunohistochemical images. h) Quantification of Tunnel positive cells from immunohistochemical images. i) Tunnel staining (green) in tumor tissues from each group; nuclei were counterstained with DAPI (blue). Data are presented as mean ± SD. Statistical analysis was performed using one‐way ANOVA + Tukey's test, *p < 0.05, **p < 0.01, ***p < 0.001.

To test the anti‐tumor effects in vivo, a murine subcutaneous tumor model was established by injecting Hepa1‐6 cells into the right flank of mice (**Figure** **6**a). After tumors reached a volume of ≈100 mm^3^, mice were randomly assigned to different treatment groups. Subsequently, nanoparticles were intravenously injected. Tumor growth was monitored by measuring tumor volume every other day. At the endpoint, tumor tissues were excised for further analysis and survival was evaluated. As shown in Figure 6b, the tumor masses in mice treated with mPDZM were significantly smaller than those in the other treatment groups, indicating a superior antitumor efficacy. Notably, the tumor growth rate in the mPDZM group was markedly slower compared to the other groups throughout the treatment period. At 14 days post‐treatment, the tumor volume in the mPDZM group was the lowest among all groups, being ≈4.59 times smaller than that in the PBS group. Compared to PBS, treatment with DOX alone resulted in a modest tumor inhibition rate of 27.36%, while DZM and PDZM achieved tumor inhibition rates of 45.54% and 61.16%, respectively. Notably, mPDZM exhibited the highest antitumor efficacy, with a tumor inhibition rate of 78.22%. In line with tumor volume results, the final tumor weights in the mPDZM‐treated group were significantly lower than those in the other groups (Figure 6d). This consistent trend between tumor volume and weight further validates the potent antitumor activity of mPDZM. Importantly, our results demonstrated that these nanoplatforms did not induce overt myelosuppression, hepatic injury, or renal dysfunction. Hematological and serum biochemical parameters remained within normal ranges across all groups (Figure S26, Supporting Information). To further evaluate the therapeutic benefit of mPDZM, the survival of tumor‐bearing mice was monitored. Kaplan–Meier survival analysis (Figure 6e) revealed that mice treated with mPDZM exhibited significantly prolonged survival compared to other treatment groups. Statistical analysis revealed that the survival rate in the mPDZM group was significantly higher than that in the PDZM, DZM, and PBS groups, indicating its superior therapeutic efficacy.

To comprehensively evaluate the therapeutic effects and underlying mechanisms of different formulations, histological analyses were performed. H&E staining revealed that tumor sections from the PBS and ZM groups exhibited dense cellularity and intact morphology, indicating limited therapeutic effect. The tumor in the DOX, DZM, and PDZM groups showed partial tumor cell disorganization and moderate necrotic areas. In contrast, the tumor from the mPDZM‐treated group displayed extensive tissue damage, characterized by disrupted cellular architecture and widespread necrosis, suggesting a strong therapeutic response (Figure S27, Supporting Information). Ki67 immunofluorescence staining further revealed differences in proliferative activity (Figure 6f). The Ki67‐positive rates in the PBS and DOX groups were 57.53 ± 5.18% and 48.50 ± 3.59%, respectively. These values decreased to 39.25 ± 4.82% in the DZM group and 21.54 ± 4.03% in the PDZM group, indicating that nanoparticle delivery and PL incorporation enhanced the antiproliferative effect of DOX. Notably, the mPDZM group showed the lowest Ki67 expression (13.26 ± 3.11%), suggesting superior inhibition of tumor proliferation (Figure 6g). TUNEL staining was employed to assess apoptosis within the tumors (Figure 6h,i). Compared with the PBS group (4.70 ± 1.35%), the percentage of TUNEL‐positive cells increased to 13.71 ± 3.55% in the DOX group, 26.62 ± 6.33% in the DZM group, and 42.14 ± 4.29% in the PDZM group. The mPDZM group demonstrated the highest percentage of apoptotic cells, with 51.02 ± 4.56%, which was 3.7‐fold higher than DOX, 1.9‐fold higher than DZM, and 1.2‐fold higher than PDZM. These results indicated that the cancer cell membrane coating significantly enhanced the pro‐apoptotic effect of the nanoplatform.

Building on the promising antitumor efficacy, we further investigated oxidative stress, a key mediator of tumor inhibition through oxidative damage and activation of apoptotic signaling pathways.^[^ ^59^, ^60^ ^]^ Strikingly, the mPDZM group exhibited the highest ROS level, 5.38‐fold compared to PBS, which was approximately three times higher than DOX, 1.8 times higher than DZM, and 1.2 times higher than PDZM (Figure S28, Supporting Information). These results suggest that the enhanced therapeutic efficacy of mPDZM is closely associated with excessive ROS generation, increased apoptosis, and suppressed tumor cell proliferation. In addition, tumor hypoxia was evaluated by pimonidazole staining and HIF‐1α expression (Figure S29, Supporting Information). Our results revealed that MnOx‐deposited nanoparticles, including ZM, DZM, PDZM, and mPDZM, effectively alleviated tumor hypoxia, as evidenced by the markedly reduced pimonidazole fluorescence. Correspondingly, HIF‐1α expression was significantly downregulated in these groups, further confirming the oxygen‐generating and hypoxia‐relieving capability of MnOx‐deposited nanoparticles. Overall, mPDZM exerts superior antitumor effects by synergistically modulating proliferation, apoptosis, oxidative stress, and hypoxia.

### 2.8 Senolytic Efficacy of mPDZM In Vivo

**Figure 7:**
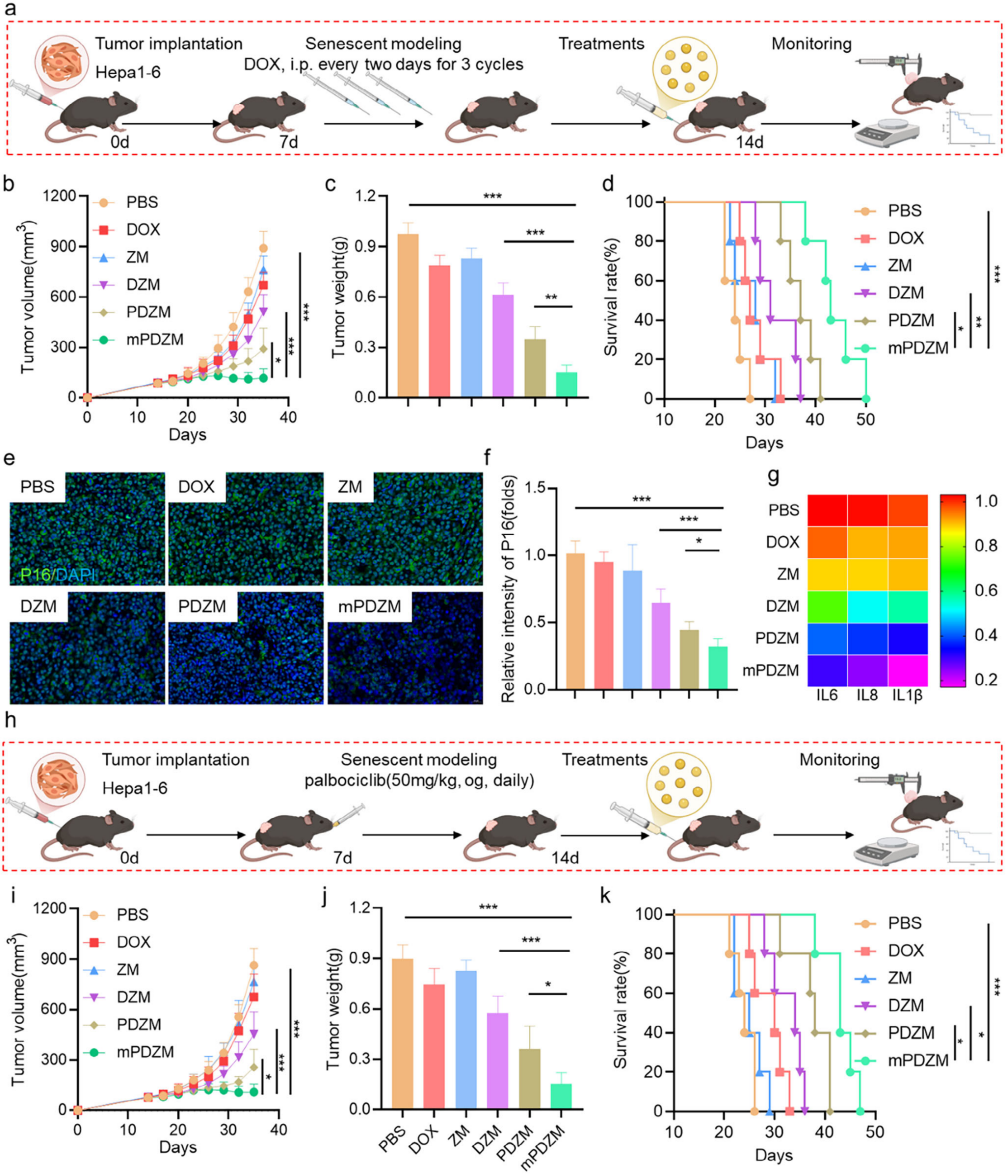
Senolytic efficacy of mPDZM in vivo. a) Schematic illustration of the in vivo experimental design for establishing a DOX‐induced tumor senescence model. Hepa1‐6 tumor‐bearing mice were intraperitoneally injected with doxorubicin (DOX, 1 mg kg^−1^, once every 1 day for a total of three doses) to induce tumor senescence. The mice were treated with PBS, DOX, ZM, DZM, PDZM, mPDZM via tail vein injection. b) Tumor growth was monitored, and growth curves were plotted. c) Tumor weights at the end of the treatment period. d) Kaplan–Meier survival curves for each treatment group. e) Representative immunofluorescence images of p16 (green) expression in tumor sections from different groups, with nuclei counterstained using DAPI (blue). f) Quantification of p16 fluorescence intensity. g) Relative levels of SASP factors IL‐6, IL‐8, and IL1β in tumor tissues from different treatment groups, measured by ELISA and normalized to the PBS group. h) Schematic illustration of the in vivo experimental design for establishing a palbociclib‐induced tumor senescence model. Hepa1‐6 tumor‐bearing mice were orally administered palbociclib (50 mg kg^−1^, once daily) for 7 consecutive days to induce tumor senescence. i) Tumor growth curves during treatment in the palbociclib‐induced model. j) Tumor weights at the end of the treatment period. k) Kaplan–Meier survival curves for each treatment group in the palbociclib‐induced model. Data are presented as mean ± SD. Statistical analysis was performed using one‐way ANOVA + Tukey's test, *p < 0.05, **p < 0.01, ***p < 0.001.

To evaluate the in vivo senolytic efficacy of mPDZM, a tumor senescence model was established.^[^ ^61^ ^]^ Briefly, C57BL/6 mice bearing subcutaneous Hepa1‐6 tumors were intraperitoneally administered with DOX (1 mg kg^−1^) every other day for a total of three injections over the course of one week to induce tumor cell senescence. Once tumor volumes reached ≈80–100 mm^3^, mice were randomly assigned to the indicated treatment groups. Tumor growth was regularly monitored, and survival benefits were assessed. At the study endpoint, tumors were harvested for histological and molecular analyses (**Figure** **7**a). As shown in Figure 7b, the tumors in the PBS, DOX, and ZM groups grew rapidly, and moderate inhibition was observed in the DZM group. The tumor growth rate in the PDZM group was significantly lower than that in the DZM group, but still higher than that observed in the mPDZM group. Obviously, the tumor volume in the mPDZM group was the lowest among all groups, indicating the potent senolytic and antitumor efficacy of mPDZM in a DOX‐induced tumor senescence model. At 21 days post‐administration, tumors were excised and weighed to further assess the tumor inhibition of mPDZM. As expected, the tumor inhibition rate in the mPDZM group reached 83.99%, which was markedly higher than that observed in the PDZM, DZM, ZM, and DOX groups (Figure 7b). Kaplan–Meier survival analysis (Figure 7c) indicated that all mice in the PBS and ZM group, all individuals reached the endpoint before day 28. The DOX groups showed only marginal improvements, with all mice dying before day 33. In contrast, mice treated with DZM exhibited moderately prolonged survival. A more pronounced survival benefit was observed in the PDZM group, where mice survived up to 41 days. Notably, treatment with mPDZM significantly extended overall survival, with mice living as long as 50 days post‐treatment. Immunofluorescence analysis (Figure 7e,f) revealed that minimal changes in p16 expression were observed in the DOX and ZM groups compared to PBS. In contrast, the DZM group exhibited a moderate reduction in p16 levels, possibly due to the clearance of early‐stage senescent cells that are sensitive to stress‐induced apoptosis even without a dedicated senolytic agent. PDZM and mPDZM, both loaded with the senolytic agent PL, significantly reduced p16 expression, highlighting their superior ability to eliminate senescent cells. Furthermore, in vivo analysis of the SASP profile revealed significant suppression of senescence‐associated cytokines following mPDZM treatment (Figure 7g). The mRNA expression levels of IL‐6, IL‐8, and IL1β were markedly reduced in the mPDZM group, indicating effective inhibition of the pro‐inflammatory secretory phenotype.

To further validate our findings, another senescent tumor model was established using palbociclib, a selective CDK4/6 inhibitor known to induce senescence.^[^ ^62^ ^]^ Briefly, on day 7 post‐tumor implantation, palbociclib was administered orally daily for 7 consecutive days (Figure 7h). Subsequently, the animals were randomly assigned to the indicated treatment groups. As expected, DZM, PDZM, and mPDZM treatments significantly suppressed tumor growth in vivo (Figure 7i). By day 21 post‐treatment, the average tumor volumes in the DZM, PDZM, and mPDZM groups were reduced to 52.42%, 27.34%, and 12.64% of the volume observed in the PBS group, respectively. A similar trend was observed in tumor weight (Figure 7j), further supporting the antitumor efficacy of the treatments. Kaplan–Meier analysis demonstrated that mPDZM treatment significantly prolonged survival in the palbociclib‐induced tumor senescence model (Figure 7k). Collectively, mPDZM exhibited potent in vivo senolytic efficacy in a therapy‐induced tumor senescence model.

### 2.9 Immunomodulatory Effects

**Figure 8:**
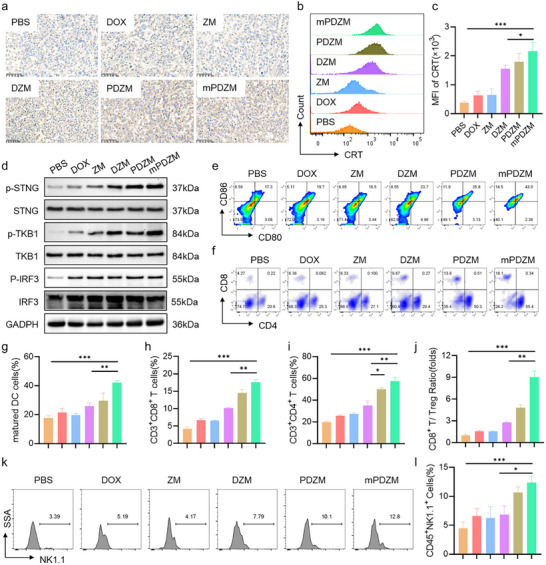
Immunomodulatory effects of mPDZM in vivo. a) Representative immunohistochemical staining of calreticulin (CRT) in tumor tissues from Hepa1‐6 tumor‐bearing mice after the indicated treatments. b) Representative flow cytometry histograms and c) quantitative analysis of CRT expression on tumor cells. d) Western blot analysis of STING signaling pathway‐related proteins, including STING, p‐STING, TBK1, p‐TBK1, IRF3, and p‐IRF3, in tumor tissues. GAPDH served as a loading control. e) Representative flow cytometry plots and g) quantitative analysis of mature DC (CD80^+^CD86^+^) in tumor‐draining lymph nodes (TDLNs). f) Representative flow cytometry plots of tumor‐infiltrating T cells. h) Quantitative analysis of CD8^+^ and i) CD4^+^ T cells in tumors. j) Ratio of CD8⁺ T cells to regulatory T cells (Tregs, CD4⁺CD25⁺Foxp3⁺) in tumors. k) Representative flow cytometry histograms and quantitative analysis of NK1.1⁺ natural killer (NK) cells in tumors.

It is supported that both senescence induction and the elimination of prolonged senescent cells facilitate the antitumor immune response.^[^ ^32^, ^63^ ^]^ Moreover, the capacity of mPDZM to induce ICD and activate the STING signaling pathway may further potentiate the enhanced antitumor response within TME.^[^ ^16^, ^64^ ^]^ Thus, we sought to investigate whether mPDZM could potentiate immune activation within the TME. Immunohistochemical analysis (**Figure** **8**a) indicated that minimal CRT exposure was observed in the PBS, DOX, and ZM groups, indicating limited ICD induction. In contrast, treatment with DZM, PDZM, and mPDZM increased CRT expression, suggesting a robust induction of ICD in vivo. This observation was further supported by flow cytometric analysis (Figure 8b,c), which revealed that mPDZM treatment significantly elevated the MFI of CRT, ≈5.64 times higher than that in the PBS group. Moreover, Western blot analysis (Figure 8d) indicated that mPDZM markedly increased the phosphorylation levels of STING, TBK1, and IRF3 in vivo, without affecting the total protein levels of STING, TBK1, or IRF3. These findings suggest that mPDZM activates the STING signaling pathway, a key mediator of innate immune sensing and type I interferon responses.

Subsequently, flow cytometric analysis revealed that mPDZM effectively enhances DC activation in tumor‐draining lymph nodes, as evidenced by the significant increase of the proportion of CD80^+^CD86^+^ mature DC to 42.1 ± 1.56% (Figure 8e,g). Compared to the PBS group, this proportion was ≈2.4‐fold higher. Indeed, it was also 2.9‐fold higher than that in the ZM group (14.5 ± 0.96%) and 1.4‐fold higher than in the PDZM group (29.67 ± 5.31%), indicating that mPDZM more effectively promotes DC maturation. mPDZM also promoted robust T cell infiltration and activation within the TME. The CD3⁺CD8⁺ T cell infiltration in mPDZM‐treated tumors increased to 17.63 ± 0.72%. In comparison, DZM and PDZM treatments resulted in moderate increases to 10.12 ± 0.23% and 14.50 ± 0.96%, respectively. However, DOX and ZM treatments only led to a slight elevation in CD3⁺CD8⁺ T cell levels (Figure 8f,h). Furthermore, CD3⁺CD4⁺ T cell infiltration was markedly increased in the mPDZM group, with a proportion of 57.5 ± 3.21%. This proportion was ≈1.15‐, 1.64‐, and 2.93‐fold higher than those observed in the PDZM, DZM, and PBS groups, respectively (Figure 8f,i). Functionally, the IFN‐γ⁺CD8⁺ T and GramzB ⁺CD8⁺ T cells, indicative of activated cytotoxic lymphocytes in the mPDZM group, were much higher than ZM, DZM, and PDZM (Figure S30, Supporting Information). Moreover, mPDZM also reduced the proportion of immunosuppressive Tregs, further shifting the TME toward an immune‐activating state. The proportion of Tregs (CD4⁺FOXP3⁺) was markedly reduced in the mPDZM group to 18.47 ± 1.77% (Figure S30, Supporting Information). Consequently, the CD8⁺/Treg ratio surged to 9.07 ± 0.80 in the mPDZM group (Figure 8j), indicating a shift toward a cytotoxic‐dominant immune profile.

Flow cytometric analysis also revealed that the percentage of NK1.1⁺ cells was higher in the mPDZM group compared to the other groups, with a proportion of 13.03 ± 0.21%. Compared to the PBS group, NK1.1⁺ cell infiltration in the mPDZM group increased by ≈2.93 times. Elevated NK1.1⁺ cells were also observed in the PDZM groups, with rates of 10.63 ± 1.01%, but remained lower than that in the mPDZM group (Figure 8k,l). These results suggest that mPDZM more effectively promotes the recruitment and accumulation of NK cells to enhance the innate immune activation. In consideration of the SASP‐mediated immunological context, we further investigated whether mPDZM influenced macrophage dynamics within TME. As shown in Figure S31 (Supporting Information), mPDZM treatment slightly increased the proportion of F4/80⁺ macrophages, possibly due to enhanced local inflammatory responses. Notably, the proportion of M1‐like TAMs increased to 38.60 ± 5.41%, which was ≈2.97‐fold higher than that observed in the PBS group, indicating a favorable shift toward a pro‐inflammatory antitumor macrophage phenotype. mPDZM treatment markedly reduced the proportion of immunosuppressive M2‐like macrophages to 17.87 ± 2.90%, which was much lower than PBS group. These results demonstrate that mPDZM successfully reprograms TAMs toward a pro‐inflammatory phenotype. Our findings highlight the immunotherapeutic potential of mPDZM, which activates both innate and adaptive immune responses.

To comprehensively characterize the impact of mPDZM on the tumor immune landscape, CyTOF analysis was performed (Figures S32–S35, Supporting Information). A total of 24 immune cell clusters were identified by automated clustering and annotated based on canonical lineage markers. t‐SNE visualization revealed a pronounced shift in immune composition following mPDZM treatment compared with PBS controls. Notably, mPDZM markedly expanded several antitumor effector populations, including CD8⁺ cytotoxic T cells (C11), effector/memory T cells (C03, C04, C17), activated NK cells (C23), and activated dendritic cells (C22), while reducing immunosuppressive or tumor‐promoting subsets such as inflammatory monocytes (C05) and M2‐like macrophages (C19). Quantitative analysis confirmed that mPDZM significantly increased effector/memory CD8⁺ T cells (C03, *p* < 0.01), while naive/resting T cells (C10) were markedly decreased (*p* < 0.01). CD4⁺ conventional T cells (C11) and CD8⁺ cytotoxic T cells (C12) also exhibited an increasing trend. Within the dendritic‐cell compartment, mPDZM selectively promoted DC maturation, leading to a significant expansion of mature DCs (C24, *p* < 0.01). Myeloid‐cell remodeling was also evident. mPDZM trended to increase M1‐like macrophages (C19, *p* < 0.01), while reducing tissue macrophages (C08, *p* < 0.05), indicating a shift toward pro‐inflammatory, antigen‐presenting phenotypes. At the molecular level, mPDZM enhanced T‐cell activation, as demonstrated by elevated CD69 and CD27, and significantly increased the co‐stimulatory molecule ICOS. Effector differentiation was further supported by increased TCRβ, and CD127 expression. NK cell activation was corroborated by elevated NK1.1 and CD49b expression. Additionally, mPDZM modulated chemokine receptor profiles, increasing CCR6 and trending upward for CXCR3, suggesting improved recruitment and trafficking of effector lymphocytes. Collectively, these findings demonstrate that mPDZM orchestrates a coordinated remodeling of the tumor immune microenvironment, expanding effector T‐, NK‐ cell populations, promoting myeloid reprogramming, and enhancing lymphocyte activation and trafficking, thereby establishing a highly immunostimulatory tumor milieu favorable for antitumor immunity.

### 2.10 Abscopal Effect and Synergy with Anti‐PD‐L1 Antibody

**Figure 9:**
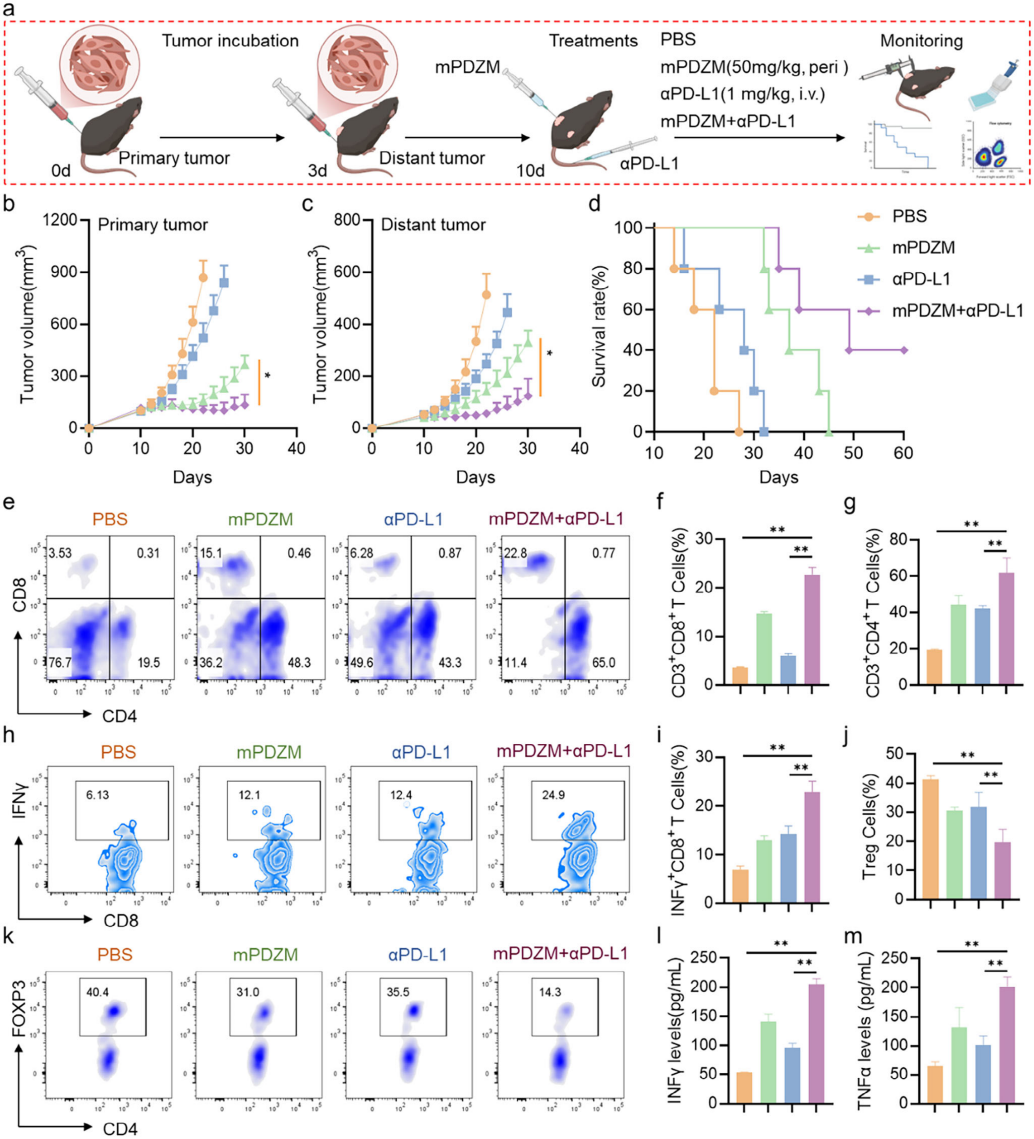
Abscopal effect and synergistic antitumor efficacy of mPDZM combined with anti‐PD‐L1 antibody. a) Schematic illustration of the in vivo experimental design for evaluating the combination of mPDZM and anti‐PD‐L1 (αPD‐L1) antibody in a bilateral tumor‐bearing mouse model. b) Tumor growth curves of primary (treated) tumors in each group. c) Tumor growth curves of distant (untreated) tumors in each group. d) Kaplan–Meier survival curves for mice in each treatment group. e) Representative flow cytometry plots of T cells in distant tumors. f) Quantitative analysis of f) CD8^+^ and g) CD4^+^ T cells in distant tumors. h) Representative flow cytometry plots showing IFNγ^+^CD8^+^ T cells in tumors. i) Quantification of IFNγ^+^CD8^+^ T cells in tumors. j) Quantification and k) representative flow cytometry plots of CD8⁺ T cells in distant tumors. ELISA assays revealing serum levels of l) IFNγ and m) TNFα in each treatment group. Data are presented as mean ± SD. Statistical analysis was performed using one‐way ANOVA + Tukey's test, *p < 0.05, **p < 0.01, ***p < 0.001.

Inspired by the immunomodulatory effects of mPDZM, we next aimed to determine whether it induces an abscopal immune response and synergizes with anti‐PD‐L1 therapy. A bilateral tumor model was first established by subcutaneously injecting Hepa1‐6 cells into the left flank on day 0 (primary tumor) and into the right flank on day 3 (distant tumor). Mice bearing primary tumors of ≈100 mm^3^ and similarly sized distant tumors were randomly assigned to receive treatment with PBS, mPDZM, anti‐PD‐L1, or the combination of mPDZM and anti‐PD‐L1(mPDZM+anti‐PD‐L1). mPDZM was administered via peritumoral injection into the primary tumor at a dose equivalent to one‐third of the intravenous dosage (**Figure** **9**a).

As shown in Figure 9b, peritumoral administration of mPDZM markedly suppressed tumor progression, as evidenced by significantly slower tumor growth compared to the PBS group. Compared to anti‐PD‐L1 monotherapy, the combination with mPDZM and anti‐PD‐L1 resulted in a significant delay in tumor growth. At day 12 post‐treatment, mPDZM monotherapy reduced tumor volume by 39.99% compared to the PBS group. Tumors in the mPDZM + anti‐PD‐L1 group were substantially smaller than those in the anti‐PD‐L1monotherapy group. Although no significant difference was observed at day 14, tumor volume in the mPDZM + anti‐PD‐L1 group was markedly lower than that in the mPDZM group by day 30. These results indicated that the combination of mPDZM and anti‐PD‐L1 provided enhanced and durable antitumor efficacy compared to either monotherapy. Consistent with the primary tumor response, the distant tumors showed a similar reduction in volume (Figure 9c). In the distant tumors, anti‐PD‐L1 or mPDZM monotherapies reduced tumor volumes by 51.94% and 72.25%, respectively. Notably, the combination of mPDZM and anti‐PD‐L1 achieved the most pronounced effect, with a tumor volume reduction of 89.04% at day 12 post‐treatment. Compared to mPDZM monotherapy, the tumor volume in the mPDZM + anti‐PD‐L1 group was markedly lower. Importantly, neither the combination therapy nor the monotherapies caused significant changes in WBC, RBC, PLT, Hb, ALT, ALB, TBIL, or creatinine, indicating the absence of overt hematologic, hepatic, or renal toxicity (Figure S36, Supporting Information). Kaplan–Meier survival analysis (Figure 9d) showed that all mice in the PBS group reached the endpoint by day 27. The anti‐PD‐L1 monotherapy modestly extended survival to day 32. In contrast, mPDZM treatment further prolonged survival to day 45. Notably, the combination of mPDZM and anti‐PD‐L1 elicited the most pronounced therapeutic benefit, with several mice surviving up to 60 days. To further evaluate whether mPDZM exerts inhibitory effects on spontaneous metastasis, we additionally established an orthotopic Hepa1‐6 liver tumor model. Mice were divided into four groups: PBS, anti‐PD‐L1, mPDZM, and mPDZM + anti‐PD‐L1. H&E staining (Figure S37, Supporting Information) revealed that mice receiving mPDZM or anti‐PD‐L1 monotherapy exhibited reduced micro‐metastatic foci. In contrast, the combination treatment (mPDZM + anti‐PD‐L1) resulted in pronounced suppression of spontaneous metastasis, with fewer metastatic lesions in the examined tissues. These results indicate that mPDZM, particularly when combined with anti‐PD‐L1, markedly reduces spontaneous metastatic dissemination. Taken together, these findings indicate that mPDZM synergizes with immune checkpoint blockade to produce a potent and durable antitumor effect in vivo.

To further confirm the abscopal effects, we assessed immune cell infiltration in distal tumor sites that were not directly exposed to mPDZM treatment. As shown in Figure 9e, treatment with PDZM and anti‐PD‐L1 moderately increased intra‐tumoral CD3⁺CD8⁺ T cell levels to 5.96 ± 0.52% and 14.7 ± 0.4%, respectively. Notably, the combination of mPDZM and anti‐PD‐L1 elicited the highest infiltration of CD3⁺CD8⁺ T cells, reaching 22.77 ± 1.45% (Figure 9f). Similarly, the combination of mPDZM and anti‐PD‐L1 resulted in the most pronounced CD3⁺CD4⁺ T cell infiltration, with a proportion of 62.03 ± 8.16% (Figure 9g). Upon treatment with mPDZM or anti‐PD‐L1, the levels increased to 12.97 ± 0.90% and 14.27 ± 1.62%, respectively. The mPDZM + anti‐PD‐L1 group showed the greatest increase in IFN‐γ⁺CD8⁺ T cells, with a proportion of 22.83 ± 2.27% (Figure 9h,i). In addition, the percentage of GrmB⁺CD8⁺ T cells was higher in the mPDZM group compared to the other groups (Figure S38, Supporting Information). Compared to the PBS group, mPDZM or anti‐PD‐L1 treatments reduced Treg levels by ≈1.35‐fold and 1.29‐fold, respectively. Notably, mPDZM treatment resulted in a more pronounced decrease in Treg levels to 19.57 ± 4.57% (Figure 9j,k). Importantly, the combination group exhibited a higher proportion of NK1.1⁺ cells compared to the other groups. (Figure S38, Supporting Information). Indeed, the serum levels of IFN‐γ and TNF‐α in the mPDZM + anti‐PD‐L1 group were higher than those observed in either the mPDZM or anti‐PD‐L1 group (Figure 9l,m), further highlighting a more robust systemic immune response elicited by the combination therapy.

## 3 Conclusion

In this study, we developed a multifunctional nanoplatform that integrates a redox‐responsive ZIF‐8@MnOx with a homologous tumor cell membrane coating, designed to effectively implement a “one‐two punch” strategy against HCC. The co‐loading of DOX and PL into ZM‐based nanoplatforms amplifies intracellular oxidative stress, leading to the death of cancer cells as well as the induction and clearance of senescent cells, thereby mitigating the long‐term pro‐tumorigenic risks associated with persistent SASP. Importantly, the biomimetic ZIF‐8@MnOx nanoplatform, which facilitates targeted drug delivery, ensures that DOX and PL are concentrated within the tumor, minimizing systemic toxicity. The redox‐sensitive MnOx component, which undergoes degradation under tumor‐relevant conditions, ROS, and activates the STING signaling pathway and further potentiating antitumor immunity. Moreover, the combination of DOX and PL synergistically enhances immune activation by promoting ICD, which facilitates DC maturation. The activation of the STING signaling pathway further amplifies immune responses, driving effective antitumor immunity. The addition of PL selectively eliminates senescent cells, mitigating chronic inflammation and SASP‐driven tumor progression.

In murine HCC models, mPDZM preferentially accumulates in the tumor and demonstrates significant tumor suppression both in normal and senescent models, with minimal systemic toxicity. Within TME, mPDZM offers a powerful approach to reprogram the TME, not only by killing the tumor cells but also by promoting the recruitment of immune effector cells such as CD8⁺ T cells and NK cells, and reducing immunosuppressive Tregs and M2‐like macrophages. mPDZM further potentiates the antitumor efficacy of anti‐PD‐L1 therapy by enhancing antitumor immunity and inducing a potent abscopal effect.

The rational design of mPDZM represents a significant advance in senescence‐based therapy and offers a means to harness the tumor‐suppressive effects of senescence while addressing the immune evasion and pro‐tumorigenic risks associated with persistent senescent cells. The ability to selectively induce senescence and clear senescent tumor cells, combined with immune activation and redox modulation, positions mPDZM as a promising candidate for clinical translation. Future studies should focus on optimizing the formulation, exploring its application in other solid tumors, and evaluating its potential in combination with additional immune therapies to further enhance its clinical efficacy.

## 4 Experimental Section

### Reagents and Materials

Zinc acetate dihydrate (Zn (OAc)_2_·2H_2_O, Z110777) and 2‐aminobenzimidazole (A104845) were purchased from Aladdin Reagent Co., Ltd. (Shanghai, China). Dialysis membranes and Cy5 were obtained from Sangon Biotechnology. Piperlongumine (HY‐N2329), chlorpromazine (HY‐12708), dynasore (HY‐15304), N‐acetylcysteine (HY‐B0215), palbociclib (HY‐50767) were purchased from MedChemExpress (MCE, USA). Doxorubicin (S1208), z‐VAD‐fmk (S7023) were obtained from Selleck. Sodium azide (26628‐22‐8) was purchased from Sigma–Aldrich. Hydrogen peroxide (H_2_O_2_) and methylene blue (MB) were purchased from commercial suppliers. Dulbecco's modified Eagle medium (DMEM), RPMI 1640 medium, and fetal bovine serum (FBS) were obtained from Gibco Life Sciences (USA). 3‐(4,5)‐Dimethylthiazol‐2‐yl)‐2,5‐diphenyltetrazolium bromide (MTT), Annexin V–FITC apoptosis detection kit (C1062L), BCA protein assay kit (P0009), enhanced mitochondrial membrane potential assay kit with JC‐1 (C2003S), Calcein/PI cell viability/cytotoxicity assay kit (C2015S), mitochondrial superoxide assay kit with MitoSO Red (S0061S), reactive oxygen species assay kit (S0033S), HMGB1 ELISA kit (PH406), TUNEL apoptosis assay kit (C1086) and DAPI staining solution (C1006) were purchased from Beyotime Biotechnology (Shanghai, China). Senescence‐associated β‐galactosidase (SA‐β‐Gal) staining kit (G1580), ATP content assay kit (BC0300), glutathione (GSH) content assay kit (BC117), and GSH (CAS: 70‐18‐8), lymphocyte separation medium (P8860) were obtained from Solarbio (Beijing, China). Recombinant IL‐4 and recombinant mouse M‐CSF were purchased from BioLegend (USA). ELISA kits for IL‐6, IL‐8, CXCL10, IL‐12p70, IL‐10, and TNF‐α were obtained from Yisheng Biotechnology Co., Ltd. (Shanghai, China). Primary antibodies against active caspase‐3 (A11021), H2AX (A11412), and p16 (A23882) were purchased from Abclonal (Wuhan, China). Antibodies against Ki67 (27309‐1‐AP), pRb (30376‐1‐AP), CRT (10292‐1‐AP), and GAPDH (60004‐1‐Ig) were obtained from Proteintech (Wuhan, China). Antibodies against STING (13647), p‐STING (72971), TBK1 (3504), p‐TBK1 (5483), IRF3 (4302), and p‐IRF3 (4947) were purchased from Cell Signaling Technology (CST, USA). Flow cytometric antibodies including anti‐CD45 (103132), anti‐F4/80 (123108), anti‐CD86 (159204), anti‐CD206 (141708), anti‐CD11c (117306), anti‐CD80 (104714), anti‐CD3 (100222), anti‐CD4 (100412), anti‐CD8a (100708), anti‐FOXP3 (126404), anti‐IFN‐γ (163514), and anti‐NK1.1 (156526) were purchased from BioLegend (USA). Anti‐PD‐L1 antibody (αPD‐L1, BE0101) was purchased from BioXCell (USA).

### Cell Culture

HCCLM3(TCHu270, CSTR:19375.09.3101HUMTCHu270) and Hepa1‐6 (SCSP‐512, CSTR:19375.09.3101MOUSCSP512) were obtained from the Cell Bank of the Chinese Academy of Sciences (Shanghai, China). HCCLM3 cells were cultured in DMEM, and Hepa1‐6 cells were cultured in RPMI‐1640 medium, both supplemented with 10% FBS and 1% penicillin‐streptomycin (Gibco, USA). Cells were maintained at 37 °C in a humidified incubator with 5% CO_2_. The culture medium was changed every 2–3 days, and cells were passaged at 80–90% confluency.

### The Synergistic Effect of DOX and PL

The synergistic effects of DOX and PL were evaluated in HCCLM3 and Hepa1‐6 cells using the MTT assay. Briefly, cells were seeded in 96‐well plates and incubated overnight to allow for attachment. DOX and PL were prepared at maximum concentrations of 20 and 40 µm, respectively, and serially diluted in a two‐fold manner to generate a range of concentrations. Cells were treated with the indicated concentrations for 24 h. Then, 20 µL of MTT solution (5 mg mL^−1^) was added to each well and incubated for 4 h at 37 °C. The medium was then carefully removed, and 120 µL of DMSO was added to dissolve the resulting formazan crystals. Absorbance at 570 nm was measured using a microplate reader. Cell viability was calculated relative to the untreated control group, and IC_50_ values were determined using nonlinear regression analysis.

To assess the synergistic effect, subtoxic concentrations of DOX and PL that individually caused ≈15% growth inhibition were selected for subsequent validation experiments. The combined cytotoxicity of DOX and PL was compared with that of each monotherapy using both the MTT assay and live/dead cell staining. For live/dead cell staining, treated cells were incubated with 2 µm Calcein‐AM and 4.5 µm propidium iodide (PI) for 30 min at 37 °C in the dark. Then, the live cells stained with Calcein‐AM (green fluorescence) and dead cells stained with PI (red fluorescence) were immediately observed under a fluorescence microscope. Representative images were captured, and the percentage of live and dead cells was quantified.

Subsequently, the synergistic effects of DOX and PL on ROS accumulation and mitochondrial damage were examined in HCCLM3 and Hepa1‐6 cells treated with either monotherapy or combination therapy. For intracellular ROS detection, a DCFH‐DA ROS detection kit was employed according to the manufacturer's instructions. Briefly, the treated cells were washed with PBS and incubated with 10 µm DCFH‐DA in serum‐free medium at 37 °C for 20 min in the dark. Then, the fluorescence intensity was immediately quantified using a flow cytometer (excitation/emission: 488/525 nm). For mitochondrial membrane potential assessment, a JC‐1 assay kit was used according to the manufacturer's protocol. After incubation with the JC‐1 working solution at 37 °C for 20 min in the dark, the cells were immediately observed under a fluorescence microscope. Polarized mitochondria were characterized by red fluorescence (JC‐1 aggregates), whereas depolarized mitochondria exhibited green fluorescence (JC‐1 monomers).

### Elimination of DOX‐Induced Senescence by PL

To induce cellular senescence, HCCLM3 and Hepa1‐6 cells were treated with 0.25 µg mL^−1^ DOX. SA‐β‐gal staining was performed to validate the successful induction of cellular senescence. Briefly, cells were washed with PBS and fixed with the fixative solution for 15 min at room temperature. After fixation, cells were incubated with the SA‐β‐gal staining solution at 37 °C in the dark. Blue‐stained cells were identified as senescent under a bright‐field microscope.

To investigate the ability of PL to eliminate senescent cells, a series of assays, including SA‐β‐gal staining, apoptosis detection, mitochondrial dysfunction assessment, and Western blot analysis, were conducted in DOX‐induced senescent cells. Non‐senescent cells without DOX pretreatment were used as a baseline control. Briefly, the non‐senescent cells and senescent cells were treated with PL, and SA‐β‐gal staining was subsequently performed to assess residual senescence.

For apoptosis analysis, cells treated with PL were stained with 10 µL Annexin V‐FITC and 5 µL propidium iodide (PI) at room temperature in the dark for 20 min. Apoptotic cells were quantified using flow cytometry.

For mitochondrial dysfunction assessment, cells treated with PL were incubated with 5 µm Mito‐SOX (for mitochondrial ROS detection) or JC‐1 working solution (for mitochondrial membrane potential detection) at 37 °C for 20 min in the dark. After washing, flow cytometry was used to quantify fluorescence intensity and evaluate mitochondrial oxidative stress and depolarization.

For Western blot analysis, cells were lysed using RIPA buffer supplemented with protease and phosphatase inhibitors. The total protein concentration was determined using a BCA protein assay kit. Equal amounts of protein (20 µg per lane) were separated by SDS‐PAGE and transferred onto PVDF membranes. After blocking with 5% non‐fat milk for 1 h at room temperature, the membranes were incubated overnight at 4 °C with primary antibodies against p16, pRb, cleaved caspase‐3, GAPDH. The membranes were then incubated with appropriate HRP‐conjugated secondary antibodies, and protein bands were visualized using enhanced chemiluminescence reagents.

### Synthesis of ZIF‐8 Nanoparticles

ZIF‐8 nanoparticles were synthesized via a simple room‐temperature precipitation method. Briefly, 148.8 mg of (Zn (OAc)_2_·2H_2_O and 328 mg of 2‐methylimidazole (2‐MIM) were separately dissolved in 10 mL of methanol. The 2‐MIM solution was then quickly added to the (Zn (OAc)_2_·2H_2_O solution under vigorous stirring. The 2‐MIM solution was then quickly added to the (Zn (OAc)_2_·2H_2_O solution under vigorous stirring. The resulting white precipitate was collected by centrifugation at 12,000 rpm for 10 min, washed three times with methanol, and dried under vacuum at 60 °C overnight. The obtained ZIF‐8 nanoparticles were stored at room temperature for further use.

### Synthesis of ZIF‐8@MnOx Nanoparticles

To prepare ZIF‐8@MnOx (abbreviated as ZM), 10 mg of ZIF‐8 was dispersed in 10 mL of deionized water by sonication for 15 min. Then, 1 mL of an aqueous KMnO_4_ solution (5 mg mL^−1^) was slowly added dropwise under continuous stirring at room temperature. The mixture was stirred for an additional 2 h to allow in situ deposition of MnOx on the ZIF‐8 surface. After the reaction, the resulting ZM nanoparticles were collected by centrifugation at 12,000 rpm for 10 min, washed three times with deionized water and ethanol, and dried under vacuum at 60 °C overnight.

### Loading of PL and DOX into ZIF‐8@MnOx Nanoparticles

To prepare PL/DOX@ZIF‐8@MnOx (abbreviated as PDZM), 10 mg of ZIF‐8@MnOx nanoparticles were dispersed in 10 mL of deionized water under sonication for 10 min. Subsequently, 1 mL of DOX solution (10 mg mL^−1^) and 1 mL of PL solution (10 mg mL^−1^) were added dropwise into the suspension under gentle stirring. The mixture was stirred continuously at room temperature for 12 h to facilitate drug loading via electrostatic adsorption and pore encapsulation. After incubation, the resulting PDZM nanoparticles were collected by centrifugation at 12,000 rpm for 10 min, washed three times with deionized water to remove unbound drugs, and lyophilized for further use. To obtain DOX‐ or PL‐loaded nanoparticles individually, the same procedure was applied using either DOX or PL alone, denoted as DOX@ZIF‐8@MnOx and PL@ZIF‐8@MnOx, respectively.

### Cell Membrane Coating of PL/DOX@ZIF‐8@MnOx Nanoparticles

To prepare CM‐coated PL/DOX@ZIF‐8@MnOx nanoparticles (abbreviated as mPDZM), cell membranes were first extracted from Hepa1‐6 cells using a membrane protein extraction kit according to the manufacturer's instructions. Briefly, collected cells were subjected to repeated freeze–thaw cycles and mechanical disruption, followed by differential centrifugation to isolate purified membrane vesicles. The protein concentration of the obtained membrane fragments was quantified using a BCA assay. Next, 1 mg of PDZM nanoparticles was mixed with the extracted membrane vesicles (containing ≈100 µg membrane protein) in 1 mL of PBS and sonicated using a probe sonicator to promote membrane fusion. The resulting mPDZM nanoparticles were collected by centrifugation at 12,000 rpm for 10 min, washed with PBS to remove excess membrane materials, and stored at 4 °C for further use.

### Characterization of the Synthesized Nanoparticles

The morphology and structure of the synthesized nanoparticles were characterized using transmission electron microscopy (TEM) and scanning electron microscopy (SEM). Elemental mapping was performed to assess the spatial distribution of key elements (Mn, O, C, N, P) within the nanoparticles. The successful coating of cell membranes onto PDZM was verified by SDS‐PAGE and by TEM with negative staining. The hydrodynamic diameter and surface zeta potential were measured by dynamic light scattering (DLS). X‐ray diffraction (XRD) was conducted to assess the crystalline structure of ZIF‐8 and its derivatives. Fourier transform infrared (FTIR) spectroscopy was performed in the range of 4000–400 cm^−1^ to identify functional groups and confirm the presence of MnOx and loaded drugs. X‐ray photoelectron spectroscopy (XPS) was used to examine surface elemental composition and chemical states, with characteristic peaks corresponding to Zn 2p, Mn 2p, O 1s, N 1s, and C 1s. Ultraviolet–visible (UV–vis) absorption spectra were recorded using a UV–vis spectrophotometerin the range of 200–800 nm to verify the successful encapsulation of DOX and PL based on their specific absorption peaks.

### Drug Loading and Encapsulation Efficiency

The loading efficiency and encapsulation efficiency of DOX and PL in the nanoparticles were determined by UV–vis spectrophotometry. Briefly, 10 mg mPDZM was dispersed in pH 1.0 hydrochloric acid solution to release the encapsulated drugs completely. The supernatant was collected for analysis. The absorbance of DOX and PL was measured at 480 nm and 332 nm, respectively, and the concentrations were calculated based on standard calibration curves. Drug loading (%) = (Weight of loaded drug / Weight of drug‐loaded nanoparticles) × 100%. Encapsulation efficiency (%) = (Weight of loaded drug / Total amount of drug added) × 100%

### In Vitro Drug Release

The pH‐responsive release behaviors of DOX and PL from mPDZM nanoparticles were investigated using a dialysis method. Briefly, 50 mg of mPDZM was dispersed in 10 mL of PBS (pH 7.4, 6.5, 5.0) and loaded into a dialysis bag (molecular weight cutoff: 3.5 kDa). The dialysis bag was immersed in 50 mL of the corresponding PBS buffer and incubated at 37 °C with gentle shaking. At predetermined time intervals, 1 mL of the external buffer was withdrawn and replaced with an equal volume of fresh buffer to maintain sink conditions. The concentration of released DOX and PL in the collected samples was measured by UV–vis spectrophotometry at 480 nm and 332 nm, respectively. ICP‐MS was used to test the concentration of Mn^2+^. The cumulative release percentage was calculated and plotted as a function of time to evaluate the pH‐dependent release profiles.

### Stability Analysis

The stability of ZIF‐8 and mPDZM nanoparticles was evaluated in three different media, including double‐distilled water (DDW), phosphate‐buffered saline (PBS, pH 7.4), and PBS supplemented with 10% FBS. Briefly, nanoparticles were dispersed in each medium at a concentration of 0.1 mg mL^−1^ and incubated at 37 °C for up to 7 days. The hydrodynamic diameter was measured using DLS to assess particle size stability.

### H_2_O_2_ consumption

The H_2_O_2_‐scavenging ability of the nanoparticles was evaluated using a UV–vis spectrophotometric method. Briefly, H_2_O_2_ solution (100 µm) was prepared in PBS (pH 7.4), and 1 mL of this solution was incubated with 1 mL of PBS, ZM, or mPDZM suspension at 37 °C. After incubation for 0.1,0.5, 1, 2, 3, 4, 6, 8 h, the mixtures were centrifuged, and the supernatants were collected. The residual H_2_O_2_ concentration was quantified by measuring absorbance at 240 nm using a UV–vis spectrophotometer.

### Oxygen Generation

The oxygen generation ability of the nanoparticles was evaluated using an oxygen probe. Briefly, 2 mL of 100 µm H_2_O_2_ solution was incubated with 1 mL of PBS, ZM, or mPDZM suspension at 37 °C. ZM or mPDZM was dissolved in the aqueous solution as a control. After incubation, the oxygen concentration in the mixtures was monitored using an oxygen probe. The data were analyzed to evaluate the oxygen generation efficiency of the different groups.

### Extracellular Hydroxyl Radical Generation

The hydroxyl radical generation ability of mPDZM was evaluated using the 3‐(4,5‐dimethylthiazol‐2‐yl)‐2,5‐diphenyltetrazolium bromide (MB) assay. Briefly, mPDZM at different concentrations was mixed with a reaction mixture containing 50 µm MB, 25 mm NaHCO_3_ (5% CO_2_ atmosphere), and 20 mm of H_2_O_2_ at 37 °C for 60 min. The degradation of MB, indicative of hydroxyl radical generation, was assessed by measuring the absorbance at 665 nm using a UV–vis spectrophotometer. To assess the GSH‐responsive regulation of hydroxyl radical generation, mPDZM was preincubated with different concentrations of GSH in 25 mm NaHCO_3_ (5% CO_2_ atmosphere) at 37 °C for 60 min. The supernatant obtained after centrifugation was subsequently reacted with 50 µm MB and 20 mm H_2_O_2_ in the NaHCO_3_ (5% CO_2_ atmosphere) at 37 °C for 30 min. The absorbance was recorded using a UV–vis spectrophotometer.

### Extracellular GSH depletion

To evaluate the GSH‐depleting capability of mPDZM, different concentrations of the nanoparticles were incubated with 1 mm GSH at 37 °C for 60 min. Then, 100 µL of DTNB solution (2.5 mg mL^−1^) was added to each sample, and the mixture was gently shaken at room temperature for 5 min. The absorbance was then measured at 412 nm using a UV–vis spectrophotometer to quantify the remaining GSH.

### T_1_‐Weighted Magnetic Resonance (MR) Imaging

To evaluate the T_1_‐weighted MR performance of mPDZM, aqueous dispersions of the nanoparticles with varying Mn concentrations were incubated with 1 mm GSH at 37 °C for 10 min. Then, the samples were transferred into 1.5 mL Eppendorf tubes and scanned using a 3.0 T clinical MR system. T_1_‐weighted images were acquired under standard imaging parameters, and signal intensities were quantified to assess the GSH‐responsive MR enhancement capability of mPDZM.

### Cellular Uptake Behaviors of mPDZM

The cellular uptake behavior of mPDZM was investigated in Hepa1‐6 cells. Cells were seeded in six‐well plates at a density of 2 × 10^5^ cells per well and cultured overnight. The cells were then incubated with Cy5‐labeled mPDZM for various time intervals. At each time point, cells were washed three times with cold PBS to remove excess nanoparticles, fixed with 4% paraformaldehyde for 15 min, and stained with DAPI. Intracellular Cy5 fluorescence was observed using a fluorescence microscope. For quantification, Cy5 fluorescence was measured using a flow cytometer. To investigate the cellular uptake mechanism, Hepa1‐6 cells were pretreated with specific endocytic inhibitors, 10 mm sodium azide, 10 µg mL^−1^ chlorpromazine, or 80 µm dynasore at 37 °C for 30 min, prior to incubation with Cy5‐labeled mPDZM. Then, Cy5 fluorescence was analyzed by a flow cytometer to assess the impact of each inhibitor on mPDZM internalization.

### In Vitro Validation of the Antitumor Effect of ZM‐Based Nanoparticles

The antitumor activity of ZM‐based nanoparticles was assessed in vitro using Hepa1‐6 cells. Cells were seeded in 96‐well plates at a density of 5 × 10^3^ cells per well and allowed to adhere overnight. Cells were exposed to ZM‐based formulations (ZM, DZM, mPDZM), and free DOX was included as a reference treatment. Cell viability was measured using the MTT assay. Absorbance was read at 570 nm using a microplate reader, and the IC_50_ was then calculated.

Furthermore, live/dead cell staining was conducted after treatment with PBS, free DOX, ZM, DZM, PDZM, or mPDZM at equivalent drug concentrations to validate the cytotoxic effects. In addition, cells seeded in six‐well plates with the indicated treatments were harvested and processed for apoptosis analysis and Western blotting. Apoptotic cells were quantified by Annexin V‐FITC/PI double staining and analyzed using flow cytometry. Western blotting was performed to detect the expression of cleaved caspase‐3 using a specific primary antibody (1:1000 dilution, Cat. No. 25128‐1‐AP, Proteintech).

### Intracellular Redox Homeostasis Disruption Capacity of ZM‐Based Nanoparticles

To evaluate the redox homeostasis disruption capacity of ZM‐based nanoparticles, intracellular ROS content, superoxide dismutase (SOD) activity, and GSH levels were measured. Briefly, 2 × 10^5^ cells per well were seeded into six‐well plates. Then the cells were treated with PBS, free DOX, ZM, DZM, PDZM, or mPDZM. Then, the treated cells were harvested for further analysis. Flow cytometry was used to measure the ROS content using the DCFH‐DA probe. For assessment of a SOD Activity and GSH levels, the treated cells were lysed with RIPA buffer. For the assessment of SOD activity and GSH levels, the treated cells were lysed with RIPA buffer. The lysates were collected and centrifuged at 12 000 × g for 10 min at 4 °C to collect the supernatant. Then, the SOD Activity Assay Kit (WST‐8 method) and the Glutathione Reductase Activity Assay Kit (DTNB method) were used to assess SOD activity and GSH levels, according to the manufacturer's instructions.

To validate the role of redox homeostasis disruption induced by mPDZM in regulating cell growth, cells were pretreated with N‐acetylcysteine (NAC, 5 mm), glutathione (GSH, 5 mm), or the pan‐caspase inhibitor z‐VAD‐fmk (20 µm) for 1 h prior to mPDZM exposure. After mPDZM treatment, cell viability was assessed using the MTT assay, as previously described.

### Senolytic Capacity of ZM‐Based Nanoparticles

To test the senolytic capacity of ZM‐based nanoparticles, DOX‐induced senescent Hepa1‐6 cells were used. Briefly, 2 × 10^5^ Hepa1‐6 cells were seeded into six‐well plates and treated with 0.25 µg mL^−1^ DOX for 3 days to induce cellular senescence. Afterward, the medium was refreshed, and the cells were treated with PBS, free DOX, ZM, DZM, PDZM, or mPDZM for another 48 h. Subsequently, senescent cells were stained using a senescence β‐galactosidase staining kit (Beyotime, C0602) by adding 1 mL of the staining solution (containing X‐gal substrate) to the cells and incubating at 37 °C for 12 h. The senescent cells were then observed under a light microscope.

To evaluate the expression of senescence‐related proteins, the treated cells were collected and lysed using RIPA lysis buffer supplemented with 1% PMSF and phosphatase inhibitors on ice for 30 min. Total protein was quantified by the BCA protein assay, and 20 µg of protein was loaded per lane for SDS‐PAGE. The primary antibodies used in this experiment were p16, DcR, and p53. For the detection of the senescence‐associated secretory phenotype (SASP), cell culture supernatants were collected after treatment and centrifuged at 3000 rpm for 10 min to remove debris. The concentrations of IL‐6, IL‐8, and IL1β were determined using commercial ELISA kits according to the manufacturers’ protocols.

To determine whether the senolytic activity of mPDZM is mediated through apoptosis, senescent cells were pretreated with the pan‐caspase inhibitor z‐VAD‐fmk (20 µm) for 1 h prior to mPDZM exposure. Western blot analysis was performed to test the expression of cleaved‐caspase3 and activation of NF‐κB pathway in mPDZM‐treated cells. In addition, free PL, the Dasatinib plus Quercetin (D+Q) combination, and ABT263 were included as reference senolytics to compare their senescent cell‐clearing efficiencies with that of mPDZM. Following treatment, senescent cells were subjected to SA‐β‐gal staining using a commercial senescence β‐galactosidase assay kit.

### RNA Sequencing Analysis

A total of 2 ×  10^5^ Hepa1‐6 cells were seeded into six‐well plates were treated with PBS and mPDZM. Total RNA was extracted using TRIzol reagent (Thermo Fisher Scientific, Shanghai, China) according to the manufacturer's instructions. High‐throughput RNA sequencing was conducted by BGI‐Tech (Shenzhen, China) using the BGISEQ‐500 sequencing platform. Gene expression levels were quantified as FPKM (Fragments Per Kilobase of transcript per Million mapped reads). Differentially expressed genes (DEGs) between groups were identified using DESeq2, with a threshold of | log_2_(fold change) | ≥ 1.5 and adjusted *p*‐value < 0.05. Functional enrichment, including Gene Ontology (GO) and Kyoto Encyclopedia of Genes and Genomes (KEGG) pathway analyses, was conducted using the Dr. Tom analysis platform provided by BGI‐Tech (Shenzhen, China).

### Chemokine Expression Analysis

A total of 2 × 10^5^ Hepa1‐6 cells were seeded into six‐well plates. Cells were then treated with PBS, free DOX, ZM, DZM, PDZM, or mPDZM. Then, total RNA was extracted using TRIzol reagent (Thermo Fisher Scientific, Shanghai, China) according to the manufacturer's protocol. The concentration and purity of RNA were assessed, and 1 µg of total RNA was reverse‐transcribed into cDNA using a PrimeScript RT reagent kit (Takara, Japan). Quantitative real‐time PCR (qRT‐PCR) was performed using SYBR Green Master Mix (Applied Biosystems) on a QuantStudio 5 Real‐Time PCR System (Thermo Fisher Scientific). The expression levels of chemokines including CCL5, CCL6, CCL9, CCL20, CXCL10, and CXCL11 were analyzed. GAPDH was used as the internal reference gene. Relative gene expression was calculated using the 2−^ΔΔCt^ method.

### Immunogenic Cell Death (ICD) Induction Analysis

To evaluate the ability of various treatments to induce ICD, key ICD markers, including CRT exposure, ATP release, and HMGB1 release, were assessed. Briefly, Hepa1‐6 cells were treated with the indicated treatment. IF analysis was performed to evaluate CRT exposure. The anti‐CRT primary antibody was diluted at 1:200 in blocking buffer. The fluorescence of CRT was visualized using a laser scanning confocal microscope and quantified by flow cytometry. The release of HMGB1 and ATP into the cell culture supernatant was determined using commercial ELISA kits.

### Dendritic Cell Maturation and T Cell Activation

To assess the effect of treatments on dendritic cell maturation, bone marrow‐derived dendritic cells (BMDCs) were generated from C57BL/6 mice. Briefly, bone marrow cells were flushed from femurs and tibias and cultured in RPMI‐1640 medium supplemented with 10% FBS, 20 ng mL^−1^ GM‐CSF, and 10 ng mL^−1^ IL‐4 at 37 °C in 5% CO_2_. On days 3 and 5, half of the medium was replaced with fresh cytokine‐containing medium. On day 6, immature BMDCs were collected and co‐incubated with dying Hepa1‐6 cells pre‐treated with PBS, DOX, ZM, DZM, or mPDZM at a 1:1 ratio for 24 h in fresh medium. Then, cells were harvested and stained with fluorescently labeled antibodies against CD11c, CD80, and CD86 for 30 min at 4 °C in the dark. The percentage of mature DCs (CD80⁺CD86⁺) was analyzed using flow cytometry (BD FACSCanto II), and data were processed using FlowJo software. The concentrations of IL‐12p70 and IL‐10 in the culture supernatants were determined using commercial ELISA kits, according to the manufacturers’ protocols. Absorbance was measured at 450 nm using a microplate reader, and cytokine levels were calculated based on standard curves. In addition, western blot analysis was performed to test the activation of the STING pathway in BMDCs with mPDMZ treatment.

To evaluate whether mPDZM‐induced immunogenic signals promote tumor antigen‐specific CD8⁺ T‐cell activation, an OT‐I T‐cell/BMDCs co‐culture system was established. Briefly, BMDCs were generated from femurs and tibias of C57BL/6 mice by culturing bone marrow cells in RPMI‐1640 supplemented with 10% FBS, 1% penicillin–streptomycin, and GM‐CSF (20 ng mL^−1^) for 7 days, with medium refreshed every 2–3 days. Hepa1‐6 cells were treated with PBS (control), free drugs, or mPDZM for 24 h. Cell culture supernatants were then collected, centrifuged to remove debris, and passed through a 0.22 µm filter. BMDCs were pulsed with OVA_257‐264_ (SIINFEKL) peptide (1 µg mL^−1^) for 2 h and subsequently incubated with the indicated tumor cell supernatants for an additional 12–18 h. Surface expression of the H‐2KbSIINFEKL complex on BMDCs was analyzed by flow cytometry using a specific H‐2KbSIINFEKL antibody. CD8^+^ T cells were isolated from the spleens of OT‐I transgenic mice using a CD8^+^ T‐cell isolation kit according to the manufacturer's instructions and labeled with CFSE to monitor proliferation. CFSE‐labeled CD8^+^ T cells were co‐cultured with pretreated BMDCs at a DC: T‐cell ratio of 1:5 for 72 h. T‐cell proliferation was assessed by CFSE dilution using flow cytometry, and CD8^+^IFN‐γ^+^ cells were quantified.

### Animal Experiments

All animal experiments were performed in compliance with institutional ethical standards and were approved by the Institutional Animal Care and Use Committee of the Fifth Affiliated Hospital of Wenzhou Medical University. C57BL/6 mice were purchased from Shanghai SLAC Laboratory Animal Co., Ltd. (Shanghai, China). To establish a subcutaneous tumor model, mice were anesthetized, and 1 × 10^6^ Hepa1‐6 cells suspended in 100 µL of PBS were injected subcutaneously. Tumor growth was monitored by measuring tumor length (L) and width (W) using a caliper, and tumor volume was calculated using the formula: V = 0.5 × L × W^2^. Once tumor volumes reached ≈1200 mm^3^, the mice were anesthetized and humanely sacrificed in accordance with ethical guidelines.

### In Vivo Biodistribution Assessment

To investigate the biodistribution profile of the nanoparticles, C57BL/6 mice bearing Hepa1‐6 tumors with volumes ranging from ≈100–200 mm^3^ were randomly assigned to receive either free Cy5 or Cy5‐labeled mPDZM nanoparticles (100 µL per mouse). In vivo fluorescence imaging was performed post‐injection using an in vivo imaging system (IVIS Lumina III, PerkinElmer, USA). Mice were anesthetized with isoflurane during imaging, and fluorescence signals were captured in the near‐infrared channel corresponding to Cy5. For biodistribution analysis, mice were euthanized at 24 h post‐injection, and major organs, including the heart, liver, spleen, lungs, kidneys, and tumors, were harvested. Ex vivo fluorescence images were acquired. Quantification of fluorescence intensity was conducted using living image software.

### In Vivo T1‐Weighted MR Imaging

To assess the in vivo T1‐weighted imaging capability of the nanoparticles, Hepa1‐6 tumor‐bearing C57BL/6 mice were intravenously injected with mPDZM via the tail vein. MR scans were performed using a clinical 3.0 T Siemens MR system. T1‐weighted images were acquired. Typical acquisition parameters were: TR = 500 ms, TE = 10 ms, slice thickness = 1.0 mm, field of view (FOV) = 30 × 30 mm, matrix = 256 × 256. Signal intensity within the tumor region was analyzed.

### In Vivo Safety Assessment

Hepa1‐6 tumor‐bearing C57BL/6 mice were treated with mPDZM at escalating doses of 100 and 200 mg kg^−1^, administered via tail vein injection. At the end of the treatment cycle, blood samples were collected for hematological and biochemical analyses. Liver and kidney function were evaluated by measuring serum levels of alanine aminotransferase (ALT) and creatinine (CR). Hematological parameters, including white blood cells (WBC), red blood cells (RBC), and platelets (PLT), were also assessed. Major organs, including the heart, liver, spleen, lungs, and kidneys, were harvested, fixed in 4% paraformaldehyde, embedded in paraffin, sectioned, and stained with hematoxylin and eosin (H&E) for histopathological evaluation under a light microscope. Any signs of inflammation, necrosis, or tissue damage were recorded to assess systemic toxicity.

### Antitumor Efficacy

Hepa1‐6 tumor‐bearing C57BL/6 mice were used. Mice with tumors reaching ≈100 mm^3^ in volume were selected and randomly assigned to five treatment groups: PBS, DOX, ZM, DZM, PDZM, or mPDZM. All treatments were administered via intravenous injection through the tail vein at equivalent DOX doses every two days for a total of 6 days. Tumor dimensions were measured using a digital caliper. Mice were monitored for body weight and general condition. At the end of a 2‐week treatment period, mice were euthanized, and tumors were collected, photographed, weighed, and subjected to further analyses, including immunohistochemistry or Western blotting. In parallel, an additional cohort of tumor‐bearing mice receiving the same treatments was monitored over time to assess overall survival. Mice were observed daily, and survival time was recorded until death or when humane endpoints were reached. Survival was analyzed and presented using Kaplan–Meier survival curves, and statistical significance between groups was determined using the log‐rank (Mantel–Cox) test.

Next, the antitumor efficacy of the formulations was further evaluated in a senescence‐enriched tumor model. Briefly, therapy‐induced senescence was established by administering low‐dose DOX to induce a senescent tumor microenvironment. After three cycles of DOX treatment, tumor‐bearing mice were randomly assigned to five groups and treated with PBS, free DOX, ZM, DZM, PDZM, or mPDZM. Tumor growth was monitored over time. At the end of the treatment period, mice were euthanized, and tumors were collected, weighed, and analyzed by p16 immunohistochemistry and ELISA for intra‐tumoral SASP factors. In a separate cohort, overall survival was recorded to assess long‐term therapeutic benefit.

For validation, therapy‐induced senescence was established using palbociclib, a selective CDK4/6 inhibitor. Mice received a dose of palbociclib (50 mg kg^−1^) to induce a senescence‐enriched tumor. Then, mice were randomly assigned to five treatment groups and subsequently administered PBS, free DOX, ZM, DZM, PDZM, or mPDZM. Tumor growth was monitored over time, and in a separate cohort, overall survival was recorded to assess long‐term therapeutic benefit.

### Histological Analysis

To evaluate ROS levels in tumor tissues, DCFH‐DA was used. A ROS‐sensitive fluorescent probe was used. Fresh tumor tissues were harvested and immediately snap‐frozen in OCT embedding medium. Frozen sections were prepared using a cryostat, air‐dried, and incubated with 10 µm DCFH‐DA in serum‐free medium at 37 °C for 30 min in the dark. For the assessment of Ki67, HIF‐1α, and CRT expression, tumor tissues were fixed in 4% paraformaldehyde overnight at 4 °C, embedded in paraffin, and sectioned at 5 µm thickness. Immunohistochemistry was performed using specific primary antibodies against Ki67, HIF‐1α, and CRT. For HIF‐1α detection, a fluorescent HRP‐conjugated secondary antibody was used. For CRT and Ki67, detection was carried out using a biotin‐avidin‐horseradish peroxidase/3,3′‐diaminobenzidine (biotin‐avidin‐HRP/DAB) detection system, and signals were developed using DAB substrate and counterstained with hematoxylin. Cell apoptosis was evaluated using the TUNEL assay according to the manufacturer's instructions. Additionally, p16 expression was examined by IHC to confirm the presence of senescent cells, and tumor lysates were subjected to ELISA for quantification of SASP, including IL‐6, IL8, and IL1β.

Tumor and major organ tissues (heart, liver, spleen, lung, and kidney) were harvested for H&E staining. Tumors were fixed in 4% paraformaldehyde overnight at 4 °C, embedded in paraffin, and sectioned at 5 µm thickness. Sections were deparaffinized, rehydrated through graded ethanol solutions, and stained with hematoxylin for 5 min followed by eosin for 2 min. After dehydration and mounting, tissue morphology was observed under a light microscope to assess histological changes such as inflammation, necrosis, or tissue damage.

### Evaluation of In Vivo Immune Modulatory Effects

After 7 days of treatment, Hepa1‐6 tumor‐bearing mice were euthanized. Tumor‐draining lymph nodes (TDLNs) were collected and processed into single‐cell suspensions for the evaluation of DC maturation, using surface markers such as CD11c, CD80, and CD86, and analyzed by flow cytometry. Simultaneously, tumor tissues were excised, minced, and enzymatically digested into single‐cell suspensions. The resulting cells were stained with fluorochrome‐conjugated antibodies to assess various immune cell populations, including: Helper T cells (CD3^+^ CD4^+^), Tregs (CD3^+^ CD4^+^FoxP3^+^), Cytotoxic T lymphocytes (CTLs) (CD3^+^ CD8^+^), Activated CD8^+^ T cells (IFN‐γ^+^CD8^+^, Granzyme B^+^CD8^+^), NK cells (NK1.1^+^), Macrophage M1 (F4/80^+^CD86^+^) and M2 (F4/80^+^CD206^+^). The gating strategies for flow cytometric analysis of NK cells and Treg cells in tumors, as well as DCs in the TDLNs, are presented in Figure S39 (Supporting Information).

In addition, CyTOF analysis was performed to assess tumor‐infiltrating immune cells. Tumors were excised, minced, and digested in RPMI‐1640 containing collagenase IV, DNase I, and hyaluronidase at 37 °C for 30–40 min. Cell suspensions were filtered through a 70 µm strainer, washed with PBS containing 2% FBS, and subjected to red blood cell lysis. Single cells were counted and adjusted to the required concentration. After Fc‐receptor blocking, cells were stained with a panel of metal‐conjugated antibodies targeting lineage and functional markers. Subsequently, cells were fixed in 1.6% paraformaldehyde and incubated overnight at 4 °C with an Iridium‐based DNA intercalator. Before acquisition, cells were washed, resuspended, and analyzed on a CyTOF mass cytometer following the manufacturer's instructions. Data were processed using unsupervised clustering and dimensionality‐reduction algorithms to identify immune cell subsets, which were annotated based on canonical lineage and functional marker profiles.

### Systemic Antitumor Immune Response and Combination Therapy with Anti‐PD‐L1 antibody

To assess the systemic immune response and the synergistic efficacy of mPDZM combined with anti‐PD‐L1 therapy, a bilateral Hepa1‐6 tumor model was established in C57BL/6 mice. Briefly, 1 × 10^6^ Hepa1‐6 cells were subcutaneously injected into the right flank on day 0 (designated as the primary tumor), followed by injection of the same number of cells into the left hindlimb on day 3 (designated as the distant tumor). Once tumors reached ≈80–100 mm^3^, mice were randomly divided into four groups: PBS, mPDZM, anti‐PD‐L1, and Combination (mPDZM + anti‐PD‐L1) group. mPDZM was administered via intra‐tumoral injection into the primary tumor, and anti‐PD‐L1 antibody (2 mg kg^−1^) was administered via intravenous injection. Tumor volumes were measured every 2 days using a caliper. Both primary and distant tumor growth were recorded to evaluate local and abscopal antitumor effects. Mice were monitored over time for overall survival analysis.

An orthotopic hepatocellular carcinoma model was established to evaluate the effect of mPDZM on spontaneous metastasis. Briefly, male C57BL/6J mice were anesthetized with isoflurane, and a subcostal incision was made to expose the left liver lobe. Hepa1‐6 cells were injected into the liver parenchyma using a 29‐gauge syringe, and gentle pressure was applied for 1–2 min to prevent cell leakage before wound closure. 10 days after implantation, mice were randomized into four groups: PBS, anti‐PD‐L1, mPDZM, and mPDZM + anti‐PD‐L1. Four weeks after tumor inoculation, mice were sacrificed, and lungs were collected to assess spontaneous metastatic dissemination. Tissues were fixed in 4% paraformaldehyde, embedded in paraffin, sectioned at 5 µm, and stained with H&E. Metastatic lesions were identified and quantified by two blinded investigators based on the presence or absence of metastasis, number of metastatic foci, and the metastatic area relative to total tissue area.

To explore treatment‐induced immune modulation, an additional cohort of mice was sacrificed on day 7 after treatment initiation. Tumor tissues were harvested for flow cytometric analysis of immune cell populations, including CD4^+^ T cells, CD8^+^ T cells, Tregs, activated CD8^+^ T cells, and NK cells. Serum levels of antitumor cytokines (IFNγ and TNF‐α) were quantified by ELISA assays according to the manufacturer's protocol.

### Ethics Declaration

This study received approval from the Institutional Review Board of the Fifth Affiliated Hospital of Wenzhou Medical University and was carried out in compliance with the ethical principles stated in the Declaration of Helsinki. All animal procedures were conducted in compliance with the Animal Care and Use Committee of the Fifth Affiliated Hospital of Wenzhou Medical University.

## Conflict of Interest

The authors declare no conflict of interest.

## Author Contributions

S. F., L. Z., and B. L. contributed equally to this work. Shiji Fang, Liyun Zheng, and Bin Lin conducted experiments, gathered and analyzed data, and drafted the manuscript. J.C., D.H., Y.D., M.H., P.Q., M.W., X.G., and Y.Z. assisted with the experiments. G.S. and M.C. assisted in data analysis. Z.Z., Z.L., and J.J. conceived and designed the study, and performed data analysis and interpretation. J.T. performed writing revisions and manuscript formatting. All authors revised and approved the final manuscript.

## Supporting information



Supporting Information

## Data Availability

The data are available from the corresponding author on reasonable request.

## References

[1] F. Bray , M. Laversanne , H. Sung , J. Ferlay , R. L. Siegel , I. Soerjomataram , A. Jemal , CA Cancer J. Clin. 2024, 74, 229. 38572751 10.3322/caac.21834

[2] S. Y. Hwang , P. Danpanichkul , V. Agopian , N. Mehta , N. D. Parikh , G. K. Abou‐Alfa , A. G. Singal , J. D. Yang , Clin. Mol. Hepatol. 2025, 31, S228. 39722614 10.3350/cmh.2024.0824 PMC11925437

[3] European Association for the Study of the Liver ., J. Hepatol. 2025, 82, 315. 36464532 10.1016/j.jhep.2022.10.006

[4] G. Lau , S. Obi , J. Zhou , R. Tateishi , S. Qin , H. Zhao , M. Otsuka , S. Ogasawara , J. George , P. K. H. Chow , J. Cai , S. Shiina , N. Kato , O. Yokosuka , K. Oura , T. Yau , S. L. Chan , M. Kuang , Y. Ueno , M. Chen , A. L. Cheng , G. Cheng , W. L. Chuang , O. Baatarkhuu , F. Bi , Y. Y. Dan , R. A. Gani , A. Tanaka , W. Jafri , J. D. Jia , et al., Hepatol. Int. 2024, 18, 1661. 39570557 10.1007/s12072-024-10732-z

[5] D. H. Palmer , K. Malagari , L. M. Kulik , J. Hepatol. 2020, 72, 277. 31954492 10.1016/j.jhep.2019.09.023

[6] K. C. Lai , Y. H. Chen , Y. P. Hung , N. J. Chiang , M. H. Chen , S. C. Chen , Hepatol. Int. 2024, 18, 1804. 39580565 10.1007/s12072-024-10728-9 PMC11632046

[7] U. Harkus , M. Wankell , P. Palamuthusingam , C. McFarlane , L. Hebbard , Semin. Cancer Biol. 2022, 86, 799. 35065242 10.1016/j.semcancer.2022.01.005

[8] S. Qin , M. Chen , A. L. Cheng , A. O. Kaseb , M. Kudo , H. C. Lee , A. C. Yopp , J. Zhou , L. Wang , X. Wen , J. Heo , W. Y. Tak , S. Nakamura , K. Numata , T. Uguen , D. Hsiehchen , E. Cha , S. P. Hack , Q. Lian , N. Ma , J. H. Spahn , Y. Wang , C. Wu , P. K. H. Chow , Lancet 2023, 402, 1835.37871608

[9] L. Rimassa , R. S. Finn , B. Sangro , J. Hepatol. 2023, 79, 506. 36933770 10.1016/j.jhep.2023.03.003

[10] A. J. Schoenfeld , M. D. Hellmann , Cancer Cell 2020, 37, 443. 32289269 10.1016/j.ccell.2020.03.017 PMC7182070

[11] M. Pinter , B. Scheiner , D. J. Pinato , Gastroenterol. Hepatol. 2023, 8, 760. 10.1016/S2468-1253(23)00147-4 37327807

[12] C. Qin , G. Yang , Q. Wei , H. Xin , J. Ding , X. Chen , Chem. Res. Chin. Univ. 2025, 41, 1.

[13] C. Wang , H. Yu , X. Yang , X. Zhang , Y. Wang , T. Gu , S. Zhang , C. Luo , Asian J. Pharm. Sci. 2022, 17, 412. 35782326 10.1016/j.ajps.2022.02.004 PMC9237584

[14] Y. Sun , X. Feng , C. Wan , J. F. Lovell , H. Jin , J. Ding , Asian J. Pharm. Sci. 2021, 16, 129. 33995609 10.1016/j.ajps.2020.05.004 PMC8105413

[15] D. V. Krysko , A. D. Garg , A. Kaczmarek , O. Krysko , P. Agostinis , P. Vandenabeele , Nat. Rev. Cancer 2012, 12, 860. 23151605 10.1038/nrc3380

[16] G. Kroemer , C. Galassi , L. Zitvogel , L. Galluzzi , Nat. Immunol. 2022, 23, 487. 35145297 10.1038/s41590-022-01132-2

[17] W. Li , J. Yang , L. Luo , M. Jiang , B. Qin , H. Yin , C. Zhu , X. Yuan , J. Zhang , Z. Luo , Y. Du , Q. Li , Y. Lou , Y. Qiu , J. You , Nat. Commun. 2019, 10, 3349. 31350406 10.1038/s41467-019-11269-8 PMC6659660

[18] Z. Xu , J. Xu , S. Sun , W. Lin , Y. Li , Q. Lu , F. Li , Z. Yang , Y. Lu , W. Liu , Redox Biol. 2022, 54, 102351. 35671636 10.1016/j.redox.2022.102351 PMC9168183

[19] Z. Li , Z. Chu , J. Yang , H. Qian , J. Xu , B. Chen , T. Tian , H. Chen , Y. Xu , F. Wang , ACS Nano 2022, 16, 15471. 35981098 10.1021/acsnano.2c08013

[20] X. Li , Y. F. Pan , Y. B. Chen , Q. Q. Wan , Y. K. Lin , T. Y. Shang , M. Y. Xu , T. Y. Jiang , M. M. Pei , Y. X. Tan , L. W. Dong , X. Y. Wan , Cell Death Dis. 2024, 15, 300. 38684648 10.1038/s41419-024-06685-8 PMC11058202

[21] D. McHugh , I. Duran , J. Gil , Nat. Rev. Drug Discov. 2025, 24, 57. 39548312 10.1038/s41573-024-01074-4

[22] K. Shimizu , H. Inuzuka , F. Tokunaga , Semin. Cancer Biol. 2025, 108, 1. 39557316 10.1016/j.semcancer.2024.11.001

[23] L. Zhou , B. Ma , M. Ruscetti , Trends Cancer 2025, 11, 334. 39732594 10.1016/j.trecan.2024.11.010 PMC11981858

[24] F. Xing , H. Lv , W. Xiang , L. Wang , Q. Zong , G. Lv , C. Liu , Q. Feng , H. Wang , W. Yang , Cancer Lett. 2025, 627, 217544. 39929434 10.1016/j.canlet.2025.217544

[25] D. Wu , C. Zhang , G. Liao , K. Leng , B. Dong , Y. Yu , H. Tai , L. Huang , F. Luo , B. Zhang , T. Zhan , Q. Hu , S. Tai , Cell. Mol. Biol. Lett. 2022, 27, 105. 36447138 10.1186/s11658-022-00403-y PMC9707060

[26] M. Chen , W. Chen , S. Sun , Y. Lu , G. Wu , H. Xu , H. Yang , C. Li , W. He , M. Xu , X. Li , D. Jiang , Y. Cai , C. Liu , W. Zhang , Z. He , J. Adv. Res. 2025, 73, 357. 39218249 10.1016/j.jare.2024.08.034 PMC12225934

[27] G. Balamurli , A. Q. X. Liew , W. W. Tee , S. Pervaiz , Redox Biol. 2024, 78, 103441. 39612910 10.1016/j.redox.2024.103441 PMC11629570

[28] L. Chibaya , J. Snyder , M. Ruscetti , Semin. Cancer Biol. 2022, 86, 827. 35143990 10.1016/j.semcancer.2022.02.005 PMC9357237

[29] H. J. Hwang , D. Kang , J. Shin , J. Jung , S. Ko , K. H. Jung , S. S. Hong , J. E. Park , M. J. Oh , H. J. An , W. H. Yang , Y. G. Ko , J. H. Cha , J. S. Lee , Nat. Commun. 2025, 16, 353. 39753537 10.1038/s41467-024-54132-1 PMC11699195

[30] N. Rosenberg , M. Van Haele , T. Lanton , N. Brashi , Z. Bromberg , H. Adler , H. Giladi , A. Peled , D. S. Goldenberg , J. H. Axelrod , A. Simerzin , C. Chai , M. Paldor , A. Markezana , D. Yaish , Z. Shemulian , D. Gross , S. Barnoy , M. Gefen , O. Amran , S. Claerhout , M. Fernandez‐Vaquero , M. Garcia‐Beccaria , D. Heide , M. Shoshkes‐Carmel , D. Schmidt Arras , S. Elgavish , Y. Nevo , H. Benyamini , J. E. E. Tirnitz‐Parker , et al., J Hepatol 2022, 77, 1631. 35988690 10.1016/j.jhep.2022.07.029

[31] L. Wang , L. Lankhorst , R. Bernards , Nat. Rev. Cancer 2022, 22, 340. 35241831 10.1038/s41568-022-00450-9

[32] C. Wang , S. Vegna , H. Jin , B. Benedict , C. Lieftink , C. Ramirez , R. L. de Oliveira , B. Morris , J. Gadiot , W. Wang , A. du Chatinier , L. Wang , D. Gao , B. Evers , G. Jin , Z. Xue , A. Schepers , F. Jochems , A. M. Sanchez , S. Mainardi , H. Te Riele , R. L. Beijersbergen , W. Qin , L. Akkari , R. Bernards , Nature 2019, 574, 268. 31578521 10.1038/s41586-019-1607-3 PMC6858884

[33] Y. Zhang , B. Xiao , S. Yuan , L. Ding , Y. Pan , Y. Jiang , S. Sun , X. Ke , L. Cai , L. Jia , Redox Biol. 2024, 76, 103323. 39180983 10.1016/j.redox.2024.103323 PMC11388193

[34] H. Sabit , T. M. Pawlik , F. Radwan , M. Abdel‐Hakeem , S. Abdel‐Ghany , A. S. Wadan , M. Elzawahri , A. El‐Hashash , B. Arneth , Mol Cancer 2025, 24, 160. 40457437 10.1186/s12943-025-02357-z PMC12131435

[35] Y. Wang , Y. Tang , L. Guo , X. Yang , S. Wu , Y. Yue , C. Xu , Asian J. Pharm. Sci. 2025, 20, 101017. 39931355 10.1016/j.ajps.2025.101017 PMC11808527

[36] X. Hu , H. Zhu , Y. Shen , L. Rao , J. Li , X. He , X. Xu , J. Nanobiotechnol. 2025, 23, 131. 10.1186/s12951-025-03229-w PMC11844015 39979917

[37] Y. Wan , J. Fang , Y. Wang , J. Sun , Y. Sun , X. Sun , M. Qi , W. Li , C. Li , Y. Zhou , L. Xu , B. Dong , L. Wang , Adv. Healthcare Mater. 2021, 10, 2101515. 10.1002/adhm.202101515 34558227

[38] Z. Deng , M. Xi , C. Zhang , X. Wu , Q. Li , C. Wang , H. Fang , G. Sun , Y. Zhang , G. Yang , Z. Liu , ACS Nano 2023, 17, 4495. 36848115 10.1021/acsnano.2c10352

[39] S. G. Balwe , D. Moon , M. Hong , J. M. Song , Nano Converg 2024, 11, 48. 39604693 10.1186/s40580-024-00456-z PMC11602914

[40] L. Hou , C. Tian , Y. Yan , L. Zhang , H. Zhang , Z. Zhang , ACS Nano 2020, 14, 3927. 32298077 10.1021/acsnano.9b06111

[41] G. Zhao , S. Wang , G. Nie , N. Li , Med 2024, 5, 660. 38582088 10.1016/j.medj.2024.03.012

[42] M. Karabicici , S. Alptekin , Z. Firtina Karagonlar , E. Erdal , Mol. Oncol. 2021, 15, 2185. 33524223 10.1002/1878-0261.12916 PMC8334288

[43] M. Kciuk , A. Gielecinska , S. Mujwar , D. Kolat , Z. Kaluzinska‐Kolat , I. Celik , R. Kontek , Cells 2023, 12, 659. 37048059 10.3390/cells12070986 PMC10092955

[44] S. K. Tripathi , B. K. Biswal , Pharmacol. Res. 2020, 156, 104772. 32283222 10.1016/j.phrs.2020.104772

[45] L. Zheng , S. Fang , A. Chen , W. Chen , E. Qiao , M. Chen , G. Shu , D. Zhang , C. Kong , Q. Weng , S. Xu , Z. Zhao , J. Ji , Pharmacol. Res. 2022, 177, 106140. 35202819 10.1016/j.phrs.2022.106140

[46] F. Liu , Q. Zhou , H. F. Jiang , T. T. Zhang , C. Miao , X. H. Xu , J. X. Wu , S. L. Yin , S. J. Xu , J. Y. Peng , P. P. Gao , X. Cao , F. Pan , X. He , X. Q. Chen , J. Exp. Clin. Cancer Res. 2023, 42, 118. 37161450 10.1186/s13046-023-02686-1 PMC10170830

[47] F. Liu , Q. Xiang , Y. Luo , Y. Luo , W. Luo , Q. Xie , J. Fan , H. Ran , Z. Wang , Y. Sun , J. Nanobiotechnol. 2023, 21, 165. 10.1186/s12951-023-01932-0 PMC10207777 37221521

[48] A. Basu , Pharmacol. Ther. 2022, 230, 107943. 34182005 10.1016/j.pharmthera.2021.107943

[49] Q. Zhang , W. Chen , X. Lv , Q. Weng , M. Chen , R. Cui , G. Liang , J. Ji , Front. Pharmacol. 2019, 10, 1180. 31680962 10.3389/fphar.2019.01180 PMC6802400

[50] X. Yan , C. Chen , Y. Ren , T. Su , H. Chen , D. Yu , Y. Huang , M. Chao , G. Wu , G. Jiang , F. Gao , Acta Biomater. 2024, 188, 329. 39278301 10.1016/j.actbio.2024.09.015

[51] H. Wang , Y. Yu , R. Li , H. Zhang , Z. S. Chen , C. Sun , J. Zhuang , Acta Pharm. Sin. B 2025, 15, 4476. 41049742 10.1016/j.apsb.2025.07.022 PMC12491696

[52] E. Limagne , L. Nuttin , M. Thibaudin , E. Jacquin , R. Aucagne , M. Bon , S. Revy , R. Barnestein , E. Ballot , C. Truntzer , V. Derangere , J. D. Fumet , C. Latour , C. Rebe , P. S. Bellaye , C. G. Kaderbhai , A. Spill , B. Collin , M. B. Callanan , A. Lagrange , L. Favier , B. Coudert , L. Arnould , S. Ladoire , B. Routy , P. Joubert , F. Ghiringhelli , Cancer Cell 2022, 40, 136. 35051357 10.1016/j.ccell.2021.12.009

[53] K. Sasaki , S. Nishina , A. Yamauchi , K. Fukuda , Y. Hara , M. Yamamura , K. Egashira , K. Hino , Cell Mol. Gastroenterol. Hepatol. 2021, 11, 739. 33191170 10.1016/j.jcmgh.2020.10.010 PMC7841526

[54] S. Zhang , L. Kang , X. Dai , J. Chen , Z. Chen , M. Wang , H. Jiang , X. Wang , S. Bu , X. Liu , G. Zhang , H. Tang , Free Radical Biol. Med. 2022, 193, 202. 36228830 10.1016/j.freeradbiomed.2022.10.004

[55] Y. Tan , Q. Zhu , M. Yang , F. Yang , Q. Zeng , Z. Jiang , D. Li , Pharmacol Res. 2024, 207, 107314. 39059614 10.1016/j.phrs.2024.107314

[56] J. Sun , Z. Zhao , X. Wei , J. Yang , D. Li , M. Li , Y. E. Choonara , L. Chen , J. Ding , X. Chen , Biomaterials 2026, 324, 123488. 40554219 10.1016/j.biomaterials.2025.123488

[57] Q. Wei , H. Xin , X. Wang , C. Qin , Y. Su , D. Li , J. Ding , Chin. Chem. Lett. 2025, 36, 111477.

[58] Y. Liu , Y. Pan , W. Cao , F. Xia , B. Liu , J. Niu , G. Alfranca , X. Sun , L. Ma , J. M. de la Fuente , J. Song , J. Ni , D. Cui , Theranostics 2019, 9, 6867. 31660074 10.7150/thno.37586 PMC6815945

[59] X. An , W. Yu , J. Liu , D. Tang , L. Yang , X. Chen , Cell Death Dis. 2024, 15, 556. 39090114 10.1038/s41419-024-06939-5 PMC11294602

[60] J. D. Hayes , A. T. Dinkova‐Kostova , K. D. Tew , Cancer Cell 2020, 38, 167. 32649885 10.1016/j.ccell.2020.06.001 PMC7439808

[61] J. Da , H. Hu , L. Wang , Z. Wang , H. Chen , Y. Xie , T. Li , J. Wang , M. Zhong , W. Dang , Y. Liu , W. Tan , Nano Lett. 2025, 25, 8033. 40326155 10.1021/acs.nanolett.5c01741

[62] H. Zhang , X. Xu , X. Shou , W. Liao , C. Jin , C. Chen , C. Zhang , W. Gao , J. Zhang , W. Ge , L. Shi , Adv. Healthcare Mater. 2024, 13, 2401085. 10.1002/adhm.202401085 38796738

[63] A. Estepa‐Fernandez , A. Garcia‐Fernandez , A. Lerida‐Viso , J. F. Blandez , I. Galiana , F. Sancenon‐Galarza , M. Orzaez , R. Martinez‐Manez , Pharmacol Res 2023, 187, 106628. 36566002 10.1016/j.phrs.2022.106628

[64] M. Jiang , P. Chen , L. Wang , W. Li , B. Chen , Y. Liu , H. Wang , S. Zhao , L. Ye , Y. He , C. Zhou , J. Hematol. Oncol. 2020, 13, 81. 32571374 10.1186/s13045-020-00916-z PMC7310007

