# Supplementary material for: Biomimetic Nanomedicine for Senescence‐Modulated Immune Activation Enhances Immunotherapy Efficacy in Hepatocellular Carcinoma
Source: Adv Sci (Weinh). 2025 Dec 23;13(10):e17792. doi: 10.1002/advs.202517792 (PMC12915144; doi:10.1002/advs.202517792)
Supplement: Supplementary file 1 — Supporting Information [file ADVS-13-e17792-s001.docx]

**Supporting information**

**Biomimetic Nanomedicine for Senescence-Modulated Immune Activation Enhances Immunotherapy Efficacy in Hepatocellular Carcinoma**

Shiji Fang^1,2#^, Liyun Zheng^1,3,4#^, Bin Lin^1#^, Jiale Chen^1^, Dehai Hou^1,5^, Yiming Ding^1^, Mengzhu Han^1^, Pan Qin^1^, Mengyuan Wang^1^, Xiaoju, Guo^1^, Yeyu Zhang^1,3^, Gaofeng Shu^1,3,4^, Fazong Wu^1,3^, Jianfei Tu^1,3^, Minjiang Chen^1,3,4^, Zhongwei Zhao^1,3*^, Zhuang Liu^2*^, Jiansong Ji^1,3,4*^

^1^ Zhejiang Key Laboratory of Imaging and Interventional Medicine, the Fifth Affiliated Hospital of Wenzhou Medical University, Lishui 323000, China.

^2^ Institute of Functional Nano & Soft Materials (FUNSOM), Jiangsu Key Laboratory for Carbon-Based Functional Materials & Devices, Soochow University, 215123, Suzhou, China. zliu@suda.edu.cn.

^3^ Cancer center, Lishui Central Hospital, the Fifth Affiliated Hospital of Wenzhou Medical University, Lishui 323000, China.

^4^ Department of Radiology, Lishui Central Hospital, the Fifth Affiliated Hospital of Wenzhou Medical University, Lishui 323000, China.

^5^ School of Pharmacy, Jiamusi University, Jiamusi 154007, China.

***Supporting results***


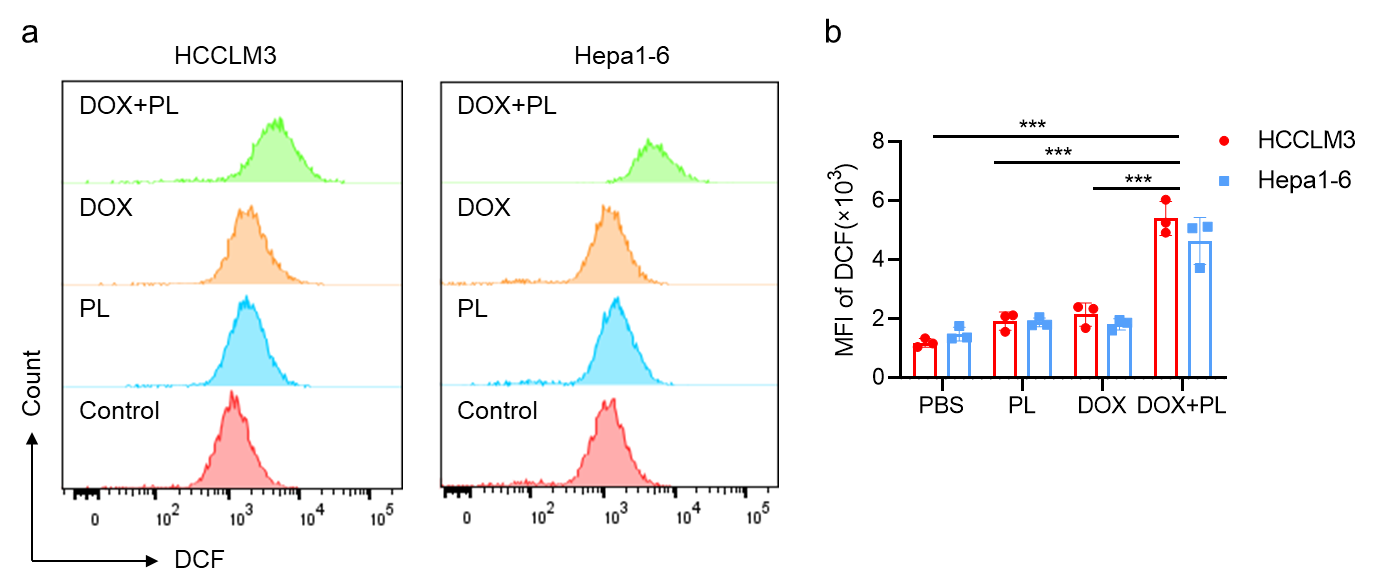


**Figure S1.** Intracellular ROS levels in HCCLM3 and Hepa1-6 cells after treatment with DOX, PL, or DOX + PL, measured by flow cytometry using DCFH-DA staining (a). (b)The quantitative analysis of mean fluorescence intensity (MFI) from three independent experiments were performed. Data are presented as mean ± SD, **p* < 0.05, ***p* < 0.01, ****p*< 0.001 compared to the control group.


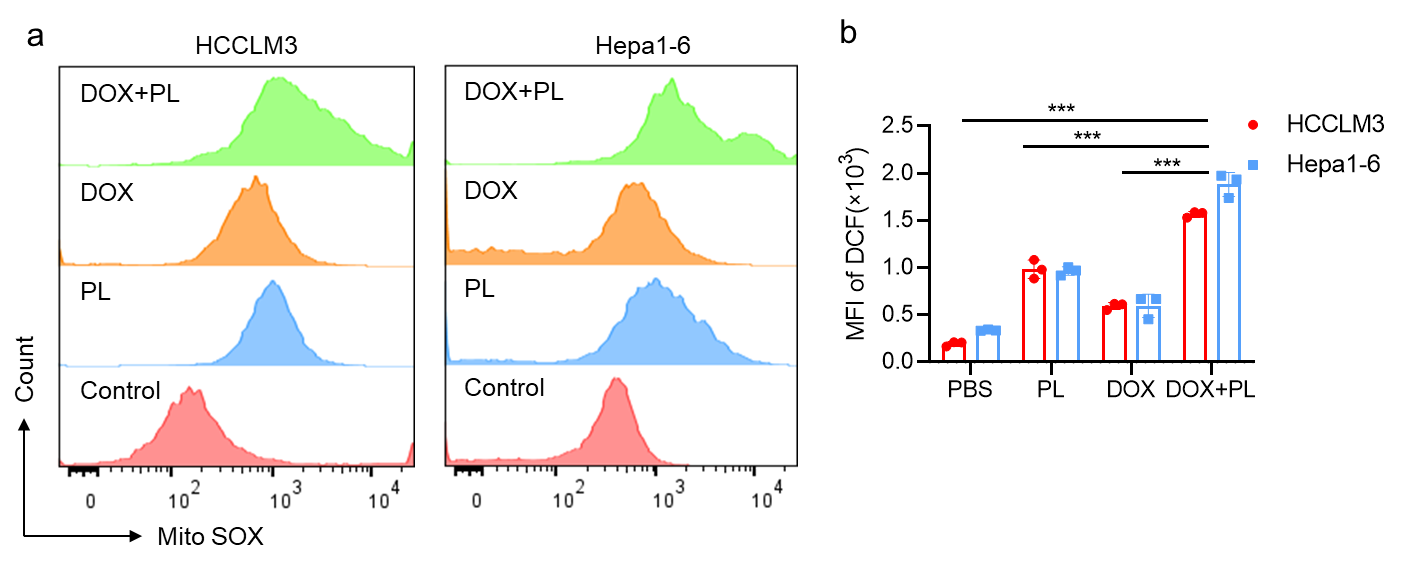


**Figure S2.** Mitochondrial superoxide levels in HCCLM3and Hepa1-6 cells after treatment with DOX, PL, or DOX + PL, measured by flow cytometry using Mito-SOX staining (a). (b)The quantitative analysis of mean fluorescence intensity (MFI) from three independent experiments were performed. Data are presented as mean ± SD, *** < 0.05, **** < 0.01, *****< 0.001 compared to the control group.


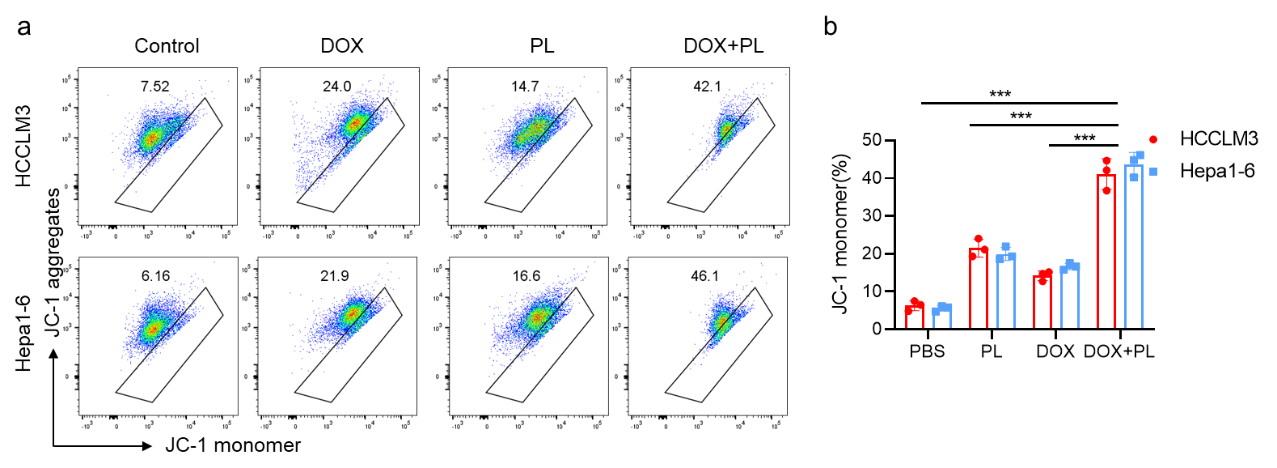


**Figure S3.** Mitochondrial membrane potential in HCCLM3 and Hepa1-6 cells treated with DOX, PL, or DOX + PL, detected by JC-1 staining and flow cytometry (a). The rate of JC-1 monomer was quantified from three independent experiments (b). Data are presented as mean ± SD, **p* < 0.05, ****p** < 0.01, ******p*** < 0.001 compared to the control group.


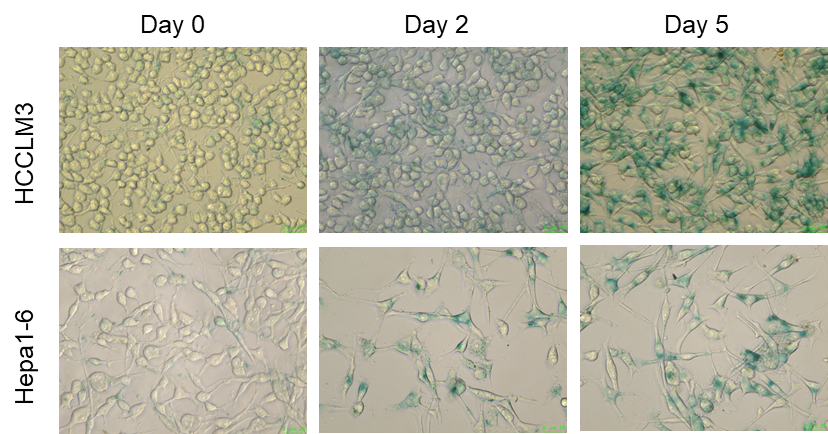


**Figure S4.** Senescence-associated β-galactosidase (SA-β-gal) staining of HCCLM3 and Hepa1-6 cells after treatment with DOX. Cells exhibiting blue staining were identified as senescent. Representative images from three independent experiments are shown. Scale bar = 25 μm.


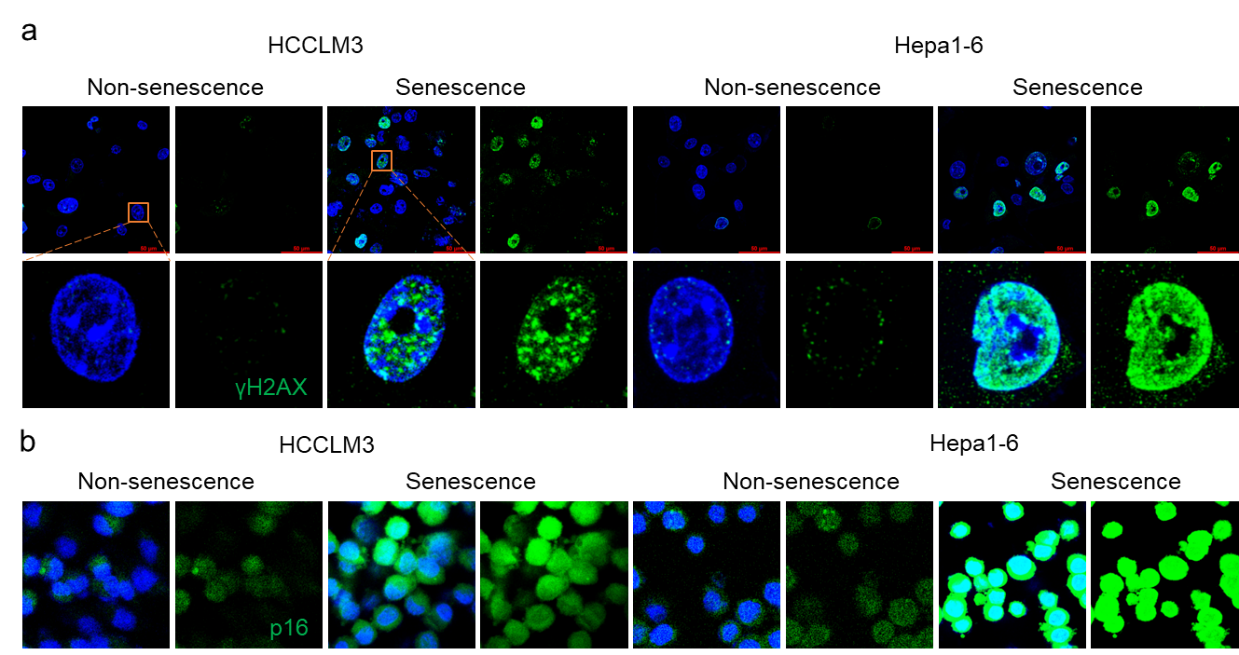


**Figure S5.** Representative immunofluorescence images of γH2AX (a) and p16 (b) in HCCLM3 cells and Hepa1-6 cells treated with PBS or DOX. Nuclei were counterstained with DAPI (blue). Enlarged views of the selected regions are shown below each panel. Scale bars = 50 μm.


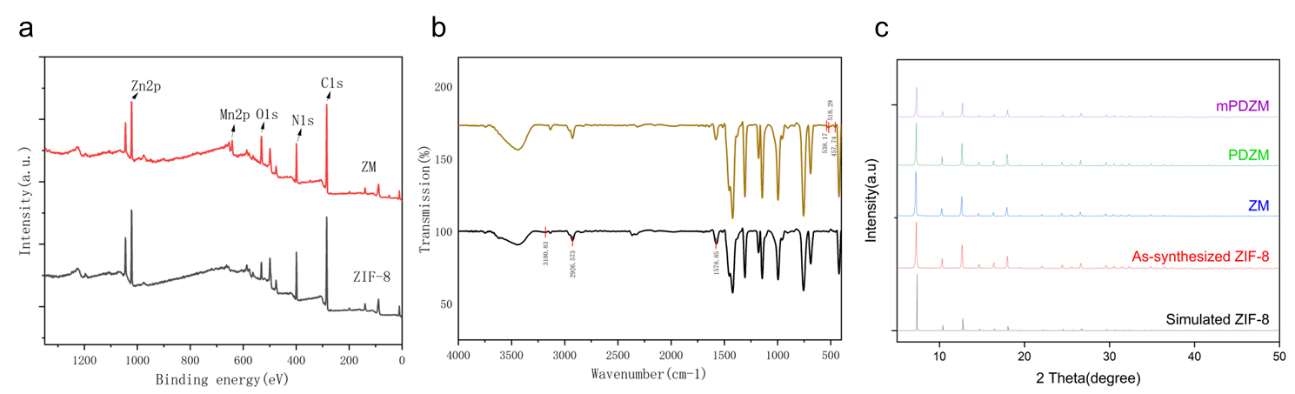


**Figure S6.** Surface composition, chemical structure, and crystallinity of nanoparticles.
(a) XPS survey spectra of ZIF-8 and ZM. (b) FTIR spectra of ZIF-8 and ZM. (c) XRD patterns of simulated ZIF-8, as-synthesized ZIF-8, ZM, PDZM, and mPDZM.


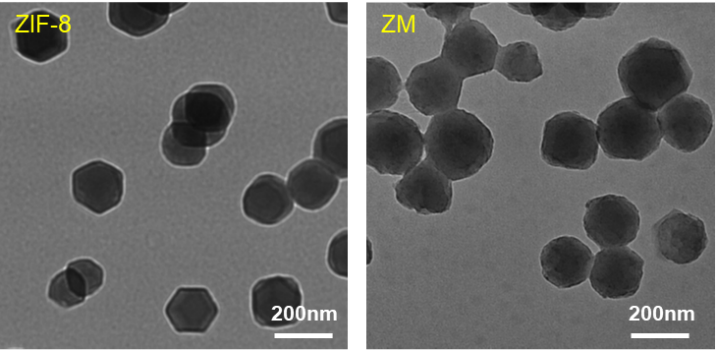


**Figure S7.** TEM images of ZIF-8 and ZM showing the change in particle morphology after MnOx modification. Scale bars: 200 nm.


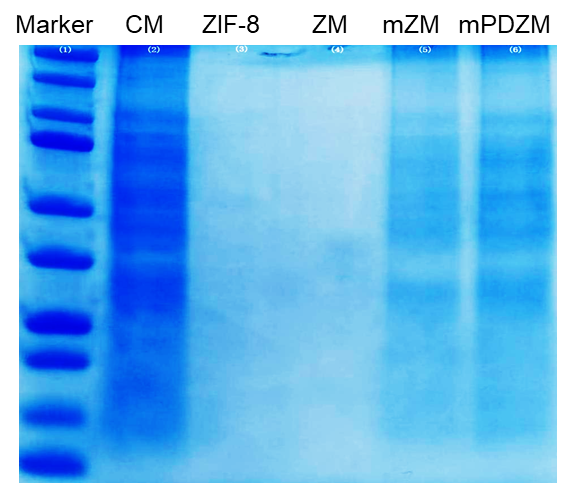


**Figure S8.** SDS-PAGE analysis of proteins from different samples stained with Coomassie Brilliant Blue. Lane 1: protein marker; lane 2: Hepa1-6 cell lysate; lane 3: ZIF-8; lane 4: ZM; lane 5: PDZM; lane 6: mPDZM. The protein bands in lane 6 indicate the successful coating of Hepa1-6 cell membrane onto mPDZM nanoparticles.


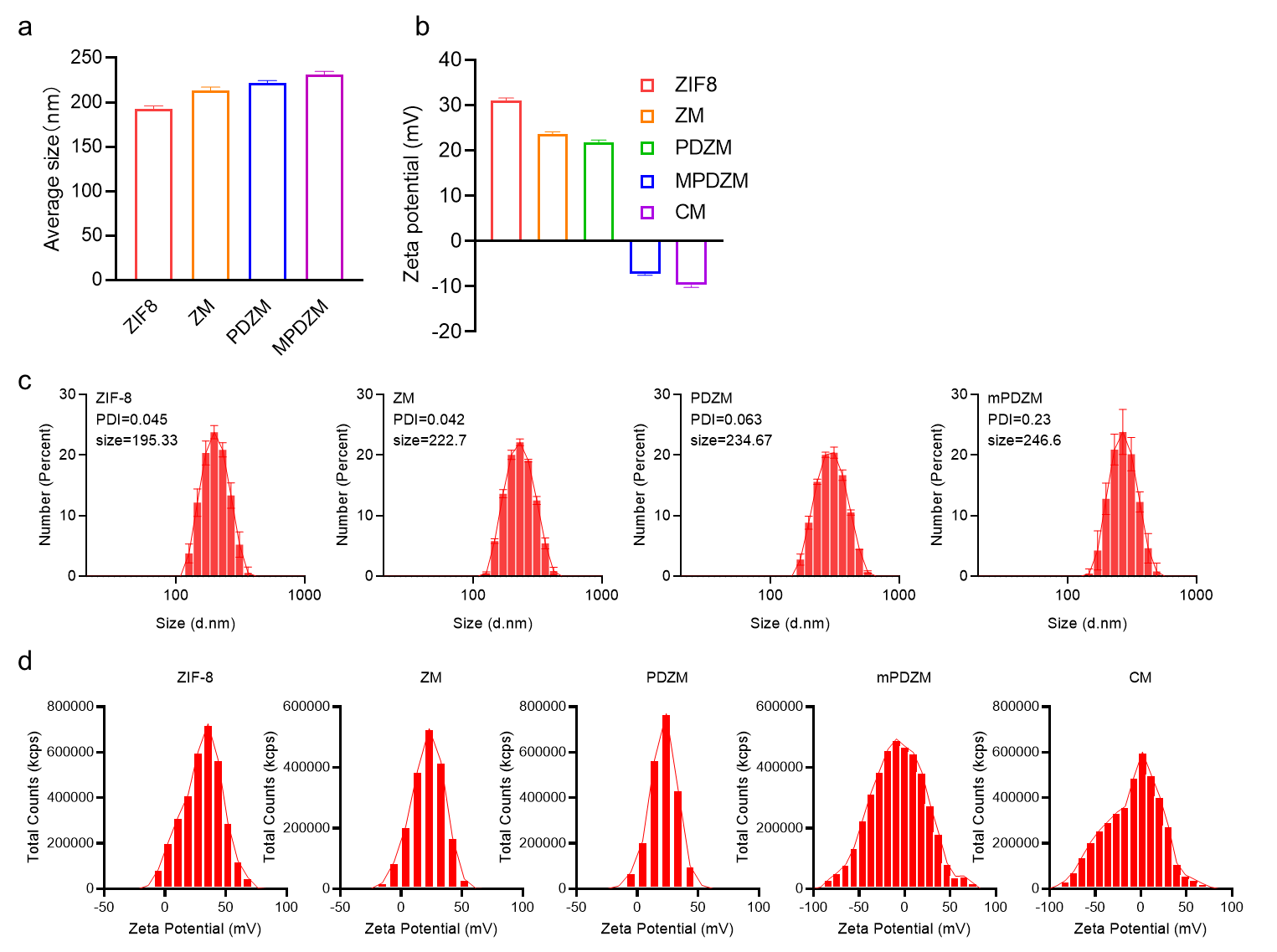


**Figure S9.** Diameter and ζ-Potential of different particles.

(a) Hydrodynamic diameter of ZIF-8, ZM, PDZM, and mPDZM measured by DLS in PBS (pH 7.4, 25 °C). (b) ζ-Potential of the same samples determined by electrophoretic light scattering in PBS (pH 7.4, 25 °C). Data are presented as mean ± SD (n = 3). (c) Size and polydispersity index (PDI) distributions. (d) ζ-Potential of the different nanoparticles and the cell membrane (CM).


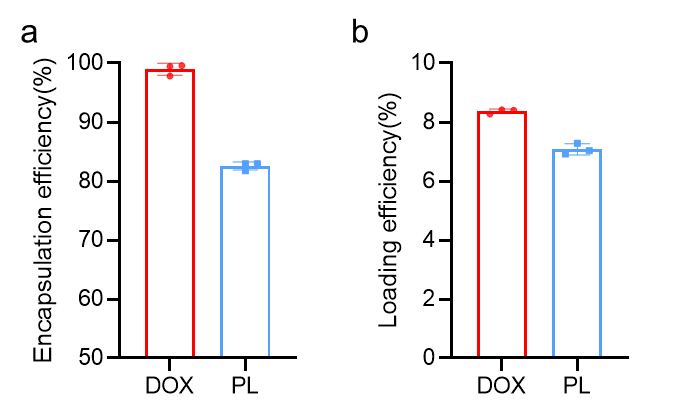


**Figure S10.** Encapsulation efficiency and drug loading efficiency of PDZM and mPDZM. (a) Encapsulation efficiency of DOX and PL in PDZM and mPDZM. (b) Drug loading content of DOX and PL in PDZM and mPDZM. Data are presented as mean ± SD (n = 3).


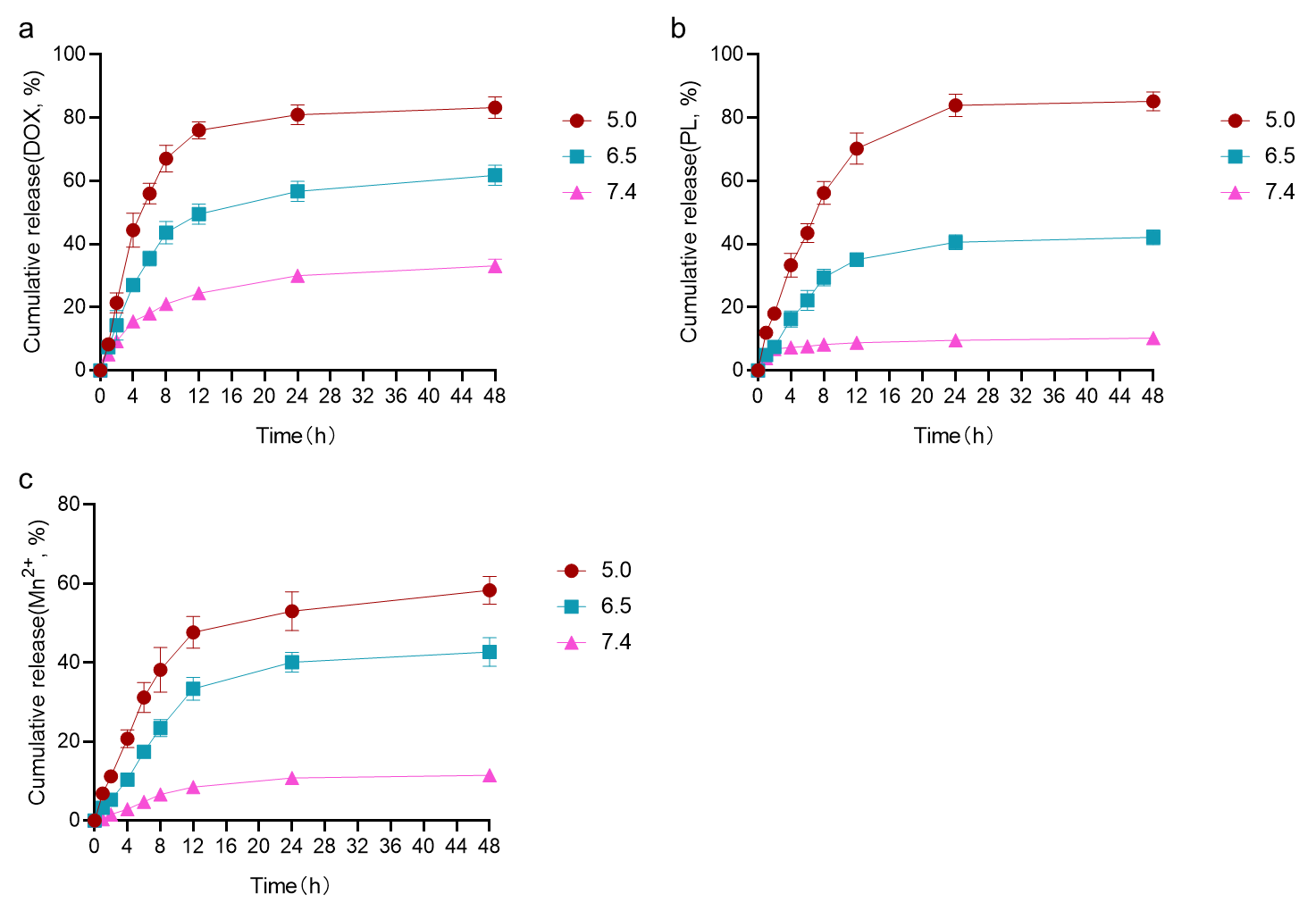


**Figure S11.** Drug release profiles of (a) DOX, (b) PL and (c) Mn^2+^ from mPDZM at different pH values. Release studies were performed in PBS at pH 7.4, 6.5, and 5.0 at 37 °C under gentle shaking, with periodic sampling and replenishment of fresh medium. Drug concentrations were quantified by UV-vis spectroscopy (DOX and PL) and ICP-MS (Mn^2+^). Data are presented as mean ± SD (n = 3).


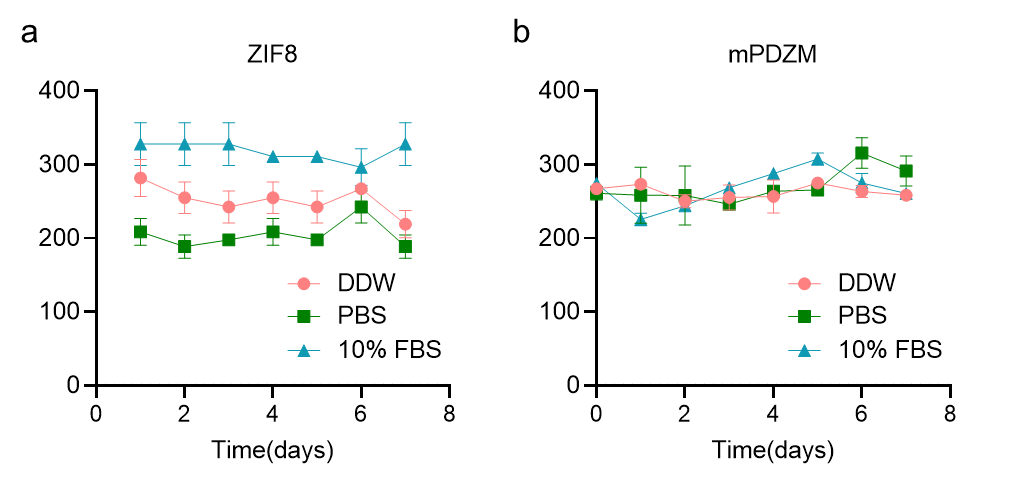


**Figure S12.** Stability of mPDZM nanoparticles. Hydrodynamic diameter of (a)ZIF-8 and (b) mPDZM measured over 7 days in PBS (pH 7.4), DMEM supplemented with 10% FBS, and ultrapure water. Data are presented as mean ± SD (n = 3).


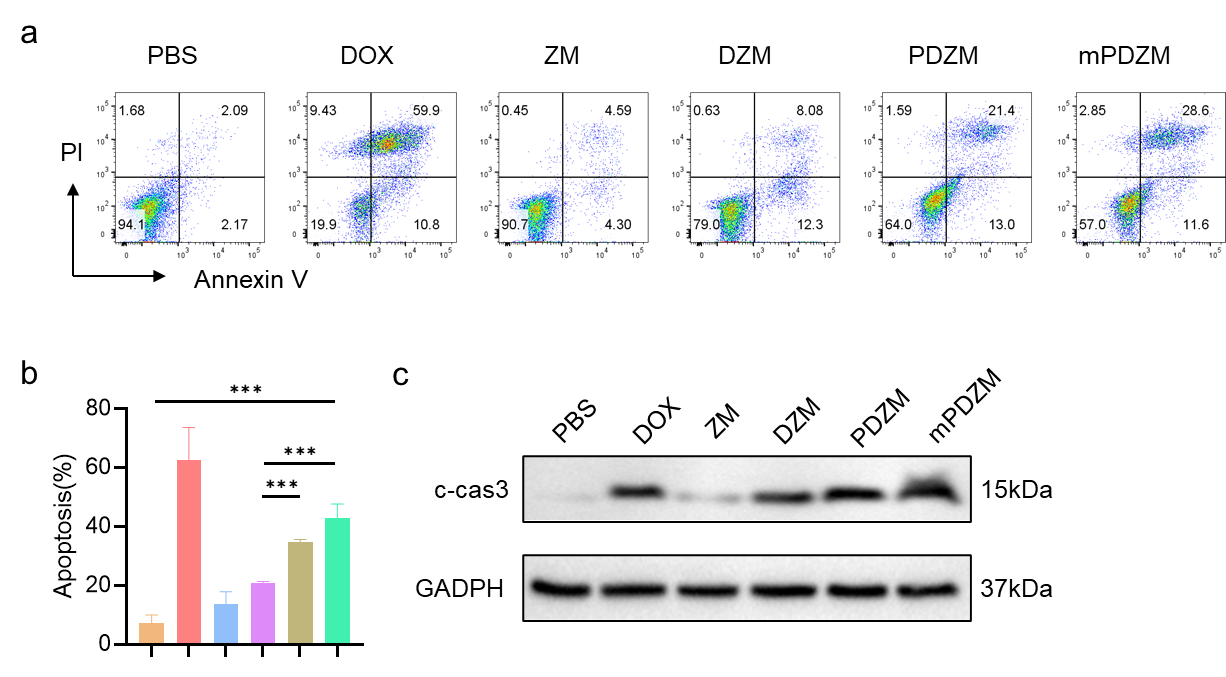


**Figure S13.** Apoptosis analysis of Hepa1-6 cells after different treatments. (a) Flow cytometric analysis using Annexin V-FITC/PI staining to detect apoptotic cells after treatment with PBS, DOX, ZM, DZM, PDZM, or mPDZM. (b) Quantification of total apoptotic cells is shown. (c)Western blot analysis the expression of cleaved-caspase3. Note: c-cas3 represents cleaved-caspase 3. (d) the cell viability of senescent cell with the pan-caspase inhibitor z-VAD-fmk was add Data are presented as mean ± SD (n = 3). Statistical analysis was performed using one-way ANOVA with Tukey’s post hoc test, **p* < 0.05, ***p* < 0.01, ****p* < 0.001.

**Figure S14.** Effects of different inhibitors on mPDZM-induced cytotoxicity. Cell viability of Hepa1-6 cells treated with PBS, mPDZM, mPDZM + NAC (5 mM), mPDZM + GSH (5 mM), or mPDZM + z-VAD-fmk (20 μM) for 24 h, measured by the MTT assay. Statistical analysis was performed using one-way ANOVA with Tukey’s post hoc test, **p* < 0.05, ***p* < 0.01, ****p* < 0.001.


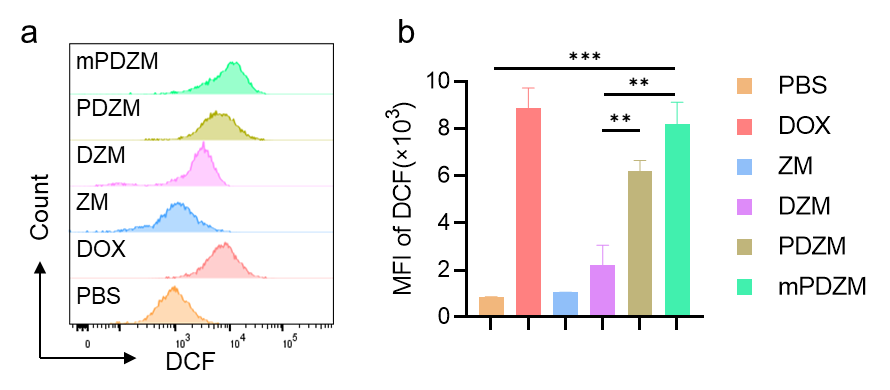


**Figure S15.** Intracellular ROS generation in Hepa1-6 cells after different treatments. Flow cytometric histograms (a) and quantitative analysis (b) of DCF fluorescence intensity in Hepa1-6 cells treated with PBS, DOX, ZM, DZM, PDZM, or mPDZM. ROS levels were detected using DCFH-DA staining, and fluorescence was measured at Ex/Em = 488/525 nm. Statistical analysis was performed using one-way ANOVA with Tukey’s post hoc test, **p* < 0.05, ***p* < 0.01, ****p* < 0.001.


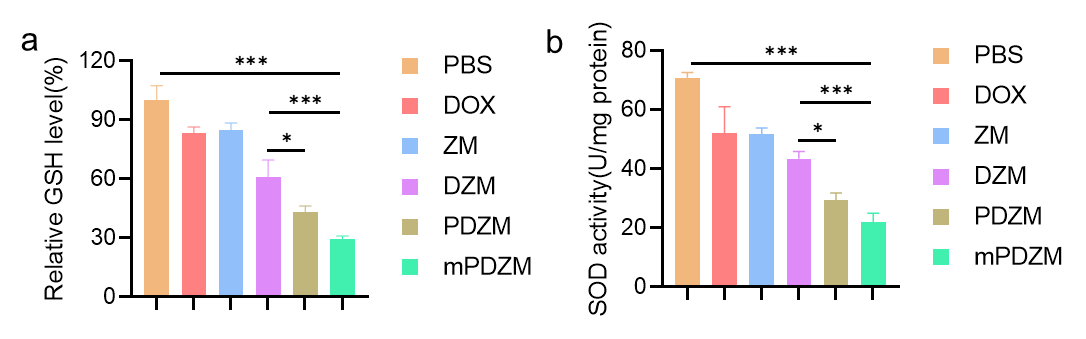


**Figure S16.** Intracellular GSH levels and SOD activity in Hepa1-6 cells after different treatments. (a) GSH levels and (b) SOD activity in Hepa1-6 cells treated with PBS, DOX, ZM, DZM, PDZM, or mPDZM. Statistical analysis was performed using one-way ANOVA with Tukey’s post hoc test, **p* < 0.05, ***p* < 0.01, ****p* < 0.001.


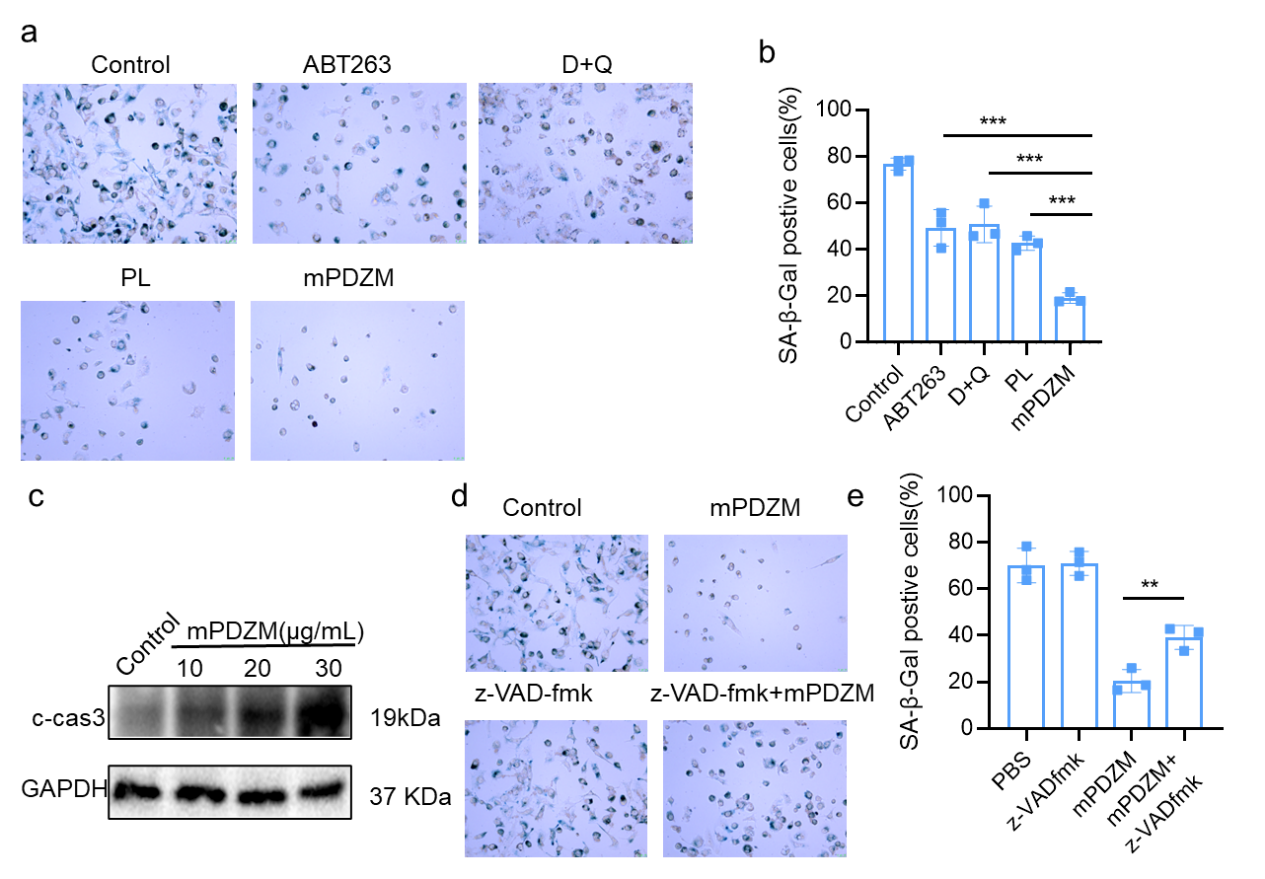


**Figure S17.** **Mechanistic investigation of mPDZM-mediated senescent cell clearance**. (a) SA-β-Gal staining images of Hepa1-6 senescent cells after treatment with PBS, free PL, D+Q (Dasatinib + Quercetin), ABT263, or mPDZM. (b) Quantification of SA-β-Gal-positive cells. (c) the expression of cleaved-caspase3 in senescent cells treated with indicated concentration of mPDZM. (c-d) SA-β-Gal staining images and quantification of mPDZM treated Hepa1-6 senescent cells with or without z-VAD-fmk pretreatment.


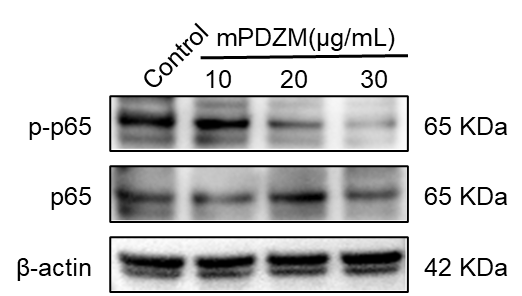


**Figure S18.** **mPDZM suppresses NF-κB signaling activation**. Western blot analysis was performed to examine the expression levels of phosphorylated p65 (p-p65) and total p65 in Hepa1-6 cells treated with the indicated concentrations of mPDZM.


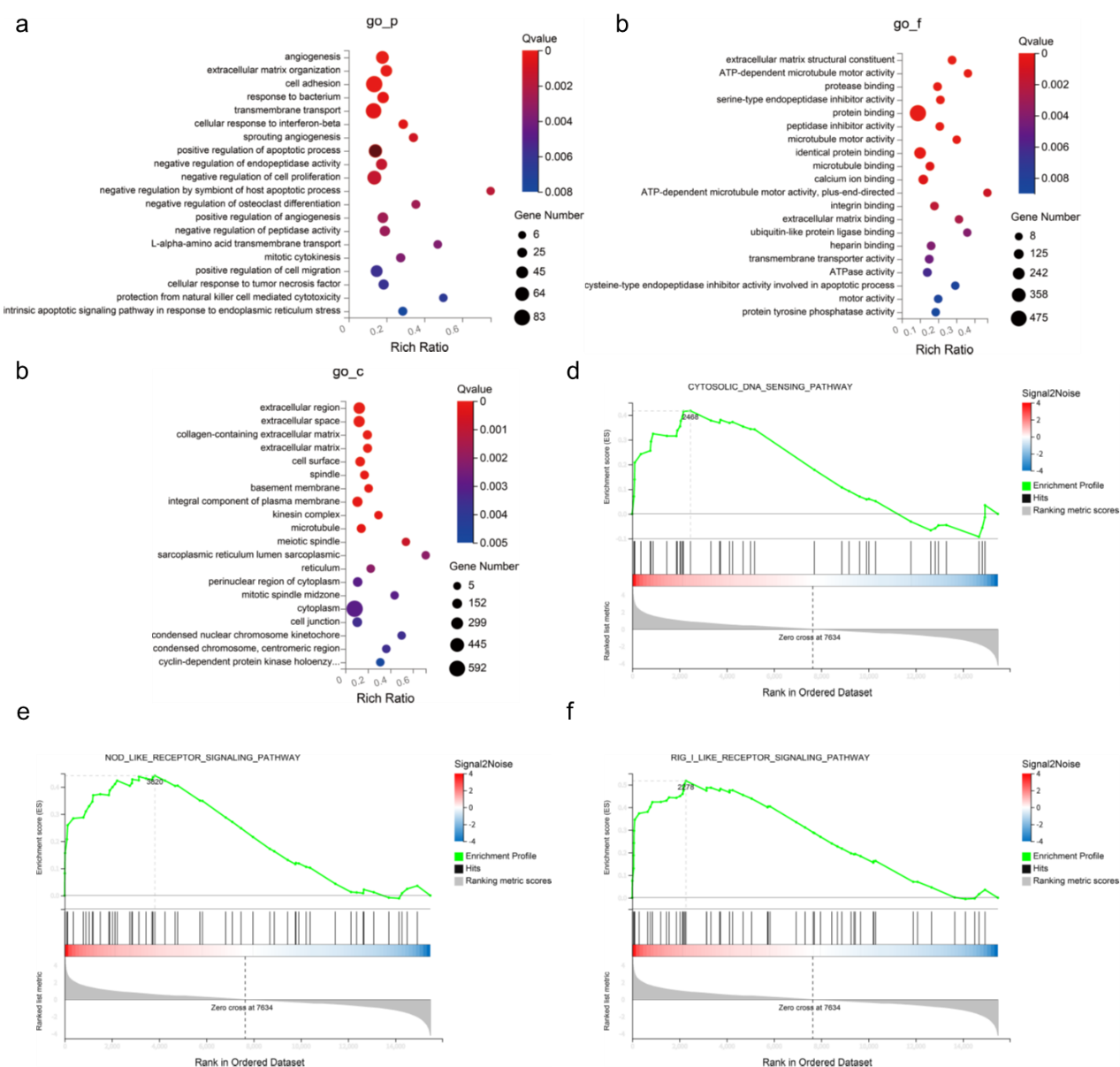


**Figure S19.** GO enrichment analysis and GSEA analysis of differentially expressed genes (DEGs) between mPDZM-treated and PBS-treated Hepa1-6 cells. (a) Biological process (BP) category. (b) Molecular function (MF) category. (c) Cellular component (CC) category. GSEA analysis revealed significant activation of the NOD-like receptor signaling pathway, the RIG-I-like receptor signaling pathway, and the cytosolic DNA sensing pathway.

**Figure S20. The release of CXCL10 in cells with indicated treatments.** ELISA assay was performed to tested the CXCL10 release. Statistical analysis was performed using one-way ANOVA with Tukey’s post hoc test, **p* < 0.05, ***p* < 0.01, ****p* < 0.001.


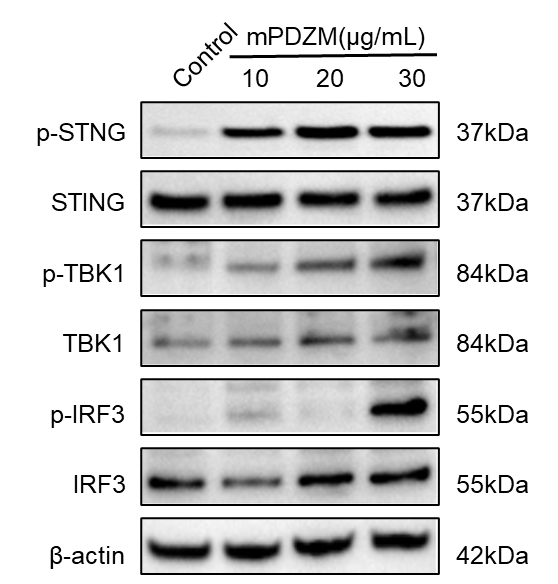


**Figure S21.** The activation of STING pathway in DCs. Western blot analysis was performed to examine the expression levels of phosphorylated STING (p-STING), p-TBK1, p-IRF3 in Hepa1-6 cells treated with the indicated concentrations of mPDZM.


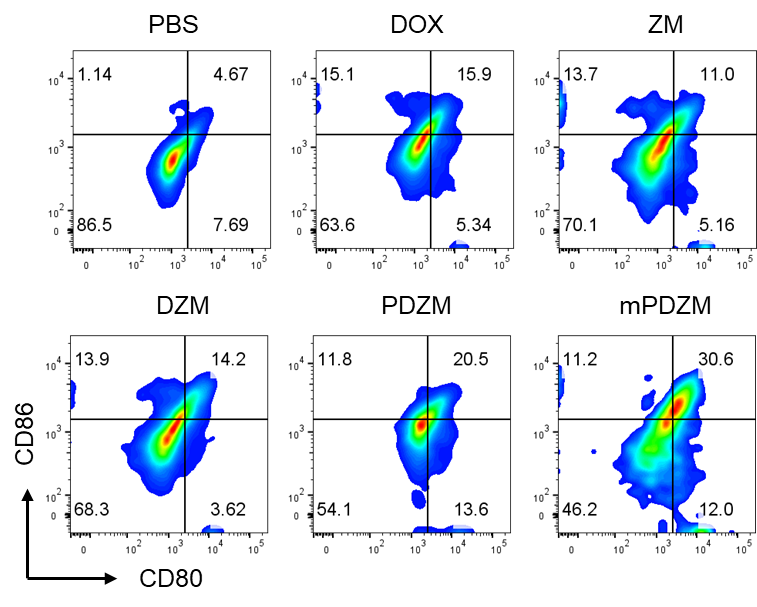


**Figure S22.** Flow cytometric analysis of matured DC. Representative dot plots showing the CD80^+^CD86^+^ DC in co-cultured BMDCs and Hepa1-6 cells pretreated with PBS, DOX, ZM, DZM, PDZM, or mPDZM.


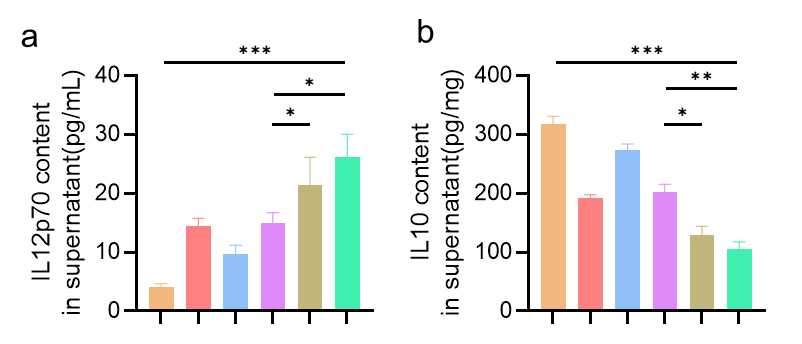


**Figure S23.** ELISA assays measuring IL-12p70 and IL-10 levels in the culture supernatants from co-cultured BMDCs and Hepa1-6 cells pretreated with PBS, DOX, ZM, DZM, PDZM, or mPDZM.


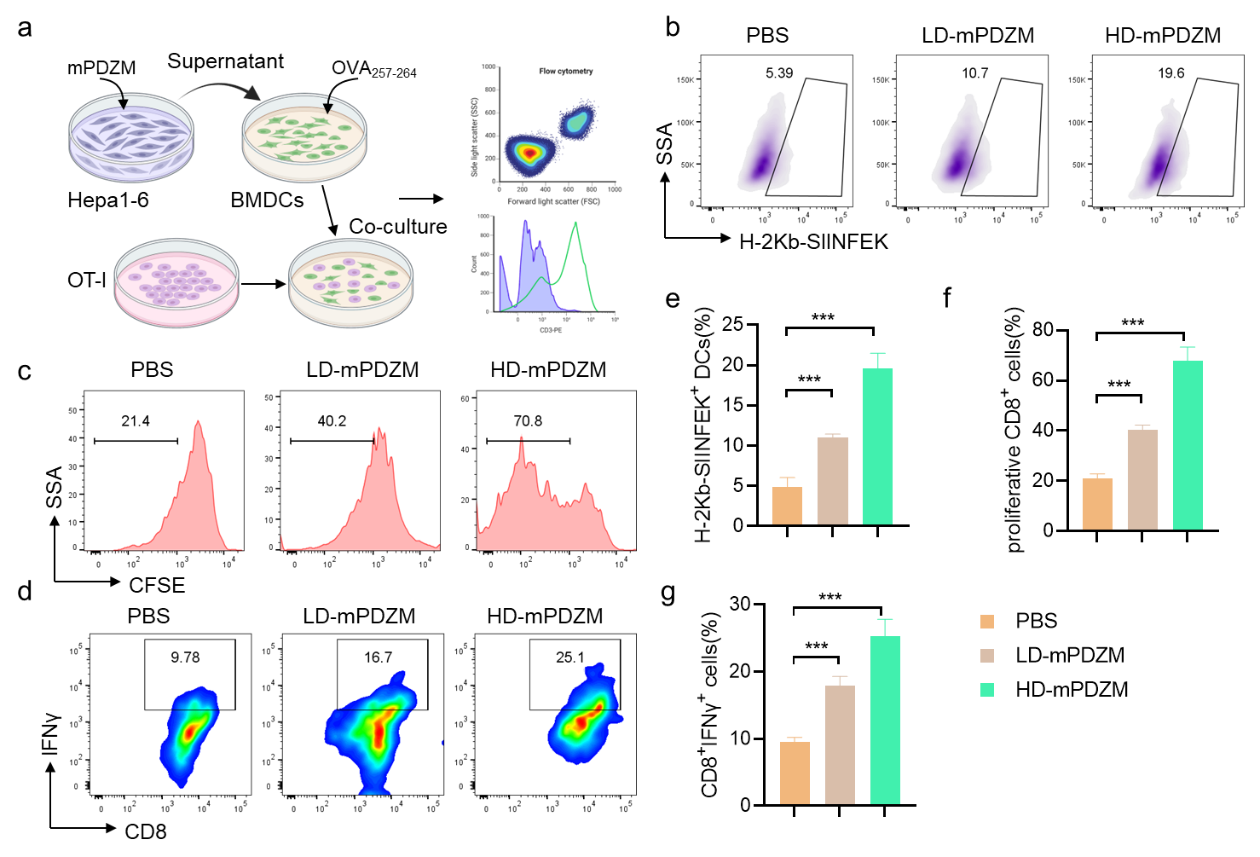


**Figure S24.** **mPDZM enhances antigen cross-presentation and promotes antigen-specific CD8⁺ T-cell activation**. (a) Schematic illustration of the experimental workflow. Hepa1-6 cells were treated with PBS, 15 μg/mL, or 30 μg/mL, and cell supernatants were collected to stimulate OVA_257-264_ pulsed BMDCs, followed by coculture with CD8^+^ T cells. (b) Flow cytometry analysis revealed the expression of H-2KbSIINFEKL in BMDCs. (c) Representative CFSE dilution histograms of CD8^+^ T cells in each treatment group. (d) Representative flow cytometry plots of IFN-γ^+^CD8^+^ T cells. (e) Quantification of H-2KbSIINFEKL^+^ BMDCs, (f) T-cell proliferation, and (h) IFN-γ^+^CD8^+^ T cells.


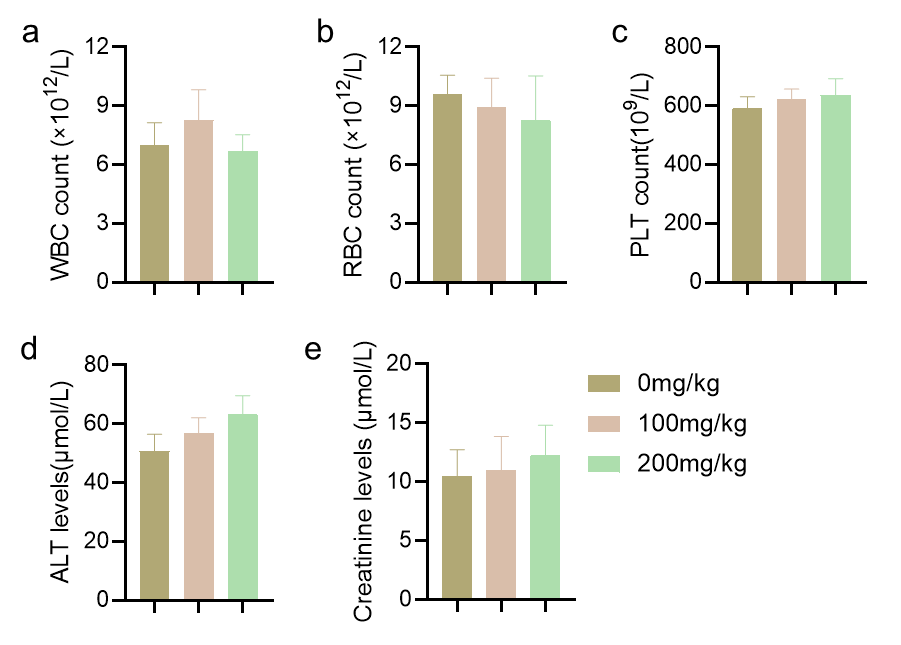


**Figure S25. Hematological parameters.** Mice were administered the indicated doses (0, 100, and 200 mg/kg), and blood samples were collected for hematological analysis. (a) White blood cell count (WBC), (b) red blood cell count (RBC), (c) platelet count (PLT), (d) alanine aminotransferase (ALT), and (e) Creatinine (CR). Data are presented as mean ± SD. Statistical significance was determined by one-way ANOVA followed by Tukey’s post hoc test.


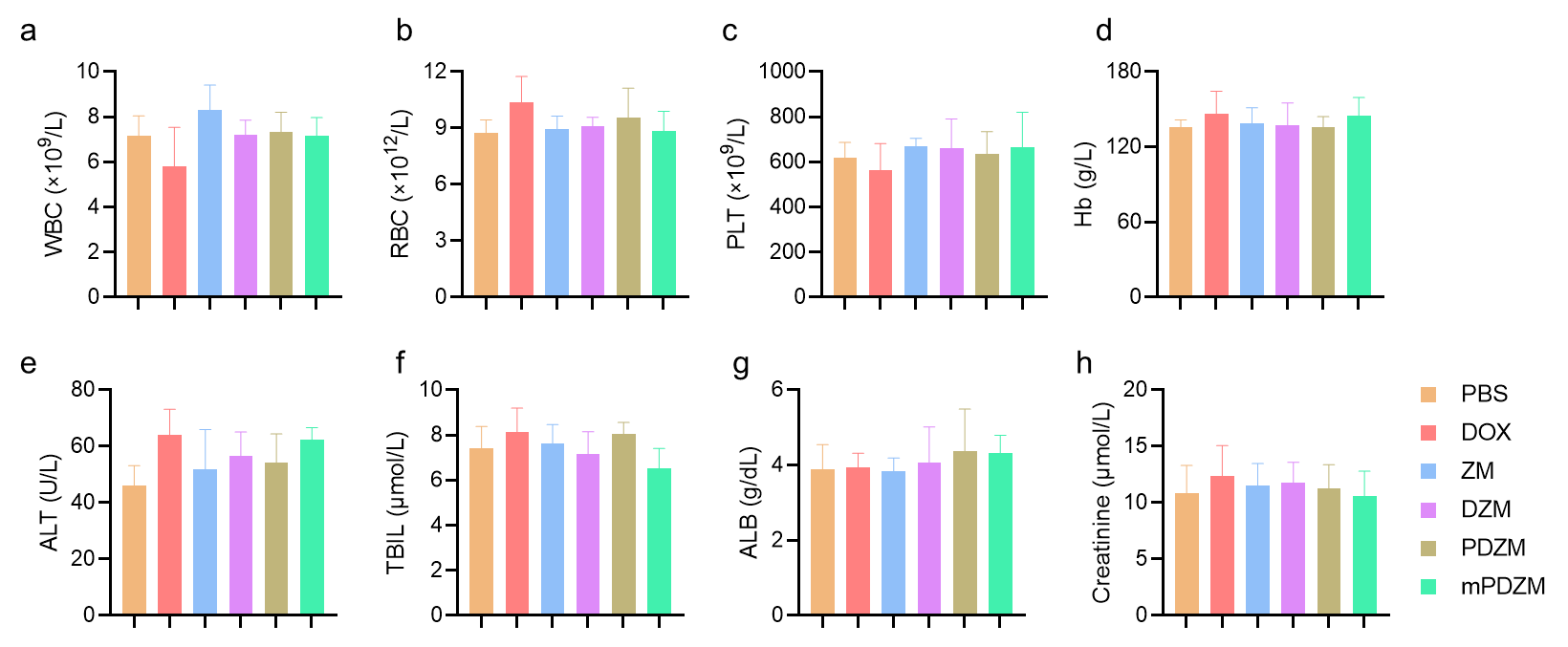


**Figure S26. Hematological parameters.** Hematological analysis of mice treated with PBS, DOX, ZM, DZM, PDZM, and mPDZM.


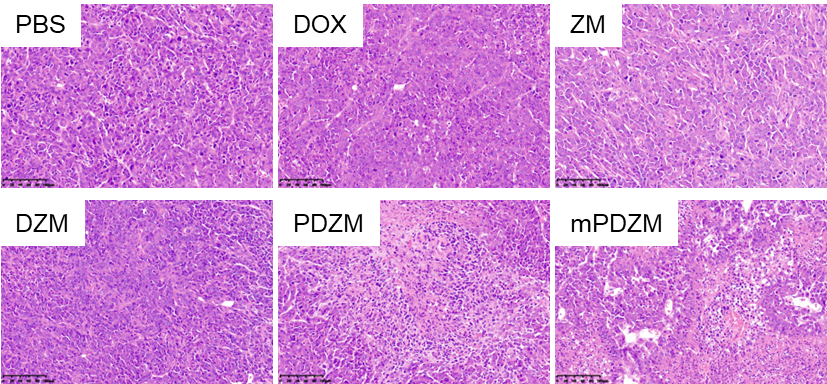


**Figure S27.** Histopathological examination of tumor tissues after different treatments. Hematoxylin and eosin (H&E) staining of tumor sections from Hepa1-6 tumor-bearing mice treated with PBS, DOX, ZM, DZM, PDZM, or mPDZM. Scale bars: 100 μm.


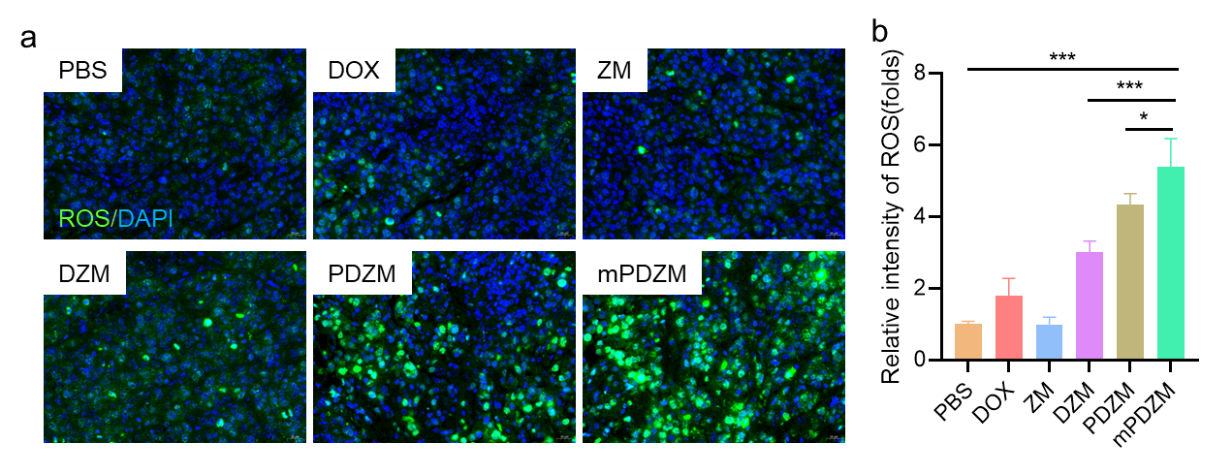


**Figure S28.** Immunofluorescence analysis of ROS generation in tumor tissues. (a)Representative immunofluorescence images showing ROS production (green) in tumor sections from Hepa1-6 tumor-bearing mice treated with PBS, DOX, ZM, DZM, PDZM, or mPDZM. ROS was detected using the DCFH-DA probe. Nuclei were counterstained with DAPI (blue). (b) Quantification of ROS fluorescence intensity. Data are presented as mean ± SD. Statistical analysis was performed using one-way ANOVA with Tukey’s post hoc test, **p* < 0.05, ***p* < 0.01, ****p* < 0.001.


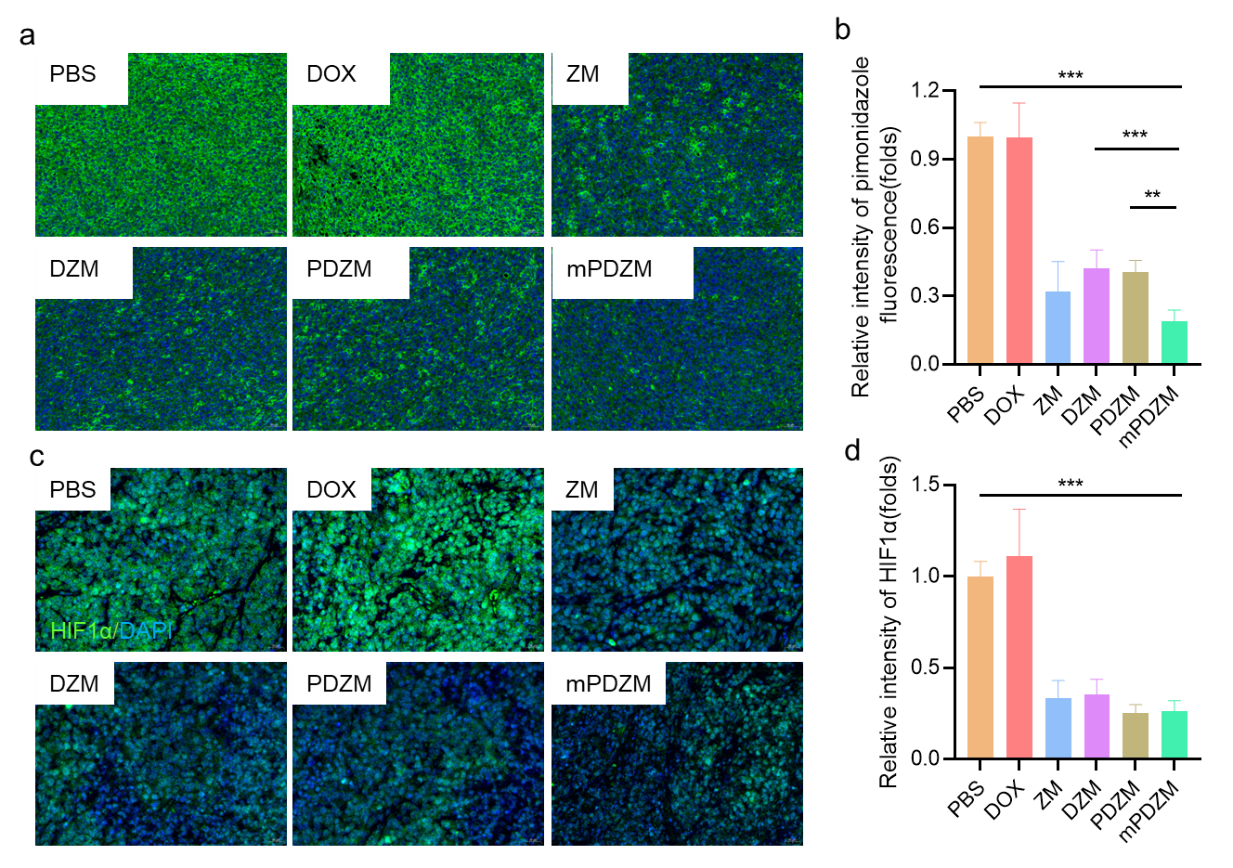


**Figure S29.** Immunofluorescence analysis of tumor hypoxia alleviation. (a) Representative immunofluorescence images of pimonidazole adducts (green) in tumor sections from Hepa1-6 tumor-bearing mice treated with PBS, DOX, ZM, DZM, PDZM, or mPDZM. Nuclei were counterstained with DAPI (blue). (b) Quantification of pimonidazole fluorescence intensity. (c) Representative immunofluorescence images of HIF-1α (green) in tumor sections. Nuclei were counterstained with DAPI (blue). (d) Quantification of HIF-1α fluorescence intensity. Data are presented as mean ± SD. Statistical analysis was performed using one-way ANOVA with Tukey’s post hoc test, **p* < 0.05, ***p* < 0.01, ****p* < 0.001.


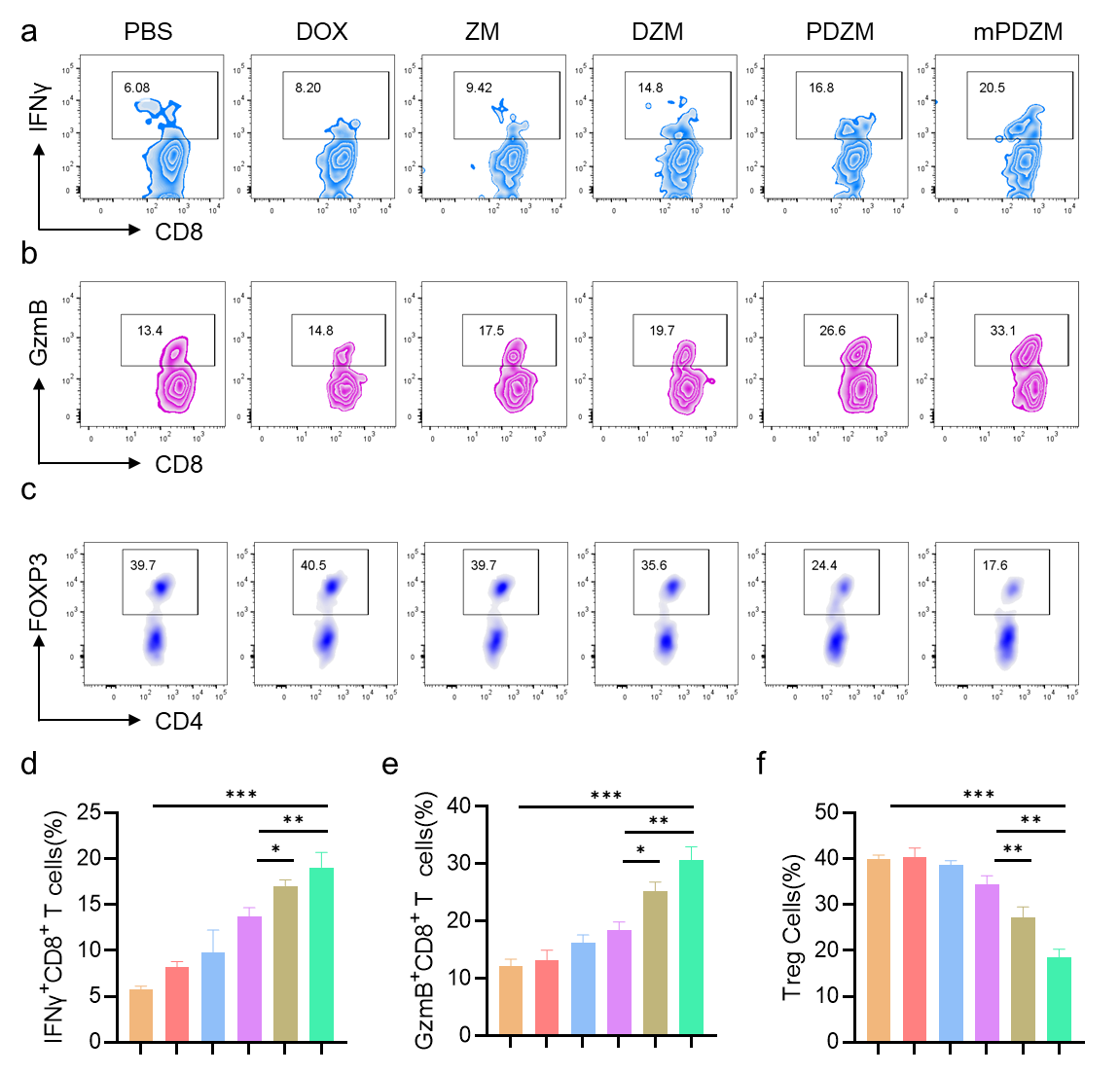


**Figure S30.** Flow cytometric analysis of tumor-infiltrating immune cell subsets in tumors. (a) Representative flow cytometry plots showing IFNγ^+^CD8^+^ T cells in tumors. (b) Representative flow cytometry plots showing GramzB^+^CD8^+^ T cells in tumors. (c) Representative flow cytometry plots showing regulatory T cells (Tregs, CD4^+^ Foxp3^+^) in tumors. (d) Quantification of IFNγ^+^CD8^+^ T cells in tumors. (e) Quantification of GramzB^+^CD8^+^ T cells in tumors. (f) Quantification of CD4^+^ Foxp3^+^ T cells in tumors. Data are presented as mean ± SD. Statistical analysis was performed using one-way ANOVA with Tukey’s post hoc test, **p* < 0.05, ***p* < 0.01, ****p* < 0.001.


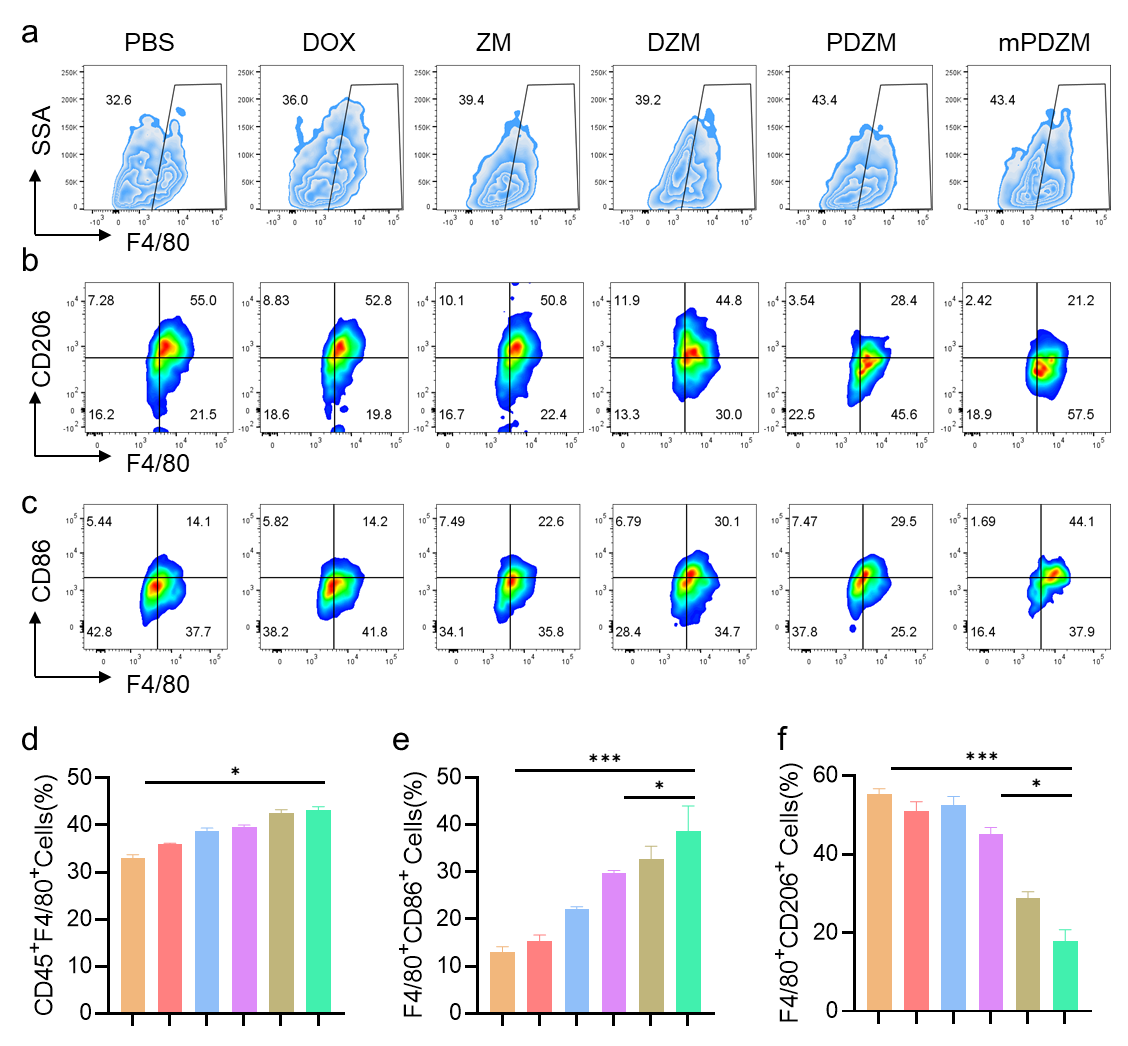


**Figure S31.** Tumor-associated macrophage phenotypes in tumors. (a) Representative flow cytometry plots showing tumor-associated macrophage (TAMs, F4/80^+^) in tumors. (b) Representative flow cytometry plots showing M2-like macrophages (F4/80^+^CD206^+^) in tumors. (c) Representative flow cytometry plots showing M1-like macrophages (F4/80^+^CD86^+^) in tumors. (d) Quantification of TAMs in tumors. (e) Quantification of M2-like macrophages in tumors. (f) Quantification of M1-like macrophages in tumors. Data are presented as mean ± SD. Statistical analysis was performed using one-way ANOVA with Tukey’s post hoc test, **p* < 0.05, ***p* < 0.01, ****p* < 0.001.


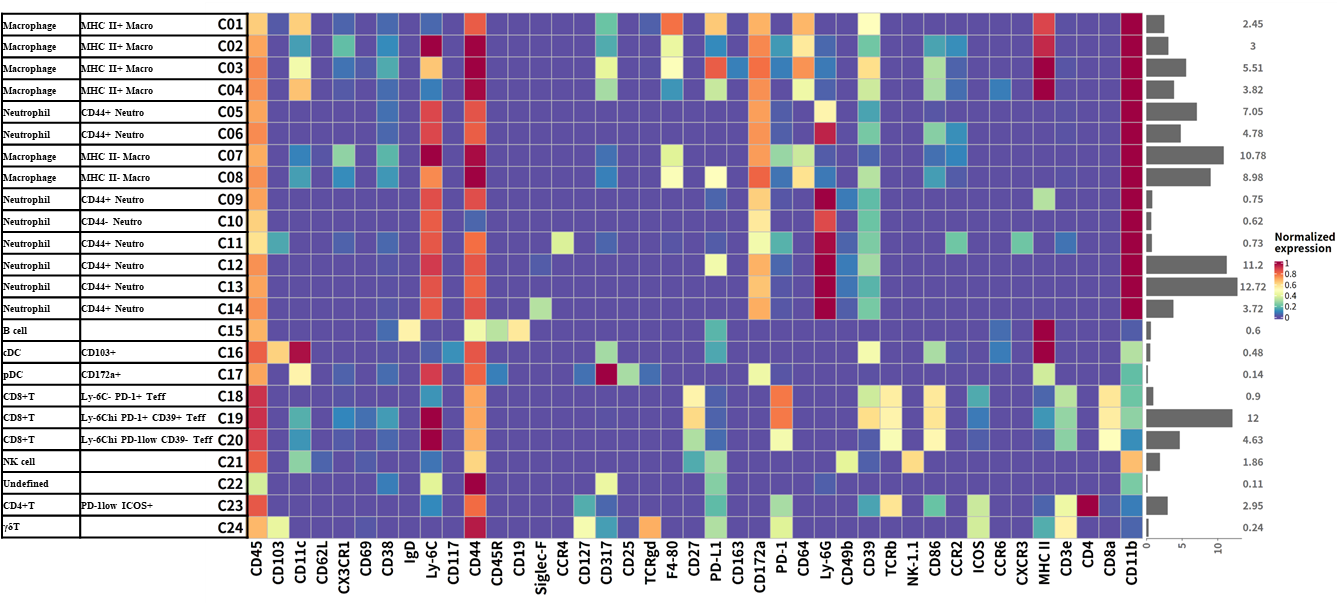


**Figure S32.** Heatmap of marker expression profiles across 24 immune cell clusters identified by CyTOF. The heatmap displays the normalized expression levels of 35 lineage- and function-associated immune markers across 24 clusters (C01–C24) obtained by unsupervised clustering of tumor-infiltrating immune cells. Rows represent immune cell clusters, and columns represent CyTOF markers covering T-cell, NK-cell, B-cell, dendritic-cell, macrophage/monocyte, and myeloid activation signatures. Warmer colors (red/yellow) indicate higher expression, while cooler colors (blue) indicate lower expression.


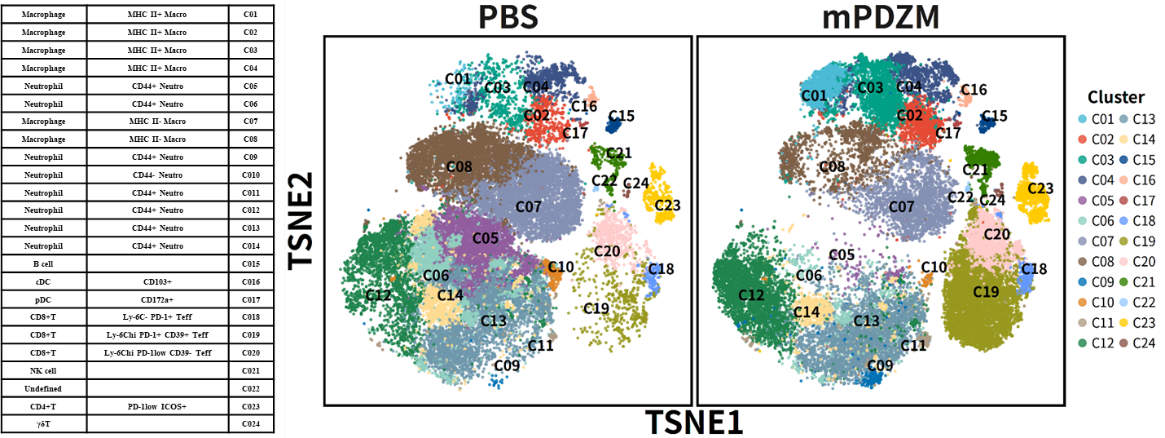


**Figure S33.** **t-SNE visualization of tumor-infiltrating immune cell populations in PBS- and mPDZM-treated mice.** t-SNE plots display the distribution of 24 immune cell clusters (C01–C24) identified by CyTOF profiling of tumor tissues from PBS and mPDZM treatment groups.


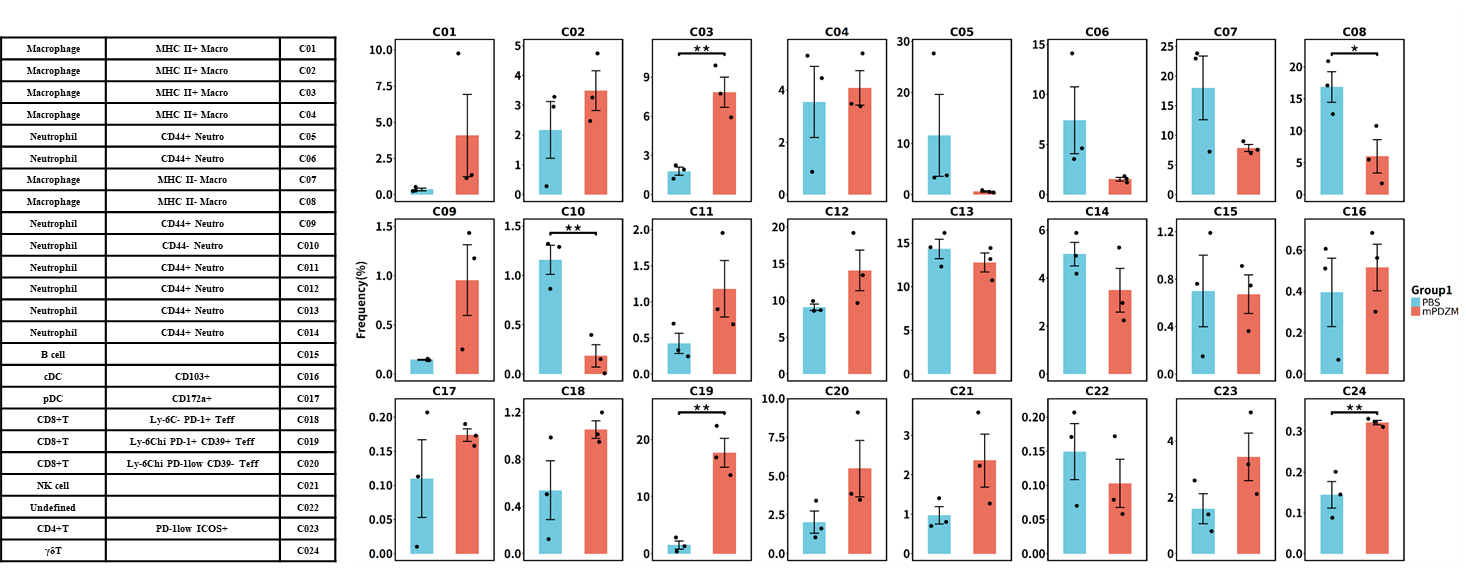


**Figure S34**. Quantitative comparison of immune cell cluster frequencies in tumors from PBS- and mPDZM-treated mice. Bar graphs display the relative abundance of 24 immune cell clusters (C01–C24) identified by CyTOF profiling of tumor-infiltrating immune cells.


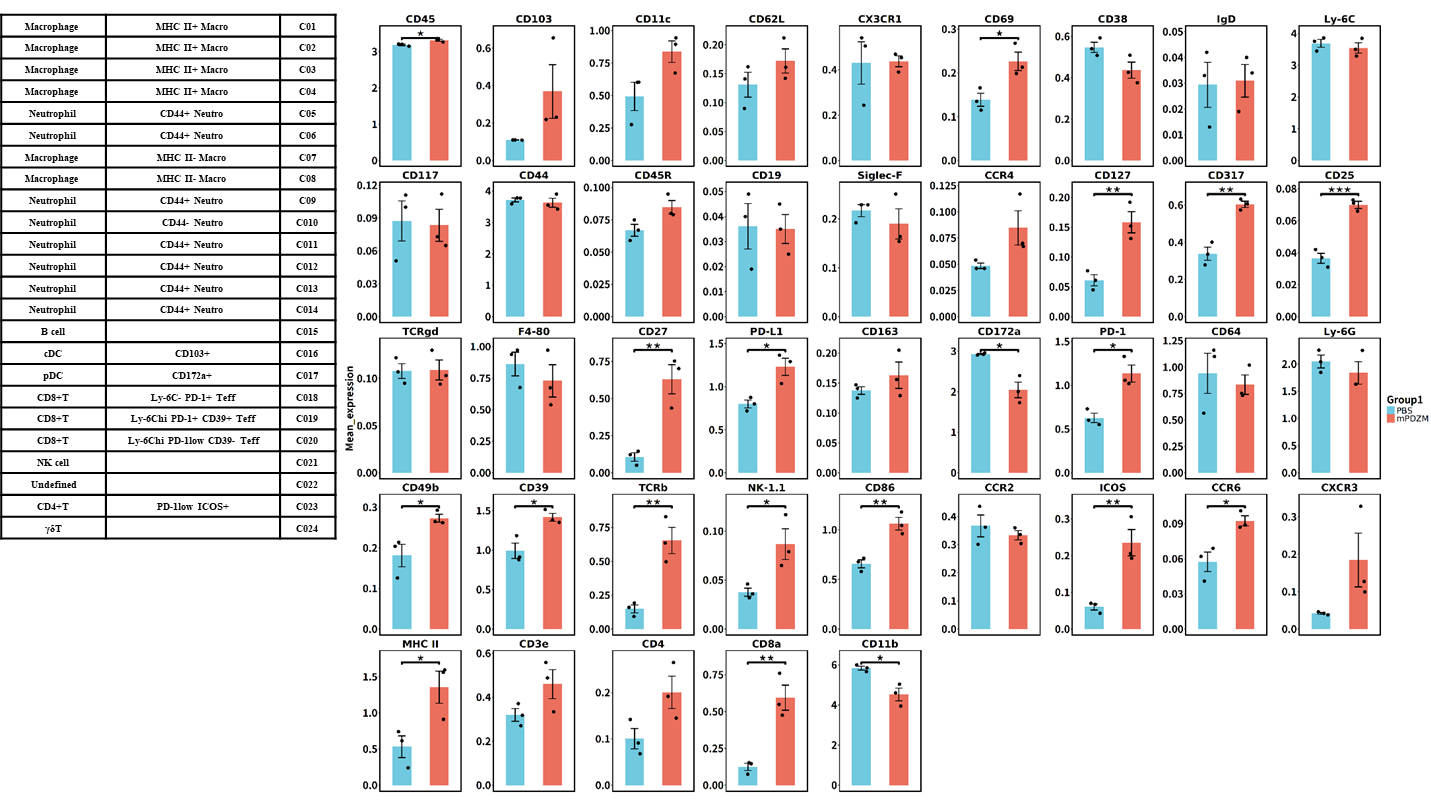


**Figure S35.** **Differential expression of immune-related markers in PBS- and mPDZM-treated tumors.** Bar plots show the normalized expression levels of key lineage, activation, co-stimulatory, co-inhibitory, chemokine, and antigen-presentation markers across tumor-infiltrating immune cells from PBS (blue) and mPDZM (red) treatment groups.


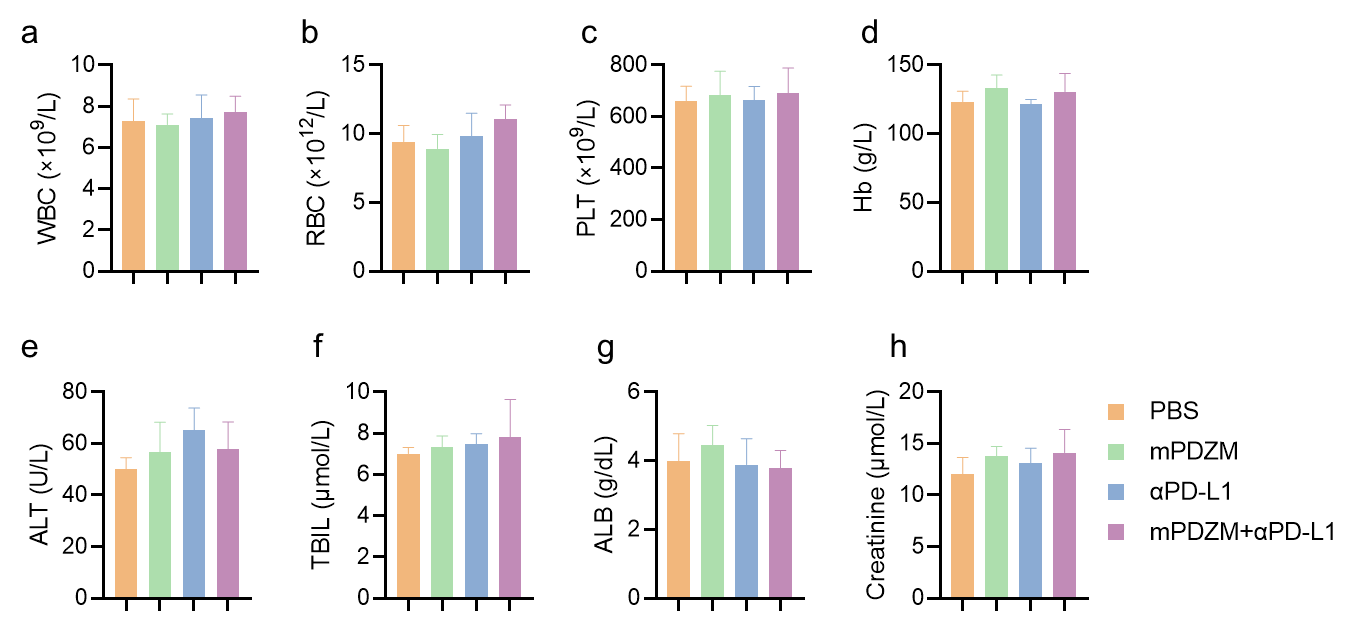


**Figure S36.** Hematological analysis of mice treated with PBS, anti-PD-L1, mPDZM, and mPDZM+ anti-PD-L1.


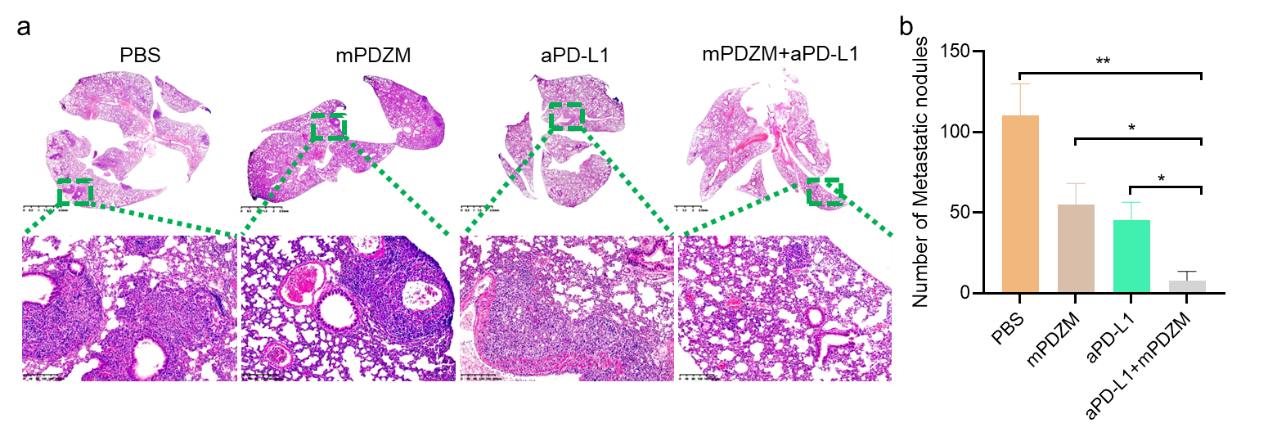


**Figure S37. mPDZM treatment reduces lung metastasis burden *in vivo*.** (a) H&E-stained lung sections from mice treated with PBS, mPDZM, anti-PD-L1 and anti-PD-L1+mPDZM. (b) Quantification of metastatic nodules confirmed a significant reduction in lung metastasis in each group. Data are presented as mean ± SD; statistical significance was determined using a two-tailed unpaired Student’s t-test. *p < 0.05, **p < 0.01.


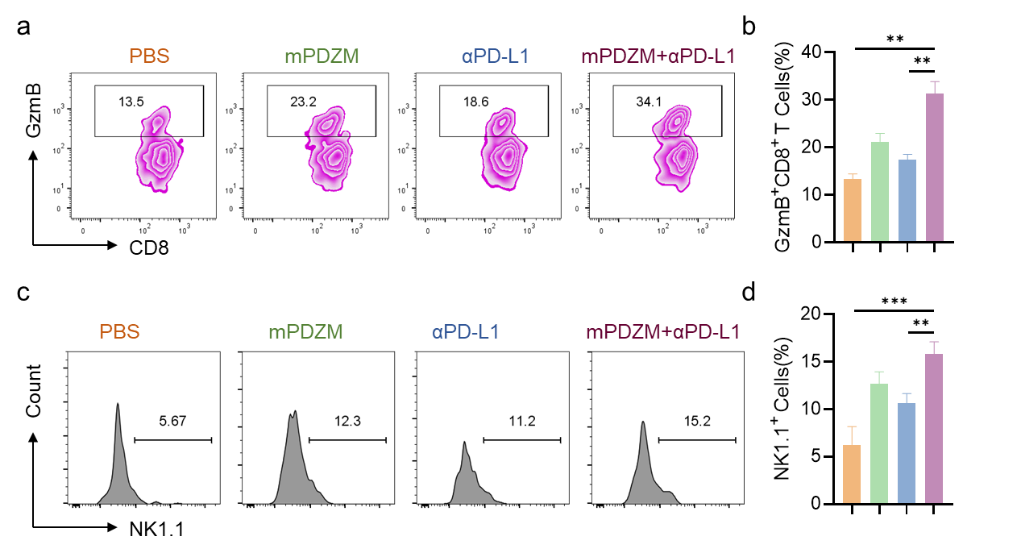


**Figure S38. GzmB^+^ CD8⁺ T cells and NK cells in distant tumors following mPDZM and anti-PD-L1 combination therapy.** (a) Representative flow cytometry plots and (b) quantitative analysis of granzyme B (GzmB) expression in CD8⁺ T cells isolated from distant tumors. (c) Representative flow cytometry histograms and (d) quantitative analysis of NK1.1⁺ natural killer (NK) cells in distant tumors. Data are presented as mean ± SD. Statistical analysis was performed using one-way ANOVA with Tukey’s post hoc test, **p* < 0.05, ***p* < 0.01, ****p* < 0.001.


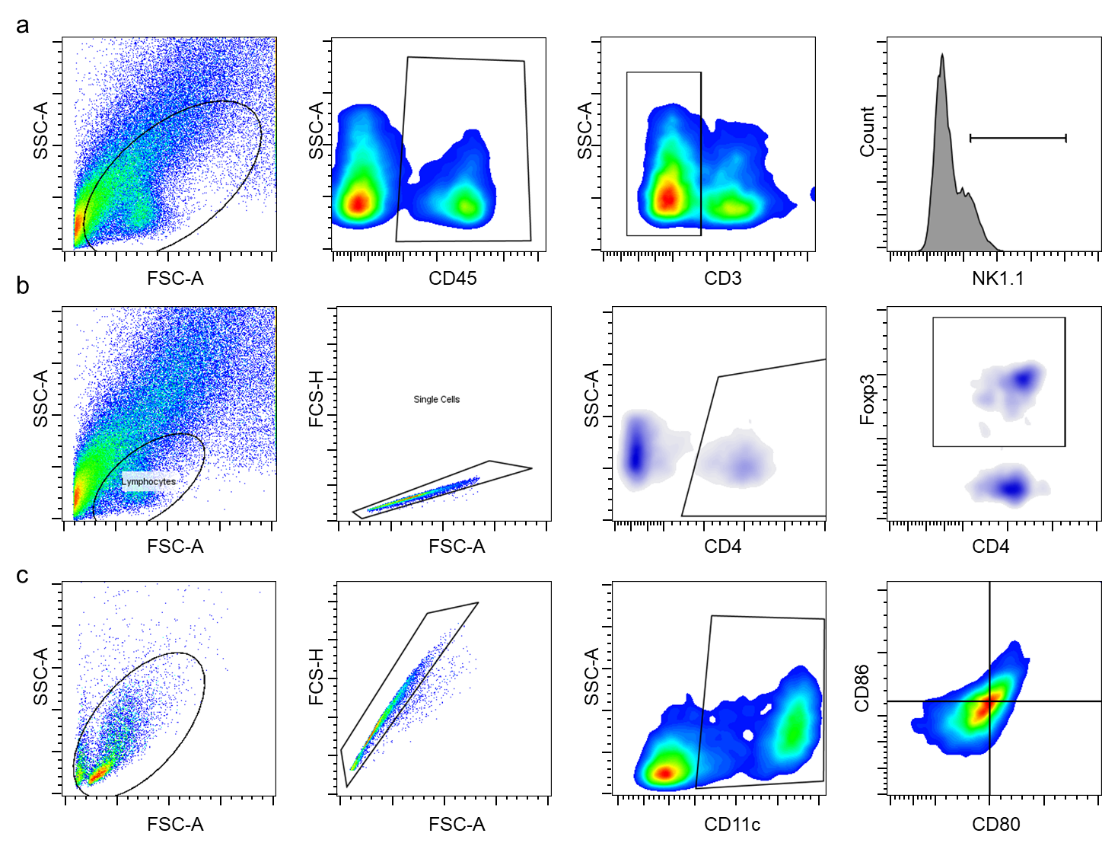


**Figure S39**. **Gating strategy for flow cytometric analysis of immune cell subsets.**
Gating workflow used to identify major immune cell populations within tumor tissues. Gating strategy for (a) NK cells and (b) Treg in tumors. (c) Gating strategy for DCs in TDLN.
